# Supplementary material for: Study protocol to examine the effects of acute exercise on motor learning and brain activity in children with developmental coordination disorder (ExLe-Brain-DCD)
Source: PLoS One. 2024 May 9;19(5):e0302242. doi: 10.1371/journal.pone.0302242 (PMC11081356; doi:10.1371/journal.pone.0302242)
Supplement: S1 File — (PDF) [file pone.0302242.s002.pdf]

EXERCISE LEARNING PROJECT

Enhancing learning via exercise in children with Developmental Coordination Disorder (DCD)

Dr. Albert Busquets Faciabén (PI)

Dr. Rosa Angulo Barroso (PI)

Dr. Blai Ferrer Uris

Index

Abstract…… 1

Introduction……….. 2

Justification and hypothesis... 13

Objectives……... 15

Methods……….. 16

Work plan and scientific roll of each collaborator……………………………………………………………. 25

Scientific and social interest……………………………………………………………………………………………. 28

References…….. 32

Annex 1 (researchers’ CV) *………………………………………………………………………………………….. 45

Annex 2 (schools) *…………………………………………………………………………………………………… 46

Annex 3 (parents/legal tutor informed consent)……………………………………………………………..… 47

Annex 4 (children informed assent)……………………………………………………………………………… 60

Annex 5 (questionnaires)……………………………………………………………………………………………. 69

* This part is not included because it contains personal data and it is not relevant for revision aspects

ABSTRACT

Developmental coordination disorder (DCD) is one of the most prevalent chronic conditions that affects about 35,000 children in Catalunya alone. Yet, it is not particularly known. DCD is a disorder characterized by a significant delay in motor skill performance and learning which carries functional consequences in daily activities and academic performance. Without proper and timely diagnosis and intervention, these consequences may persist until adulthood. Physical activity, as a potential intervention, has been proven to improve motor learning (adaptation and consolidation) in children, even in those with some disorders. However, no studies have examined the effect of a short bout of physical exercise on motor adaptation and consolidation in children with DCD. Furthermore, the potential role of perceptual-motor integration and attention as mediators of learning has been examined via neuroimaging in pediatric populations, but not in children with DCD. Therefore, the primary aims of this project will be (a) to examine the effect of acute exercise on motor learning (adaptation and consolidation) while performing a rotational visuo-motor adaptation task (rVMA) in children with and without DCD , (b) to explore cortical activation in the visuomotor integration and the attentional network areas while learning the rVMA task in children with and without DCD under rest or post-exercise conditions, and (c) to explore via a pilot study the combined use of two optical neuroimaging techniques (functional near-infrared spectroscopy, fNIRS, and functional diffuse correlation spectroscopy, fDCS) to measure cortical activation in in the visuomotor integration and the attentional network areas while learning a motor adaptation task in children with and without DCD under rest or post-exercise conditions. We will recruit 120 children from 6-8 schools (60 DCD, 60 controls) and randomly assign them to either exercise or rest prior to performing a rotational visuo-motor adaptation task. The exercise will be a 13min shuttle run task. Adaptation and consolidation of the learned task will be evaluated via two error variables and 3 retention tests (1h, 24h and 7 days post adaptation). Cortical activation will be registered via fNIRS during the baseline, adaptation and consolidation phases to estimate sustained attention. As a pilot study, the brain activity of a subset of 10 children of each group will be recorded using a hybrid optical neuroimaging device (fNIRS + fDCS). We expect to find exercise benefits on both, motor learning and attention, in children with DCD so that their profiles are closer to those of children with typical development. The results of this project will provide further evidence to: (a) better characterize children with DCD; (b) establish acute exercise as a potential intervention to improve motor learning and attention, explored for the first time through concurrent brain imaging and behavioral assessments in DCD children; (c) design educational materials for educators and families related to children with DCD; and (d) verify that combining optical technology provide us with the possibility to obtain more accurate and robust information of the children's brain activity during learning.

INTRODUCTION

DCD: Prevalence and functional consequences

Imagine how a child may feel when most peers can tie their shoes without problems but he cannot do it or is the last one to finish; or when at the playground she is the last one to be picked up to participate in a physical activity; or when most children are enjoying playing soccer but he chooses to stay quietly in a corner. Unfortunately, these situations are very common for a child with developmental motor coordination disorder (DCD). According to World Health Organization (1992), 5-6% of children in North America and Europe have these problems. Although prevalence estimates varies between 2-19% depending on the criteria used (Kadejso & Gillberg, 1999; Lingam, Hunt, Golding, Jongmans, & Emond, 2009; Tsiotra et al., 2006; Zwicker, Missiuna, Harris, & Boyd, 2012), the most widely accepted prevalence (6%) would imply that 34,971 children in Catalunya alone would be diagnosed with DCD (Censo del Instituto Nacional de Estadística, 2017).

World Health Organization (2018) has defined DCD as "a disorder characterized by a significant delay in the acquisition of gross and fine motor skills and impairment in the execution of coordinated motor skills that manifest in clumsiness, slowness, or inaccuracy of motor performance. Coordinated motor skills are substantially below that expected given the individual's chronological age and level of intellectual functioning. Onset of coordinated motor skills difficulties occurs during the developmental period and is typically apparent from early childhood. Coordinated motor skills difficulties cause significant and persistent limitations in functioning (e.g., in activities of daily living, school-work, and vocational and leisure activities)."

These motor coordination problems make daily activities like tying shoe laces and participation in physical activities, such as soccer, difficult and unappealing. As a consequence, a cascading effect takes place where physical inactivity leads to sedentary behaviors (Fong et al., 2011), which in turn leads to over-weight or obesity (Beutum, Cordier, & Bundy, 2013; Cairney, Hay, Faught, & Hawes, 2005; Faught, Demetriades, Hay, & Cairney, 2013), and therefore, lower physical fitness and higher risk of cardiovascular diseases (Cairney, Hay, Veldhuizen, & Faught, 2011; Cairney, Veldhuizen, King-Dowling, Faught, & Hay, 2017). This ill-sequence of events makes DCD one of the major health problems among children worldwide (Green, Baird, & Sugden, 2006). Furthermore, the motor behavior characteristics of children with DCD significantly interfere not only with daily activities (Summers, Larkin, & Dewey, 2008; Wang, Tseng, Wilson, & Hu, 2009), but also with academic performance (Dewey, Kaplan, Crawford, & Wilson, 2002), and ultimately with quality of life (Zwicker, Harris, & Klassen, 2013). In addition, the motor difficulties in children with DCD have also been linked to an increased risk for mental health issues, such as low self-esteem (Cairney et al., 2007; Poulsen, Ziviani, Johnson, & Cuskelly, 2008), social isolation (Katartzi & Vlachopoulos, 2011), higher anxiety (Piek, Dawson, Smith, & Gasson, 2008; Pratt & Hill, 2011) and depression (Dewey et al., 2002).

DCD: Comorbidity and severity

Although DCD is a unique and separate neurodevelopmental disorder, its manifestations are multiple and diverse producing large heterogeneity among those who receive the diagnosis (Visser, 2003). It is known that about 35-50% of children with DCD also have attention deficit hyperactivity disorder (ADHD) (Dewey et al., 2002; Gomez & Sirigu, 2015; Green et al., 2006), and 50% of dyslexic children also have DCD (Iversen, Berg, Ellertsen, & Tønnessen, 2005). However, for the purpose of the present line of research, children with probable DCD with or without comorbid ADHD or dyslexia will be the focus. This decision will allow us to characterize and study this population as it presents itself in most contexts.
In any case, the diagnosis of DCD cannot be explained by mental retardation, specific congenital or acquired neurological disorder (Blank, Smits-Engelsman, Polatajko, & Wilson, 2012). On the contrary, DCD should be determined by poor motor coordination that interferer with academic performance or activities of the daily life. The Motor Assessment Battery for Children (M-ABC2, Henderson, Sugden, & Barnett, 2007; B. N. Wilson et al., 2009) and specific check-lists and questionnaires are used to determine DCD severity. The M-ABC2 is the most widely used and one of the two assessment batteries recommended by the European Academy of Childhood Disability guidelines for diagnosing DCD (Blank et al., 2012). Although several DCD subtypes have been proposed (Visser, 2003), children who show motor coordination delay below the 15th percentile on the M-ABC2 are classified as probable DCD and this classification is sufficient to investigate characterization of the population and preventive actions or interventions (Cairney, Hay, Veldhuizen, Missiuna, & Faught, 2010; Cairney et al., 2017; Chia, Licari, Guelfi, & Reid, 2014; Tsai, 2009). A more restrictive cutoff of below 5th percentile on the M-ABC2 is used for clinical purposes with these children being diagnosed with DCD (Blank et al., 2012; Gueze, Jongmans, Schoemaker, & Smits-Engelsman, 2001).

Exercise interventions in DCD: Chronic versus Acute

Because DCD is a chronic condition that cannot be reversed and will continue until adulthood (Kirby, Sugden, Beveridge, & Edwards, 2008) preventive and intervention actions are crucial (Missiuna & Campbell, 2014). In fact, many have proposed that a child with a diagnosis of DCD should be treated and that any treatment is better than no treatment (Blank et al., 2012; Hillier, 2007; Pless & Carlsson, 2000). Unfortunately, even when diagnosed, DCD often goes untreated (Plata & Guerra, 2009). In the last decade, studies examining the effect of different forms of chronic exercise on motor and cognitive performance of children with DCD have proliferated (for reviews see (Preston et al., 2017; Smits-Engelsman & Wilson, 2013).

In a recent systematic review, Preston et al., (2017) concluded that interventions that focused on neuro-motor, perceptual-motor or motor training were the most effective and provided the strongest evidence to support their use to improve children's movement skills. These motor intervention programs had few characteristics in common; they all were task-oriented and they used instrumentation. On the other hand, intervention programs based on aquatic therapy, table tennis or soccer were found potentially effective but lacked robust evidence; while Wii Fit training or Taekwondo were classified as ineffective interventions.

Most motor interventions to date have utilized chronic exercise, that is, a program repeated several times per week during several weeks, to examine changes in motor performance in children with DCD. However, none, to our knowledge, have used acute exercise to observe and understand motor improvements in this population. Why acute exercise might be important for children with DCD? By definition, acute exercise is short in duration but it is typically intense. The limited time requirement imposed by a short bout of exercise is clearly appealing if one thinks about implementing such strategy in the schools or in time-pressing situations as it happens very often nowadays. In fact, previous studies have shown that acute exercise provided benefits for children with ADHD in speed of processing and inhibitory control using the Stroop test (Piepmeier et al., 2015) and in appropriate response preparation using the Go/NoGo test (Chuang, Tsai, Chang, Huang, & Hung, 2015). Even in children with typical development, Lambrick, Stoner, Grigg, & Faulkner (2016) demonstrated that a 30 minute single session of either continuous or intermittent exercise at various intensities yielded better inhibitory control compared to no exercise in children at 9 years of age. Taken together, it seems reasonable to think that acute exercise may also benefit children with DCD but no studies, to our knowledge, have examined this question.

Motor Learning and DCD

In spite of the intervention program used, improvements in motor skill require a learning process. An important and relatively recent new addition to the criteria for diagnosing DCD (Blank et al., 2012) acknowledges this fact. Problems involved in the acquisition (that is, learning), not only the execution, of coordinated motor skills is now a critical characteristic in the identification of children with DCD. Therefore, it seems reasonable to examine the effect of exercise intervention programs on this particular ability (motor learning). Learning a new motor coordination skill entails the integration of motor and sensory information in the first place, so adequate timing of actions can take place. Surprisingly, little research is available that focused on how children with DCD learn new motor skills and the evidence is inconclusive given that consolidation and transfer of the learning has been weakly examined (Smits-Engelsman, Jelsma, Ferguson, & Geuze, 2015).

Two different laboratory methodologies have been used to examine motor learning in the literature: motor sequence learning and motor adaptation tasks (Bo & Lee, 2013). We will review each one separately. When examining the evidence related to sequence learning in children with DCD, some studies found group differences (Gheysen, Van Waelvelde, & Fias, 2011) while others did not (Lejeune, Catale, Willems, & Meulemans, 2013; P. H. Wilson, Maruff, & Lum, 2003). As pointed out by P. H. Wilson, Ruddock, Smits-Engelsman, Polatajko, & Blank (2013) lack of group differences between children with DCD and children with typical development (TD) in motor sequence learning is due to studies focusing solely on the fast acquisition stage of learning, disregarding the later stages where consolidation of the task is achieved.

Although some controversy exists, the most recent and better designed research examining motor sequence learning in children with DCD concluded that these children have problems learning a motor sequence (Gheysen et al., 2011). Potentially, this deficiency is related to the problems children with DCD demonstrate in activities of daily living which typically are

composed by a sequence of actions. In addition, these authors suggested that children with DCD relied more on a "look-then-move" strategy (increasing time for planning) rather than controlling their movements using a feedforward mechanism (that is, prediction).

Recently, a plausible hypothesis has been put forward to explain impaired motor learning in children with DCD. This hypothesis suggests a **deficit in the generation of an internal model** (Figure 1) so that children with DCD have a reduced ability to use predictive (feedforward) motor control (Adams, Lust, Wilson, & Steenbergen, 2014; Wilmut & Wann, 2008; P. H. Wilson & Butson, 2007; P. H. Wilson et al., 2013; Zwicker et al., 2012).

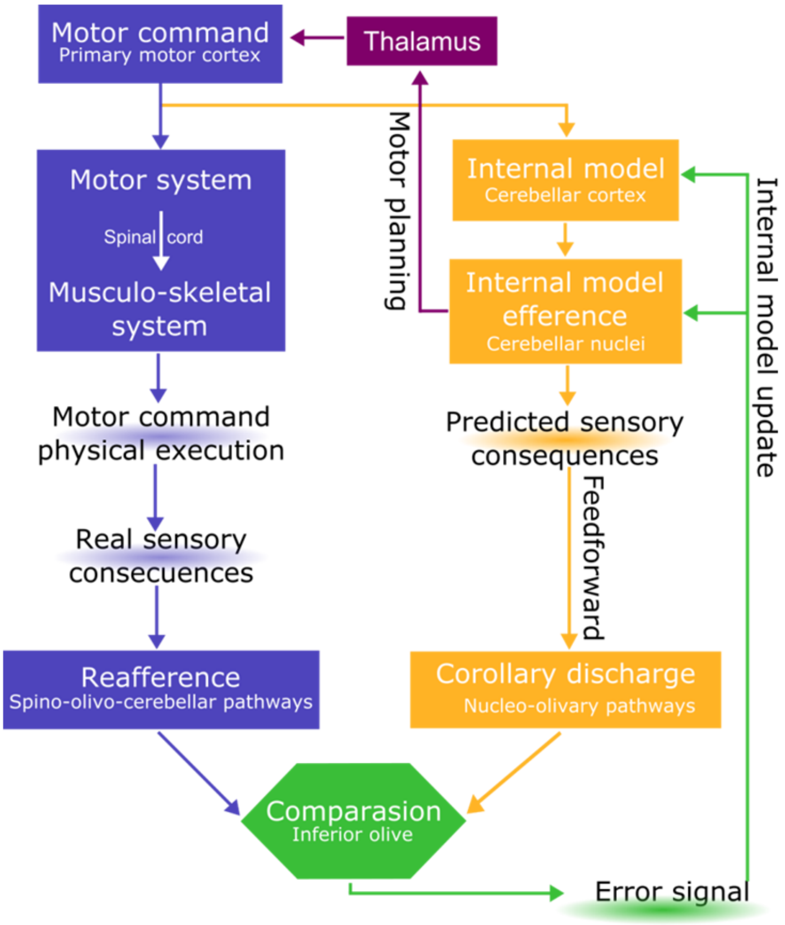

Figure 1. Theoretical and neural organization of motor control. The internal model is used to generate motor commands in the motor cortex (blue circuit), at the same time, an efferent copy is created in the cerebellum (orange circuit). Predicted sensory consequences (feedforward) from the efferent copy are compared with the real sensory consequences to detect errors (green circuit) that will be send as feedback and use to update the internal model.

Successful motor control is thought to result from an internal model that predicts sensory consequences of a motor command. The cerebellum is thought to receive an efferent copy of the motor command and then compare the predicted movement with the actual movement; if there is a mismatch, the cerebellum sends an error signal as feedback to create a more accurate movement on subsequent occasions (Kawato, 1999). The involvement of the cerebellum has been suspected in DCD (Bo, Block, Clark, & Bastian, 2008; Zwicker, Missiuna, & Boyd, 2009), which has since been confirmed in fMRI studies that show under activation of the

cerebellum in children with DCD relative to children with TD (Debrabant, Gheysen, Caeyenberghs, Van Waelvelde, & Vingerhoets, 2013; Zwicker, Missiuna, Harris, & Boyd, 2011). Cerebellar network—connections to frontal and parietal areas—also seem to be implicated in DCD, providing indirect support for the internal modeling hypothesis (Debrabant et al., 2013; Kashiwagi, Iwaki, Narumi, Tamai, & Suzuki, 2009; Zwicker, Missiuna, Harris, & Boyd, 2010; Zwicker et al., 2011).

The other widely used laboratory methodology to examine motor learning is **motor adaptation tasks**. Motor adaptation tasks have been used as a technique to examine the ability to form and adapt an internal model and therefore use a feedforward mechanism of motor control (Kasuga, Telgen, Ushiba, Nozaki, & Diedrichsen, 2015). Two types of motor adaptation tasks are usually used: tracking tasks, where participants have to follow a moving object with movements of their fingers, hand or arm; and visuomotor adaptation tasks, where participants have to perform discrete reaching movements to one or various targets. In both tasks a perturbation is introduced either on the participant's visual field (using prism glasses or screen rotations) (for an example see: Miall, Jenkinson, & Kulkarni, 2004) or on the participant's proprioceptive feedback (e.g. using force fields normally applied by robotic arms) (for an example see: Takahashi et al., 2006), causing a mismatch between visual and proprioceptive information. Therefore, participants have to adapt their movements to the introduced perturbation (perceptual recalibration), possibly using feedforward mechanisms (Henriques & Cressman, 2012). In particular, the task that is going to be used in this research project is a task known as the rotational Visuomotor Adaptation Task (rVMA) (Figure 2 Left). In the rVMA, participants use a joystick to control the movements of a screen cursor, which they have to move to reach to one of several target points, one at a time. A visual-motor mismatch between joystick movement and screen cursor movement is applied by rotating the movement of the cursor, so the greater the rotation the greater the deviation of the cursor according to the movement of the joystick (Figure 2) (for an example see: Angulo-Barroso, Ferrer-Uris, & Busquets, 2019; Ferrer-Uris, Busquets, & Angulo-Barroso, 2018; Miall et al., 2004).

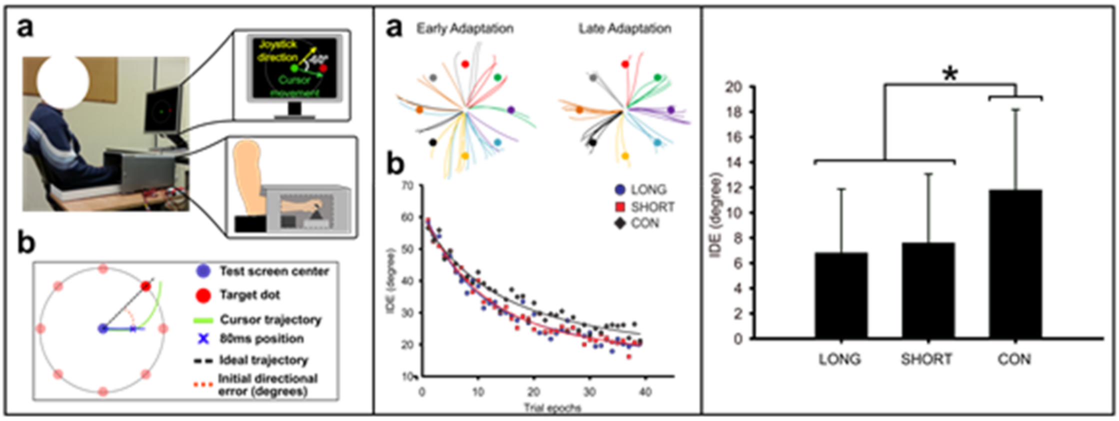

Figure 2: (Left) (a) Experimental setup of the rotational visuomotor adaptation task. Participants were seated in front of a computer screen and manipulated an on-screens cursor using a joystick. At certain point (adaptation and retention trials), the cursor movements were deviated 60 degrees from the joystick movements. (b) Example of a single trial analysis and computation of the initial directional error (IDE), a variable which reflects the error in the planning of the movement. (Middle) (a) Representation of the cursor trajectories and associated target (using a color code) at the start and at the end of the task practice during the adaptation trials of the motor skill. (b) Representation of the learning curve, error reduction, along the trials during the task adaptation. Learning curves of three groups are represented: one group performed a long exercise bout (LONG, 13-min), another group performed a short exercise bout (SHORT, 5-min), and the last group rested (CON) before the motor task's adaptation. No exercise effect was observed during the adaptation of the motor task. (Right) Mean retention performance (consolidation) of the three experimental groups. Both exercise groups presented better consolidation of the motor task (lower error values) compared to the CON group.

Children with DCD showed difficulties in motor adaptation (Bo & Lee, 2013). The internal model generation capacity in children with DCD has been studied using rVMA tasks (Kagerer, Bo, Contreras-Vidal, & Clark, 2004; Kagerer, Contreras-Vidal, Bo, & Clark, 2006; King, Kagerer, Harring, Contreras-Vidal, & Clark, 2011). Kagerer et al. (2004) exposed children with and without DCD to a novel visuomotor task. In the baseline condition (no rotation), DCD children were more variable and showed lower accuracy than TD children. When the visual feedback was rotated 45º in relation to their hand movements, children with DCD did not show observable after-effects compared to TD children. That is, DCD children did not realize that the alteration was removed indicating a weak modification of their internal model during the adaptation phase. The authors suggested that DCD children had a poorly defined visuomotor internal model in the baseline condition (i.e., larger variability) that made difficult to detect differences if they did not overpass this variability level. A follow up study conducted by Kagerer et al. (2006) indicated that both groups (DCD and TD) adapted to the visuomotor perturbation when it was suddenly introduced (i.e., 60º perturbation) but adaptation in DCD children was smaller than in TD with similar number of trials. Conversely, if the perturbation was gradually presented (i.e., increments of 10º of perturbation to achieve 60º), DCD children did not appear to be able to use the small differences in perceptive signals to adapt their internal model. These results seem to indicate that: (1) DCD children may be able to adapt their internal model but they will need enough sensory discrimination to generate error feedback (i.e, the alteration must be larger than variability); and (2) the rate learning of DCD children may be slower than TD children (Gomez & Sirigu, 2015) . Given the known role of the cerebellum in providing an error signal necessary for updating the sensorimotor mapping in response to a gradual visuomotor distortion, findings from Kagerer et al. (2006) implicated compromised cerebellar function in children with DCD (H. Imamizu, Kuroda, Miyauchi, Yoshioka, & Kawato, 2003; H. Imamizu, Kuroda, Yoshioka, & Kawato, 2004; Hiroshi Imamizu et al., 2000; Zwicker et al., 2010). Additionally, suboptimal parietal and cerebellar networks in DCD due to poor visual spatial integration for online movement controls and poor motor

preparation has also been suggested in children with DCD (H. Imamizu et al., 2003, 2004; Hiroshi Imamizu et al., 2000; Zwicker et al., 2010).

In addition to the motor learning (acquisition) problems observed in children with DCD (Jelsma, Ferguson, Smits-Engelsman, & Geuze, 2015), many studies defined performance problems as well. Such studies define children with DCD as being slower (Debrabant et al., 2013; Kagerer et al., 2004; Lejeune, Wansard, Geurten, & Meulemans, 2016), less accurate (Kagerer et al., 2004; Smits-Engelsman, Wilson, Westenberg, & Duysens, 2003), and having more variability (King, Kagerer, et al., 2011; Roche, Viswanathan, Clark, & Whitall, 2016; Smits-Engelsman & Wilson, 2013) in the performance of motor skills than their TD peers Therefore, it seems reasonable to examine performance variables, especially variability, in addition to learning outcomes.

Acute exercise and motor learning:

As mentioned before, exercise, and particularly, acute exercise could be an enhancer of function (Chuang et al., 2015; Lambrick et al., 2016; Piepmeier et al., 2015), including motor learning (Ferrer-Uris, 2017; Ferrer-Uris et al., 2018). However, evidence for an acute exercise-motor learning relationship does not exist for children with DCD. Furthermore, very few studies have examined this question in children with TD. To our knowledge, only one study, in addition to our own research, explored the effect of acute exercise on learning a tracking task in children with TD (Lundbye-Jensen, Skriver, Nielsen, & Roig, 2017). In this study, the consolidation of the tracking task was improved when participants performed a short bout exercise. In our own research we analyzed the effects of acute exercise on the acquisition and consolidation of an rVMA task in TD children. We observed how a 13 minute acute bout of exercise enhanced consolidation, especially when exercise was performed before practicing the motor task (Ferrer-Uris et al., 2018). Furthermore, we also observed how even a shorter exercise bout of only 5 minutes could enhance motor learning (consolidation) (Ferrer-Uris, 2017). Therefore, although information is scarce regarding acute exercise effects on children's motor learning, existing evidence points out that exercise, even when it is as short as 5 minutes, could potentially benefit learning in TD children. Since children with DCD have motor learning deficiencies, we set up this project to address whether they could benefit from an acute exercise intervention.

Despite that little is known about why exercise contributes to learning enhancements, mechanisms underlying this benefits have been related to changes in brain activity and concentration increases in neurochemicals (Taubert, Villringer, & Lehmann, 2015). Studies utilizing fNIRS have shown brain activation changes due to exercise in adult and elderly (Hyodo et al., 2012; Yanagisawa et al., 2010). Furthermore, it seems that acute exercise transiently affects adults' brain function through an increase in the concentration of certain neurotransmitters (e.g., catecholamines) and neurotrophic factors (e.g., brain derived neurotrophic factor, BDNF) (McMorris & Hale, 2012; Skriver et al., 2014; Taubert et al., 2015; Winter et al., 2007). Acute exercise-associated increases in BDNF and catecholamine secretion apparently enhance learning through facilitation of neuroplasticity-related processes (e.g., LTP) (Hötting & Röder, 2013; Kempermann et al., 2010; Taubert et al., 2015). Difficulties to measure central increases of these compounds in humans call for caution when interpreting these

results. Therefore, concurrent brain imaging and behavioral assessments would be the next step to explore the exercise benefits on learning in children. To our knowledge, there are no such studies, and certainly not in children with DCD.

Attention

Attention as a potential mechanism to explain deficits in motor learning in DCD

Attention is critical for skill acquisition, with mild or moderate impairments impacting learning efficacy (Boyd, Vidoni, Siengsukon, & Wessel, 2009). Three to four different types of attention have been defined in the literature: orienting, selective, divided and sustained (Fortenbaugh, DeGutis, & Esterman, 2017; Posner, 1990; Raz, 2004). Sustained attention is particularly important when learning has to occur in an invariant, relatively predictable context as in the learning of the rVMA task. A child does not need to divide, select or differently orient its attention when confronted with a computer screen and joystick to control a marker going to predetermined target positions, whether congruently or in a rotated fashion. On the contrary, sustain attention is critical since learning this task requires many practice trials which follow relatively fast. A small break in the maintenance of attention to the task will have large error consequences. In addition, limited capacity to sustain attention may hinder the formation of an internal model necessary to consolidate the task. Important attention related information can be obtained by examining multiples measures of behavior such as the reaction time which has been shown to be highly sensitive to changes in sustained attention (Derosière, Dalhoumi, Perrey, Dray, & Ward, 2014; Fortenbaugh et al., 2017).

A particularly sensitive period of development for sustained attention capacity seems to be between 6 to 9 years of age. Betts, Mckay, Maruff, and Anderson (2006) investigated sustained attention in children with TD between 5 and 12 years of age. They diagnosed a rapid development from 5-6 years to 8-9 years and a developmental plateau from 8-9 years to 11-12 years. Whether this developmental trajectory is the same for children with DCD is not known, but it places a target age range to examine issues of attentional development in children with DCD.

Children with DCD have more attentional problems than their peers (Dewey et al., 2002). Specifically, Fong et al. (2016) found that children with DCD were less attentive to M-ABC movements than their peers, even when subjects with comorbid ADHD were eliminated. These authors used a one-channel electro- encephalography (EEG) placed over the prefrontal cortex (PFC) to assess attention (Fong et al., 2015) showing that the attention index was significantly associated to M-ABC motor impairment so poor motor performance was explained by inattention after controlling for age, sex, body mass index and physical activity level.

It seems that different brain activation measurement techniques, such as EEG and functional magnetic resonance imaging (fMRI), could assist in elucidating group differences in attention. For instance, the dorsolateral prefrontal cortex (DLPFC) has been linked, together with anterior cingulate, to attentional control (Fassbender et al., 2004; Milham, Banich, Claus, & Cohen, 2003). Using fMRI and performance on an attention inhibition task, Querne et al. (2008) have suggested that children with DCD (without ADHD) may have a dysfunction in the attentional

brain network, as evidenced by less prefrontal activity compared to controls during a “go/no-go” task. Also, Zwicker et al. (2011) found lower levels of blood oxygen-level dependent (BOLD) signal in DLPFC in a trail tracking task (learning and retention). Similarly, Debrabant et al. (2013), using fMRI and a sequence learning task with predicted or unpredicted interstimulus intervals found that DCD children showed less activation than typically developing children in the right DLPFC. Together, these results suggest that poorer attentional capability may be one factor that impacts motor skill acquisition in children with DCD and neuroimaging can help elucidate these differences. However, whether these differences are also mediated by the visuomotor integration network in children with DCD has not been examined.

Although EEG and fMRI are techniques widely used, optical technologies provides a less invasive and more practical way to examine brain activation in pediatric populations (Buss, Fox, Boas, & Spencer, 2014; Imai et al., 2014; Lloyd-Fox, Blasi, & Elwell, 2010; Lloyd-Fox, Wu, Richards, Elwell, & Johnson, 2015; Nishiyori, Bisconti, Meehan, & Ulrich, 2016; Vanderwert & Nelson, 2014). Functional near infrared spectroscopy (fNIRS) technique uses light to measure changes in cerebral oxygenation and deoxygenation fractions of hemoglobin ([O_2Hb] and [HHb], respectively). It has been used in adults to reliably determine different levels of brain activity in different conditions, including activity of regions related to sustained attention (Derosière et al., 2014) (Figure 3). Similarly, fNIRS has been used in pediatric populations such as children with and without disabilities to examine attention and executive function (response inhibition, cognitive shifting, working memory, and attention) (for a review see, Moriguchi & Hiraki (2013). Reliability of the fNIRS as measures of brain activation in children has been proven (Blasi, Lloyd-Fox, Johnson, & Elwell, 2014). Recently, Caçola, Getchell, Srinivasan, Alexandrakis, & Liu (2018) used fNIRS to examine cortical activation in children with DCD performing different tasks (finger tapping, curve tracing, and paragraph writing). DCD children compared to TD children showed different focal activation patterns, even for the finger tapping (a simple task with no spatial or temporal demands), and these differences were also task-specific (for example, differences in the right DLPFC were found for the paragraph writing).

	Figure 3. *Near Infrared Spectroscopy (NIRS) pilot data during the execution of*

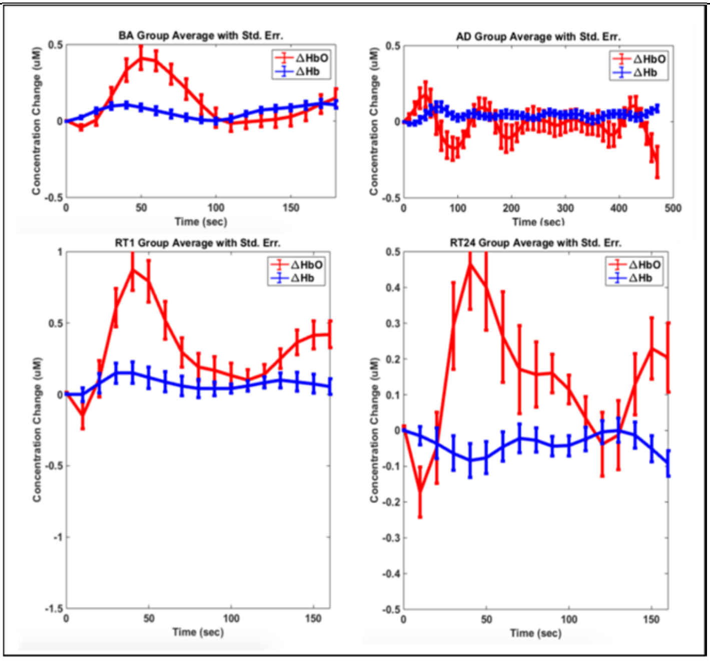

*the rotational Visuomotor Adaptation task (rVMA). These data are part of an own unpublished study, were 21 participants practiced the rVMA along 4 task sets: a baseline set (BA), an adaptation set (AD), a retention set 1 hour after AD (RT1), and a retention set 24 hours after AD (RT24). A single-channel NIRS probe was located over the dorsolateral prefrontal cortex. Oxy (HbO) and deoxy-hemoglobin (Hb) were registrered, filtered, and normalized (ΔHbO and ΔHb). Afterwards average ΔHbO and ΔHb were computed for analysis purposes. Data are presented as means and standard errors of the 21 participants for each task set. (Ramos-Moreno, **Busquets**, **Ferrer-Uris**, **Angulo-Barroso**, Eken, and Durduran, manuscript in preparation)*

In addition to fNIRS, a novel optical technology based on laser speckle statistics has been developed recently (T. Durduran, Choe, Baker, & Yodh, 2010; Turgut Durduran & Yodh, 2014) . Diffuse light also carries information about the motion of the scatterers, in this case dominated by the movement of red blood cells (i.e. blood flow) and this is exploited by the diffuse correlation spectroscopy (DCS) approach to measure deep tissue micro-vascular blood flow index (BFi). Functional fDCS is a relatively new technique, which has been utilized *extensively* in subjects of all ages – premature born infants to aging adults - and in animal models of all sizes. It has been validated against a multitude of modalities (T. Durduran et al., 2010; Turgut Durduran & Yodh, 2014) for the measurement of cerebral blood flow (CBF) and cerebral metabolic rate of oxygen extraction ($CMRO_2$). Despite CBF and $CMRO_2$ play a crucial role in neuro-vascular coupling in health and in diseases, its potential in neuro-sciences and clinics has been under-exploited because devices to measure them were not available. fDCS provides direct assessment of these relevant neurological variables allowing for more accurate assessment of distributed neuronal functional. This pioneering technique was first introduced by Durduran et al. (T. Durduran et al., 2010; T. Durduran, Kristoffersen, Varma, Valdes, & Culver, 2013; Turgut Durduran & Yodh, 2014; Valdes et al., 2014; Varma, Valdes, Kristoffersen, Culver, & Durduran, 2014) . In this project our group collaborates with ICFO Medical Optics group led by ICREA professor Turgut Durduran at ICFO (The Institute of Photonic Sciences). They are the leaders of fDCS in Spain and amongst the pioneering groups worldwide.

To our knowledge, no studies have utilized optical neuroimaging in children with DCD to explore brain responses in a motor learning context, much less the impact of acute exercise, and this project is a first attempt to provide some information. In addition, we propose to use the most advanced non-invasive technology (i.e., fNIRS and fDCS) to acquire insightful results.

Attention: Exercise as an enhancer of learning via attention

Although the specific underlying mechanisms by which physical exercise can improve learning and memory are not clear, several proposals have been placed forward. At the molecular level, and as mentioned before, changes in the concentrations of catecholamines (e.g. norepinephrine; T. McMorris, Collard, Corbett, Dicks, & Swain, 2008) and neurotrophic factors

(e.g. brain derived neurotrophic factor, BDNF; Koehl et al., 2008; Schwarz & Kindermann, 1990; Winter et al., 2007) have been shown to be related to the enhancement of learning when exercise is implemented. Alternatively, two psycho-cognitive factors, arousal and attention, may also mediate the relationship between physical exercise and improved learning. An optimal level of arousal is necessary to effectively use the limited attention capacity (Kahneman, 1973). This effective use of attention, in turn, facilitates learning (Catroppa & Anderson, 1999).

It is, therefore, plausible that moderate acute physical exercise places the individual at the appropriate level of arousal (not too high, not too low) and assists in the maintenance of attention, so the learning and consolidation of a new motor-perceptual task, like the rVMA task, are improved. In fact, Medina et al. (2010) found that the attention deficits in children with ADHD could be minimized via physical exercise using an acute interval exercise of 30 minutes and assessing sustained attention using the Conner's Continuous Performance II test. However, these authors had no measurements of learning or memory. Furthermore, no studies have examined the effect of acute exercise on the attention capacity of children with DCD and whether sustained attention plays a role in the exercise-induced enhancement of learning.

JUSTIFICATION AND HYPOTHESES

This project is designed to provide a multidisciplinary link between the educational sciences, sports sciences, and neurosciences to better understand a relevant population of children suffering from DCD. Results from this project have translational applications to teachers, parents, and other children educators that will allow them to elaborate adequate interventions from solid evidence (i.e., science-based interventions). In addition, innovative key-enabling technologies use will ensure that the knowledge generated will go after the next frontier in education and neuroscience field. The key concept is the emerging idea that physical exercise is the “new preventive medicine” for chronic vascular and metabolic diseases, but also it has been shown to be an enhancer of learning, even as a result of an acute bout of exercise. Children are at a developmental phase where the brain is still very plastic and establishment of enduring healthy habits is more effective. Children with DCD have problems with learning motor skills which are the support for a long-lasting active life style, and becoming physically active adult individuals. Unfortunately, therapeutic interventions are not main stream for this population despite its high prevalence (6%).

Motor learning entails integration of motor and sensory information and is constraint by our capacity to sustain attention. It is suggested that difficulties to perform motor tasks by children with DCD are related to poor visuomotor integration and attention deficits. Our previous work suggested that improvements learning a motor skill are related to experience in exercise and sport (Busquets, Marina, & Angulo-Barroso, 2013; Busquets, Marina, Irurtia, Ranz, & Angulo-Barroso, 2011; Ferrer-Uris et al., 2018) even when learners suffer some disorder or functional incapacity (R. M. Angulo-Barroso et al., 2017; R. M. R. Angulo-Barroso, Peirano, Algarin, Kaciroti, & Lozoff, 2013; Valentín-Gudiol et al., 2017). Furthermore, results from our studies (R. Angulo-Barroso et al., 2019; Ferrer-Uris et al., 2018; Ferrer-Uris, Busquets, Lopez-Alonso, Fernandez-del-Olmo, & Angulo-Barroso, 2017) have demonstrated that acute intense exercise enhances motor learning in adults and children with typical development. However, whether children with DCD could benefit from this strategy to improve their motor learning capacity is not known. The scarce brain studies conducted with adults about the exercise-learning interaction (Dal Maso, Desormeau, Boudrias, & Roig, 2018) indicate that acute exercise benefits on motor learning may be associated, in part, to changes in the brain activity. However, no similar studies in children, much less in DCD children, have been published thus far. Therefore, concurrent brain imaging and behavioral assessments would be the next logical step to explore the exercise benefits on learning in children with or without DCD.

In order to examine the combined effect of acute exercise and learning on children's brain activity, widely used neuroimaging techniques are either too expensive, or do not allow for in-situ assessment (i.e., same location where learning is occurring, in our case, children's school). Optical technologies have been utilized and suggested as potential and promising tools to fully study the alterations of the brain networks. Perhaps most importantly, the features of the new technological approach (non-invasive, quantitative, safe, inexpensive and portable) and the characterization of brain activity using fNIRS will allow us to conduct studies in-situ (i.e., children’s school) with robust results, including estimation of what brain areas are more or less activated while learning in children with DCD. In addition, combination of two optical techniques, fNIRS and DCS, has the potential to add new reliable information of the children’s brain activity during learning which open the door to conduct a pilot study to verify this potential. This knowledge is hypothesized to assist in the design of better, more targeted interventions. In addition, exercise benefits in learning, if confirmed in this population, could also be integrated as part of effective educational interventions for children with DCD. Our research hypotheses are arranged around these concepts.

- H1: Children with DCD will show worse motor learning (slower movements, more variability, slower rate of learning, and worse consolidation) than

typically develop (TD) children when facing a rotational visuomotor adaptation task (rVMA). Performing an acute intense exercise bout before rVMA will improve consolidation in TD and DCD children. In addition, exercise will narrow the gap between children with DCD and children with TD. Based on the null exercise effect on adaptation previously found in TD children, we have no predictions for DCD children.

- H2: Cortical activation measured by fNIRS from the visuomotor integration and the attentional network areas during the baseline phase will be lower in the children with DCD than in children with TD. Relative activation levels ([O_2Hb) in both networks during adaptation and consolidation will demonstrate opposite profiles in children with DCD (start low, end high) vs children with TD (start high and end low). In addition, acute intense exercise will narrow, but may not completely close, the gap between children with DCD who receive the exercise intervention and children with TD controls.
- H3: In a pilot study, combining fDCS technology that measures $CMRO_2$ and rCBF with fNIRS technology will provide us with the possibility to obtain more accurate and robust information of the children's brain activity during learning than using fNIRS alone.

OBJECTIVES

Although the long term objective of this line of research will address the underlying mechanisms of acute exercise effects on motor learning and possible brain activity changes with developmental coordination disorder (DCD), we currently defined the specific objectives of this project as follows:

- O1. To study the acute intense exercise effects on the motor learning (adaptation and consolidation) in children with and without DCD from a behavioral perspective (performance and execution).
  - O1.1- Characterize the possible motor learning modulators (physical fitness; physical activity level; attentional and cognitive capabilities) in children with and without DCD.
  - O1.2- Compare the motor learning (adaptation and consolidation) ability of children with and without DCD while performing a rotational visuo-motor task (rVMA) (CON-DCD vs. CON-TD).
  - O1.3- Examine the effect of acute intense exercise on motor learning (adaptation and consolidation) while performing a rotational visuo-motor task in children with and without DCD.
- O2. To explore cortical activation in the visuomotor integration and the attentional network areas while learning a motor adaptation task in children with and without DCD under rest or post-exercise conditions. Visuomotor integration and attentional network areas will include dorsolateral pre-frontal cortex (DLPFC), ventrolateral pre-frontal còrtex (VLPFC), and oculo pre-frontal còrtex (OPFC).
  - O2.1- Assess changes in cortical activation ([0_2Hb]) during rVMA learning (adaptation and consolidation) in children typically developed and in children with DCD.
  - O2.2- Evaluate the impact of acute intense exercise on changes in cortical activation ([0_2Hb]) during rVMA learning (adaptation and consolidation) in children typically developed and in children with DCD.
  - O2.3- Associate the presence of changes in the cortical activation variables with differences shown in behavioral variables.
- O3: To explore via a pilot study the combined use of two optical neuroimaging techniques (fNIRS and fDCS) to measure cortical activation in in the visuomotor integration and the attentional network areas while learning a motor adaptation task in children with and without DCD under rest or post-exercise conditions.
  - O3.1- Evaluate the usability of the hybrid device
  - O3.2- Assess the potential to obtain new relevant information of the hybrid device in comparison with using on technology (fNIRS or fDCS) alone.

METHOD

Participants

A total of 120 participants divided into two cohorts (60 children with DCD and 60 TD children) will be recruited for the study. Taken into consideration a 5-6% prevalence for DCD in the children population (Blank et al., 2012; Harris, Mickelson, & Zwicker, 2015; Kadejso & Gillberg, 1999; Lingam et al., 2009; Tsiotra et al., 2006; Zwicker et al., 2012), screening a total amount of 1000 children (from 6-8 schools, see Annex 2) using a motor-based questionnaire completed by parents and by teachers (M-ABC2 checklist, Henderson, Sugden, & Barnett, 2007) will be a first step to identify the necessary children with DCD on the basis of their difficulties in daily living activities and academic performance. Those children early identified as DCD by the motor-based questionnaire will be then evaluated to confirm their inclusion in the DCD group. Children in the DCD group will be selected based on the following inclusion criteria: (1) a movement assessment battery for children – second edition (MABC-2, Henderson et al., 2007)score of <15% administered by trained testers. The MABC-2 is a standardized and normative referenced test that is designed to identify motor impairment in children aged 3-16 years by evaluating manual dexterity, ball skills, and static and dynamic balance. Because DCD children present motor performance substantially below that expected given the person's chronological age and intelligence, a <15% cut-off is usually used to indicate children with probable DCD (Blank et al., 2012; Gueze et al., 2001). The Spanish translated edition and norm-referenced in Spain MABC-2 (Ruiz & Graupera-Sanz, 2012) will be used for scoring. (2) An average or better cognitive ability tested through the Test of Nonverbal Intelligence version 4 (TONI-4) and scoring above 85 in the intellectual quotient (IQ). (3) A parent-report history to confirm that motor difficulties showed by their child cannot be explained by any other neurological, developmental, and /or severe psychosocial problem according to the child's pediatrician. Comorbid attention deficit hyperactivity disorder, attention deficit disorder, and dyslexia will be acceptable in order to better represent the DCD population since data population-based studies suggest that almost 40% of the children with DCD have combined problems related to learning and/or attentional disorders (Blank et al., 2012; Harris et al., 2015).

The TD cohort will demonstrate usual levels given their age performing daily activities and academic performance, 25th percentile or greater score on M-ABC2 (Chia et al., 2014), an average cognitive ability (>85 IQ in the TONI-4), and a parent-reported history confirming that their child does not have any problem and/or disability affecting motor learning according to the child's pediatrician. The TD children cohort, and subsequent groups, will be matched to the DCD cohort and subgroups using age, gender, weight, handedness, physical activity engagement, and fitness level because these factors could affect the acute exercise effect on cognitive performance (Labelle et al., 2014) as well as the development of visuomotor representations (Contreras-Vidal, Bo, Boudreau, & Clark, 2005). Uncorrected 20/20 vision will be an exclusion criterion for all children.

Participants of both cohorts (DCD and TD) will be randomly assigned to one of two groups depending on the performance or not of an exercise bout prior to learning the task: (1) EX, who will engage in the learning task after exercising, and (2) CON, who will rest until the

learning task. Participants will not have prior experience with the proposed learning task (i.e., the rVMA). In addition, a subsample of 10 children from each group (CON-DCD, CON-TD, EX-DCD, and EX-TD) selected randomly will be part of a pilot study where hybrid optical neuroimaging device (fNIRS + fDCS) will measure the brain activity. The number of participants in this pilot study will be constrained by hybrid device development.

Statistical power analyses were performed for sample size estimation, based on IDE data from Kagerer et al. (2006) study (N=20) comparing TD to DCD (specific objectives 1 and 2) and from Ferrer-Uris et al. (2018) study (N=21) comparing EX vs CON (specific objectives 3, 4, and 5). The effect sizes (ES) in these studies were 0.78 and 0.55, considered to be large from 0.5 to 0.8 using Cohen's (Cohen, 1988) criteria. With an alpha = .05 and power = 0.80, the projected sample size needed to achieve these effect sizes (GPower 3.1) is approximately between N = 12-26 per group for this simplest between group comparison (TD vs. DCD). Thus, our proposed sample size of 120 children (60 TD and 60 DCD; 30 EX and 30 CON within each cohort) should be adequate for the main objectives of this study and should also allow for expected attrition and our additional objectives of controlling for possible moderating factors, subgroup analysis, etc. Controlling for covariates will reduce residual error and further increase power.

Children's parents or legal guardians will be fully informed of all the details of the study. They will sign a consent form prior to their child's participation (Annex 3) as well as children participants will sign an assent form (Annex 4). All the experimental procedures will be carried out following the health safety rules established by our institution (INEFC) and by children schools. Treatment of the data will follow the current normative. A data management delegate from the Presidency of the Generalitat of Catalonia will be requested and a data management plan will be elaborated using DMPonline platform (https://dmp.csuc.cat/) to ensure data protection and their proper use. The study was submitted to the Ethic Committee of Clinical Research of the Catalan Sport Administration for its approval.

Experimental protocol

Participants will engage in five different sessions (see Figure 3). Before enrolling in any of the study sessions, participants along with their guardians will complete a set of questionnaires to assess (Annex 5): (1) the participant health status to participate in physical activity (Physical Activity Readiness Questionnaire, PAR-Q); (2) the participant's physical-activity engagement (Spanish version of the Physical Activity Questionnaire for Children, PAQ-C, validated by Benítez-Porres et al., 2016); (3) the participant's academic performance and difficulties performing daily living activities (Spanish version of the M-ABC2 checklist, validated by Ruiz & Graupera-Sanz, 2012); (4) the possible comorbidities permitted in this study such as attention deficit hyperactivity disorder, attention deficit disorder, and/or dyslexia; (5) the type and dose of medication a child currently takes; (6) possible psychosocial problems assessed by the Conners Comprehensive Behavior Rating Scales (Conners CBRS; Conners, 2008); (7) the exclusion criteria via a checklist; and (8) the family history about DCD, neurological disorders or mental health, and family's socio-economic status (SES) and parental education. At the same time, the participant's teacher will also complete the M-ABC2 checklist (Ruiz & Graupera-Sanz, 2012) questionnaire to identify motor coordination problems and the participant's academic

level. Participant handedness will be determined based on their preferred hand for everyday activities and confirmed by the M-ABC criteria.

In the first session, participants' intelligence level will be assessed through the TONI-4 while their motor development will be evaluated by the MABC-2. Basic anthropometric parameters (height and body mass) will be also obtained and used to calculate body mass index (BMI). The fitness level (estimated VO_2max) will be assessed through the 20-meter shuttle run test (20mSRT) (Léger, Mercier, Gadoury, & Lambert, 1988). The 20mSRT start at one longitudinal end of the zone limited by 20 m long and 1.5 m wide, and move back and forth following the rhythm of an audio track. Participants must match the arrival at each end of the bounded zone (stepping the line) with an auditory signal (beep). The initial speed is 8.5 km/h but the frequency of the beep increase every minute (stage) leading the participants to increase their speed 0.5 Km/h. The test ends when the subjects abandon or fail to maintain the rhythm for 2 consecutive lines. To encourage participants' performance during the fitness test and the intense exercise (iE) bout, for each 20 m back and forth ran they will receive card containing cartoon-character pictures that would be used in the third session of the study to perform a memory game of pairing two equal cards, so the more cards collected the better. To further support children, an adult will run next to the child to assist in keeping the pace and provide encouragement. The last completed stage (Emax) of the test will be recorded. In addition, during the 20mSRT, beat-by-beat values for the RR intervals (time between peak values in an electrocardiogram) will be registered using a Polar RS800CX (Polar Electro) at 1000 Hz frequency. The heart rate (HR) will serve as a control parameter for the intensity of the test. A minimum of 48h delay will be applied between sessions 1 and 2. Only participants that meet inclusion-exclusion criteria will be enrolled further into the study.

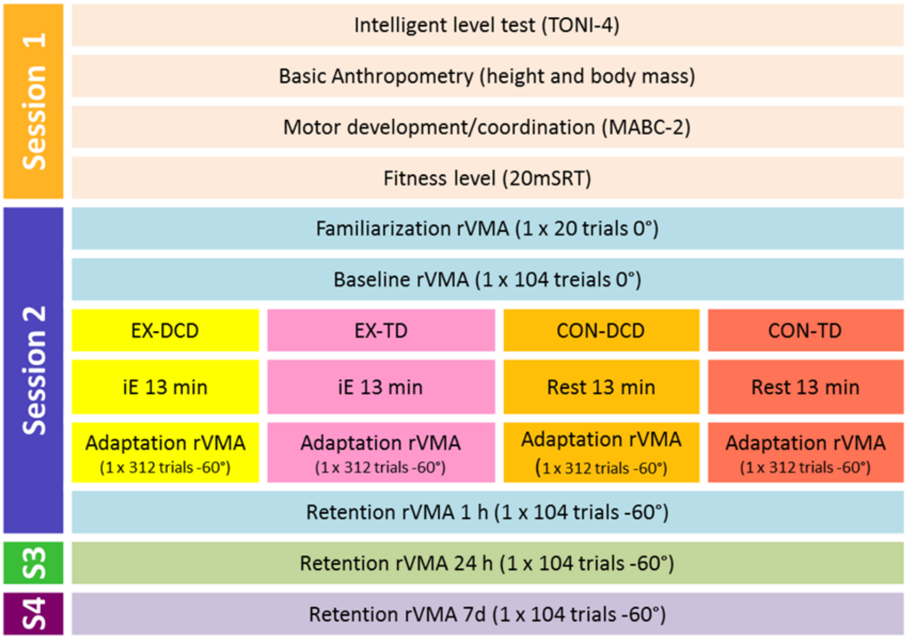

Figure 4. Schematic overview of the experimental procedure.

At the start of session 2, participants will be familiarized with the rVMA protocol (familiarization set) by performing 20 trials of non-rotated (0°) practice trials. Afterwards, a non-rotated baseline condition set (104 trials) will be performed. After the baseline set, the EX groups (i.e., EX-DCD and EX-TD) will perform a 13-min iE session while the CON groups (i.e., CON-DCD and CON-TD) will rest the same period of time reading or holding a conversation, no additional exercise or musical activity will be permitted. Next, all participants will do an adaptation set (312 trials) in the rVMA task, with a clockwise rotation of 60° applied to the cardinal coordinates of the cursor movement. At one hour after the end of the adaptation set, all participants will perform a 60° clockwise retention set (short-term retention, RT1h, 104 trials). At the end of session 2, all participants will perform a memory game with the cards they collected during the 20mSRT and potentially the iE.

Sessions 3 and 4 will occur 24 h and 7 days from the end of the adaptation set. In each session, participants will perform a 60° clockwise rotated retention set (mid and long term retentions, RT24h, and RT7d respectively, 104 trials each).

The rotational visuomotor adaptation task (rVMA)

During the rVMA participants will be seated in a quiet room, in front of a 19-inch computer screen located at 1m and at eye level. With their dominant hand, participants will be asked to grasp a joystick, maintaining a claw-type grip, an elbow flexion of 90° and a comfortable shoulder position while the forearm rests on a flat surface (Figure 2a). The height and position of the joystick will be adjusted to meet the position criteria. The joystick movement controls a green dot on the screen (1x1cm). Individual targets will randomly appear on screen as red dots (1x1cm) in eight possible locations (45, 90, 135, 180, 225, 270, 315 and 360°) and at a radius distance of 13 cm from the center. A new target will appear every 2 s and will remain visible for 750 ms. Participants will be instructed to move the green dot, starting from the center of the screen, over the target (red dot) and back to the center as fast and as straight as possible in a single move. During the task a visual-motor mismatch between joystick movement and screen cursor movement is applied by rotating the movement of the cursor, so the greater the rotation the greater the deviation of the cursor according to the movement of the joystick (for example see Ferrer-Uris et al., 2018).Cartesian x-y coordinates of the joystick movement and time will be registered at 120Hz through a NI-6008 card (National Instruments Corporation).

The intense exercise bout (iE)

The intense exercise (iE) bout will consist of a 13-minute 20-meter shuttle run. During this exercise bout two speeds, based on a percentage of the estimated VO_2max, will be combined: a fast-paced speed (fast, 85% of VO_2max) and a slow-paced speed (slow, 60% VO_2max). A total of 3 series of 3 min of the fast-paced speed will be carried interspersed with 2 series of 2 min of the slow-paced speed. Prior to the iE start, a warm-up protocol consisting of 2 min slow and 1 min fast will be done with the objective to familiarize participants with the iE speeds. A 5-minute rest period will be guaranteed before starting the iE. Transition time between iE and rVMA will be 4 min. Participants' hearth rate will be captured following the same procedure described for the 20mSRT.

Optical neuroimaging: The functional Near-Infrared Spectroscopy (fNIRS) and the functional Diffuse Correlation Spectroscopy (fDCS) protocol

During sessions 2, 3, and 4, 27-channel NIRS instrument (Brite MKII, Artinis Medical Systems, Netherlands) will be placed in the head of the participants to perform recordings of the hemodynamic changes of their right and left dorsolateral pre-frontal cortex (DLPFC), ventrolateral pre-frontal còrtex (VLPFC), and oculo pre-frontal còrtex (OPFC) to cover motor, perceptual and attentional areas. Placement of the optodes and cap will be anchored to various landmarks in the 10-20 EEG electrode placement system (Cao et al., 2015) . Participants will be asked to rest seated during 30 seconds before the familiarization, baseline, and adaptation sets (session 2), the RT24h (session 3) and the RT7d (session 4) to record a reference resting state in the fNIRS signals before to start the rVMA task. In the pilot study to explore the usability of a fNIRS and fDCS hybrid device, DCS sensors will be placed closer to fNIRS sensors to perform simultaneous recordings of the areas defined previously. Both devices will be placed at the same time and their recording will be synchronized.

The Brite MKII is a portable wave continuous system developed to measure concentration changes of oxyhemoglobin ([0_2Hb]) and deoxyhemoglobin ([HHb]), which indirectly reflect brain activity. The light sources (optodes) transmit two wavelengths, ±760 nm and ±850 nm, and the receiver is a high sensitive PIN diode with ambient light protection. The distance between the receiver and the optodes and the sensitive PIN diode and the intensity of the light can be modified to better adjust to participant characteristics. Spring-loaded grommets will be used to improve data quality in dark thick hair. Data acquisition sampling rate will be set to 10 Hz. Oxysoft software (v6.0, Artinis Medical Systems, Netherlands) will allow real time assessment of the quality of the fNIRS signals and zero baseline will be set when acceptable signal-to-noise ratio was obtained.In the pilot study, fDCS will measure cerebral metabolic rate of oxygen extraction ($CMRO_2$), and microvascular cerebral blood flow (CBF) during all motor task phases to assess short and long-term changes in cortical processing. Data acquisition sampling rate of the hybrid device (fNIRS and fDCS) will be set at the same frequency when they collect data together. fNIRS and fDCS data will be analyzed using programs wrote by our team and our collaborators in ICFO.

Data reduction

Data of the 20mSRT will be used to characterize participants' fitness level and to define the intensity of the exercise during iE performance in the second session. Individual maximum velocity (Vmax, km/h) will be calculated using the last completed stage (Emax) (Léger et al., 1988).

$$V_{max} = 0.5 \cdot \ E_{max} + 8$$

VO_2max will be estimated based on the participant Vmax and age (*y*, years) (Léger et al., 1988):

$$VO_2max = 31.025 + (3.238 \cdot V_{max}) - (3.248 \cdot y) + (0.1536 \cdot y \cdot V_{max}))$$

Individual heart rate (HR) will be monitored by a Polar RS800CX (Polar Electro) and it will be synchronized with the time of the 20mSRT test (session 1) and with the iE intervals (session 3). Maximal HR (HRmax) during the childhood and adolescence periods is typically above 200 b/min. Therefore, we will use this value as a reference to assess maximal effort level in the last part of the 20mSRT (Armstrong & Fawkner, 2007). In addition, the mean HR during the iE intervals (85% and 60% of the estimated VO_2max) will be calculated (HR-85%VO_2max and HR-65%VO_2max). These values will be used to further characterize the exercise's intensity during the iE.

The rVMA data fitting and reduction will be done using custom-made MATLAB R2014b programs (The MathWorks, Inc.). Cartesian x-y positions will be low-pass filtered using an eight-order dual-pass Butterworth filter with a cut-off frequency of 12Hz. Accepted trials will have to fulfill the following conditions: startup position found within 20% of the center-to-target distance, and travelled distance equal or higher to 90% of the center-to-target distance. Movement onset of the accepted trials will be defined as the nearest point in an outward movement equal to a 10% of the center-to-target distance. Movement offset will be defined as the first point where speed decreased to a 10% of the max speed value. The adaptation set will be divided in epochs of 8 trials for analysis purposes.

fNIRS and fDCS data, will be converted into changes in concentration levels of oxyhemoglobin ([0_2Hb]), deoxyhemoglobin ([HHb]), cerebral metabolic rate of oxygen extraction ($CMRO_2$), and microvascular cerebral blood flow (CBF). A subject-specific differential path-length factor (DPF) will be used for this conversion based on the age of the participant (Duncan et al., 1996; Scholkmann & Wolf, 2013). Data will be low-pass filtered to avoid excessive frequencies that will belong to normal physiology activity and noise from respiration and heart beats or movements (typically a cut-off frequency of 0.1 Hz). Filtered data will be normalized relative to the individual values found in the reference resting state measures. All these processes previous to calculate the variables will be computed using custom made programs.

Variables

The movement during the rVMA task will be described by the calculated movement time (MT, ms), travel distance (TD, cm) and reaction time (RT, ms) variables. RT will be defined as the time between target appearance and movement onset. Movement output error will be measured through the initial directional error (IDE, deg) and root mean square error (RMSE, cm) variables (Figure 5). IDE will be calculated as the absolute angular difference between the ideal trajectory, a linear vector from the center to the target, and the early real trajectory, defined by the linear vector from the center to the green dot position at the time of 80 ms after movement onset. IDE will be used as a measure of the rotation adaptation or motor consolidation avoiding the possible trajectory correction through perceptual feedback (Contreras-Vidal et al., 2005; Ferrer-Uris et al., 2018, 2017). RMSE will be calculated to represent the straightness of the movement between the ideal trajectory and the real joystick trajectory following the procedure described in Contreras-Vidal et al. (2005):

$$RMSE = \sqrt{\sum_{i=1}^{N}[(x_1 - x_2)^2 + (y_1 - y_2)^2]\frac{1}{N}}$$

where (x_1, y_1) and (x_2, y_2) are the coordinates of the real and ideal trajectory, respectively, and N is the number of points in the path. Movement straightness (i.e., RMSE) includes the initial deviation from the ideal trajectory (IDE) and the remaining movement trajectory, which could be corrected using perceptual feedback. All these variables will be calculated for each set (baseline, adaptation, RT1h, RT24h, and RT7d). Considering the large inter-subject variability usually presented by children, computed mean adaptation and retention variables will be normalized by subtracting the participant's mean baseline values.

It is proposed that DCD groups will show more variability than their TD peers (King, Harring, Oliveira, & Clark, 2011; Roche et al., 2016; Smits-Engelsman & Wilson, 2013). Inter-trial variability will be calculated via SD for all performance variables in the rVMA (MT, MT-SD, TD, TD-SDRT, RT-SD, IDE, IDE-SD, RMSE, and RMSE-SD) to characterize the groups.

During the adaptation set, data typically present an initial rapid-error decay followed by a slower decline. As it has been seen in other studies (Ferrer-Uris et al., 2018, 2017; Krakauer, Ghez, & Ghilardi, 2005), these data from adaptations seem to be best fitted by a double exponential function:

$$y = a * e^{b*x} - c * e^{d*x}$$

where y is the error (epoch's mean), x is the epoch number and a, b, c and d are parameters. The initial rate of learning (RL) will be computed, as described in Coats (2014), as the first derivative of the first half of the function and evaluated at epoch 1 for both error variables IDE (RL-IDE) and RMSE (RL-RMSE). All individual exponential functions will be visually inspected for a plateau to assess that learning is achieved.

Progress towards increasingly skilled performance consists of the acquisition and stabilization of more effective movement patterns (Vereijken, Van Emmerik, Bongaardt, Beek, & Newell, 1997). Inter-trial variability could be used to assess the stabilization level of the task. High values of variability are related to an early learning stage that explores new solutions for the task while low values of variability are associated with more skilled performers showing stable executions. Changes in variability across epochs in the acquisition set (i.e., rate of variability, RV) for both error variables (RV-IDE-SD and RV-RMSE-SD) will be also computed.

Normalized changes in concentration levels of hemodynamic variables during the different sets will be analyzed. Neural activation of each cortical area will be expressed as an increase of [0_2Hb] (also of $CMRO_2$ and CBF in the pilot study) and a decrease in [HHb] (Mandrick et al., 2013). Qualitative analysis of the changes in these variables across trials will be performed to establish differences in hemodynamic patterns. Average and SD of the hemodynamic variables for each set will be calculated.

Statistical analysis

Age, gender, weight, handedness, physical activity engagement, and fitness level (estimated VO_2max) measures were explored through one-way analysis of variance (ANOVA) to evaluate group differences. Non-parametric ANOVA will be used when necessary.

Descriptive statistics of all the scores /results obtained from the questionnaires answered by the parents and teachers, the anthropometric measures (height, weight, and BMI), and the tests conducted in sessions 1 (20mSRT) and 2 (TONI-2 and M-ABC2) will be used to characterize DCD and TD children (**O1.1**). Also, independent t-test will be applied to contrast the differences between the DCD and the TD cohorts (**O1.1**). Variables obtained from the rVMA will be used to elucidate and compare the motor learning ability of children with and without DCD while performing rVMA (**O1.2**) and the effect of acute exercise on this motor learning (**O1.3**), while data from the fNIRS will explore patterns of brain activation while learning a motor adaptation task in children with and without DCD and the effect of iE (**O2**). Data from fNIRS and dDCS collected during the pilot study will be used to assess the fDCS usability and point out the additional relevant information to use both optical technologies simultaneously (**O3**).

We will compare the mean baseline value for each rVMA and brain hemodynamics variables with ANOVAs to evaluate baseline performance across the four groups. Differences between the CON-DCD and CON-TD children groups (O1.2 and O2.1) on the motor learning (adaptation and retention phases) will be explored using independent t-tests in the adaptation set, while 2 (Group) x 3 (Time) ANOVAs with repeated measures will be used to analyze differences in the retention phases. Focusing on the effects of exercise (O1.3 and O2.2), possible differences in the adaptation set will be contrasted by 2 (Group) x 2 (Exercise) ANOVAs and retention tests will be analyzed by 2 (Group) x 2 (Exercise) x 3 (Time) ANOVAs with repeated measures. Association of the changes in brain activation variables with changes occurred in the rVMA variables will be explored using bivariate correlations and multivariate correlations (O2.3). Usability and additional information provided by fNIRS and fDCS will be evaluated qualitatively and contrasted brain activity levels obtained from both technologies using t-test and ANOVAs (adaptation: t-test (Technology); retention tests: 2 (Technology) x 3 (Time) ANOVAs) (O3).

Before any statistical test normality distribution will be tested for all variables via exploration of histograms, Q-Q plots and with the Shapiro-Wilk's normality test. When the normality assumption failed, variable transformation or non-parametric alternative tests will be adequately conducted. In all ANOVAs Greenhouse-Geisser sphericity-corrected values will be used when appropriate. Variables with initial group differences and significantly correlated with the dependent variable will be considered as potential covariates in the ANOVA analyses. Bonferroni *post hoc* analyses will be performed if significant differences are found. In addition, the effect size will be calculated using η^2p (0.01 small, 0.06 medium, 0.14 large effect). Statistical significance was set at $p<0.05$ for all analyses.

Ethic treatment

All the procedures of the present research project will be conducted according to the latest revision of the Helsinki Declaration. In addition, this research project has been sent to the Ethics Committee of the Consell Català de l'Esport for its approval.

Previous research has examined the effect of exercise on human cognition and how individual characteristics as fitness level, age, and gender could moderate the exercise effects on cognition. V. Labelle et al. (2014) found that exercise effects on cognition were not moderated by gender. Studies where exercise has been observed to stimulate motor learning in children included male and female in the groups (Lundby et al., 2017; Ferrer-Uris et al., 2018). Therefore, during the execution of this research no distinction for sex or ethnic characteristics will be made during participant recruitment. Yet, a greater number of male participants is expected because DCD has shown greater prevalence in male than female children. More male DCD probable cases are expected in the present study, compared to female DCD cases. Therefore, gender balance across groups may or may not be accomplished in this project. Despite no gender differences were expected, we will introduce sex as covariate in the statistical procedures to better know if any sex effect occurred. Dissemination and transfer of the research will be take care to high standards preserving the gender dimension explaining similarities and differences if any. To do so we will be assisted by our colleague in INEFC Dr. Susanna Soler and her group of research (https://inefcgiseafe.com/) who are highly recognized by their research and work about the gender dimension. All participants recruited during the study will provide verbal and written assent (see Annex 4) along with their guardian's written consent (see Annex 3). As stated in the assent and consent formularies, participants will be free to quit the study in any moment if they don't want to continue participating.

Treatment of the data will follow the current normative. Department of the Presidency of the Generalitat of Catalonia data management delegate (https://presidencia.gencat.cat/ca/el_departament/proteccio-dades/delegat-da-de-proteccio-de-dades/) will be request and a data management plan will be elaborate using DMPonline platform (https://dmp.csuc.cat/) to ensure data protection and their proper use. Participants' personal information will always be kept safe in order to preserve their anonymity. That is, participants' identity information will only be stored in a unique safe file and afterwards participants identity will be codified though all data registration and treatment procedures.

Lastly, a supervisor will be designated in each school in order to oversee that all study procedures are performed according to this ethics declaration. In addition, the school supervisor will be requested to provide support to the researchers in case of accident or injury of the participants during the experimental procedure, providing assistance with the emergency protocols of the school. The school supervisor will also be the mediator between researchers and participants/participants' guardians in any case of necessity.

WORK PLAN AND SCIENTIFIC ROLL OF EACH COLLABORATOR

The work plan involved: Dr. Albert Busquets, principal investigator 1 (PI1); Dr. Rosa Angulo, principal investigator 2 (PI2); Dr. Blai Ferrer, research team (RT1); Dr. Priscila Caçola, work team (WT1); Dr. Nadja Schott, work team (WT2); Neus Camins, work team (WT3); Faruk Beslija, work team (WT4). Below we present briefly the four researchers that form our work team:

- Dr. Priscila Caçola (Department of Kinesiology and the Director of the Developmental Motor Cognition Lab at the University of Texas at Arlington, USA) has two main lines of research: Low motor ability (Developmental Coordination Disorder) and Motor skill interventions. Her research focuses on the links between cognitive and motor behavior across the lifespan, with particular emphasis on how motor and space representations develop and how problems associated with those representations cause motor skill difficulties in vulnerable populations (e.g., children with low motor ability, DCD).
- Dr. Nadja Schott (Institute of Sport and Exercise Science in the department of motor and cognition at the University of Stuttgart, Germany) deals with central aspects of motor and cognitive performance over the entire lifespan. She investigates the impact of exercise programs on school performance and track down motor ineptitudes to promote motor and cognitive development. She has published in DCD since 2007, and she has also examined effects of acute exercise on inhibitory control.
- Neus Camins (INEFC, Barcelona, Spain) is a PhD candidate on her first year and she is studying the effects of the physical fitness in cognition in young adults (university students) with a multidisciplinary approach (neuroimage, psychological test, exercise test, microbiota). Also, she is collaborating with the Departament de Psicologia Clínica i Psicobiologia, Facultat de Psicologia, Universitat de Barcelona in a project to improve cognition and neuroplasticity in stroke patients through physical exercise intervention.
- Faruk Bešlija (ICFO, Castelldefels/Barcelona, Spain) is a first-year PhD candidate in the Medical Optics group at ICFO. He is currently involved in a research related with diffusive optics techniques for tissue imaging, with focus on applications in neurology. It is expected that he will soon be included in several collaborative projects with partners from Barcelona region, as well as Europe and United States, evolving around emerging optical methods for non-invasive and deep-tissue blood flow measuring and monitoring.

A list of the principal tasks related to the research objectives are presented indicating members of the group involved. Critical tasks that could alter the expected timing (underlined) are related to the accessibility of the sample and DCD children detection.

- **Task 1.** Participant recruitment
  - Task1.1. <u>Participant recruitment</u>. Investigators involved: PI1, WT3, WT4. Duration: M1-36.
- **Task 2.** Objective 1
  - Task 2.1. Collection of participant's characterization data, including: Health status (PARQ), physical activity engagement (PAQ-C), academic and daily living activities performance (MABC-2 checklist), possible comorbidities report, regular medication intakes, possible psychosocial problems (Conners CBRS), exclusion criteria related-data, and the family-related information. Investigators involved: PI1, PI2, WT3. Duration: M2-40.

- Task 2.2. Intelligence quotient data collection using the Test of on-verbal intelligence version 4 (TONI-4). Investigators involved: PI1, PI2, WT3. Duration: M2-40.
- Task 2.3. Motor coordination data collection using the Movement assessment battery for children – second edition (MABC-2). Investigators involved: PI1, PI2, WT3. Duration: M2-40.
- Task 2.4. Participant's anthropometric data collection. Investigators involved: PI1, PI2, WT3. Duration: M2-40.
- Task 2.5. Estimation of participant's VO_2max via the 20-meter shuttle run test (20mSRT). Investigators involved: PI1, PI2, WT3. Duration: M2-40.
- Task 2.6 Exercise intervention execution and motor learning (rotational visuomotor adaptation task) behavioral data collection. Investigators involved: PI1, PI2, RT1, WT3, WT4. Duration: M2-40.
- Task 2.7. Reduce data and carry bio-statistical procedures. Investigators involved: PI1, PI2, RT1, WT1, WT2, WT3. Duration: M7-45.
- Task 2.8. Produce results and disseminate according to dissemination plan. Investigators involved: PI1, PI2, RT1, WT1, WT2, WT3. Duration: M14-48.

- **Task 3.** Objective 2 and 3
  - Task 3.1. Brain activity data acquisition under resting state and baseline execution of the motor task (rotational visuomotor adaptation task without deviation of hand movements). Investigators involved: PI1, RT1, WT3, WT4. Duration: M2-40.
  - Task 3.2. Brain activity data collection during the acquisition and retention of the new motor skill (rotational visuomotor adaptation task with hand movements deviated 60 degrees clockwise). Investigators involved: PI1, PI2, RT1, WT3, WT4. Duration: M2-40.
  - Task 3.3. Reduce data and carry bio-statistical procedures. Investigators involved: PI1, PI2, RT1, WT1, WT2, WT4. Duration: M7-45.
  - Task 3.4. Produce results and disseminate according to dissemination plan. Investigators involved: PI1, PI2, RT1, WT1, WT2, WT3, WT4. Duration: M14-48.
- **Task 4.** Return to society and preparation of future projects
  - Task 4.1. Development of DCD awareness booklets/brochures and website. Investigators involved: PI1, PI2, RT1, WT1, WT2, WT3. Duration: M1-4 (phase 1) and M42-48 (phase 2).
  - Task 4.2. Workshops to increase knowledge about DCD between parents and educators. Investigators involved: PI1, RT1, WT1, WT3. Duration: M1-4 (phase 1) and M42-48 (phase 2).
  - Task 4.3. Define methodological procedures and prepare documentation for future projects. Investigators involved: PI1, PI2, RT1, WT1, WT2, WT3, WT4. Duration: M43-48.

Given the large sample needed for this project and the total number of sessions that each participant will be engaged in, we estimate that data collection would last 39 months approximately (school vacations periods included). Therefore, we elaborated a work plan schedule of 4 years (Figure 4).

Figure 4. Deliverables and chronogram

Deliverables:

D1, M2: Report on protocols.

D2, M12: Preliminary test of optical systems.

D3, M12: Project status report.

D4, M24: Update on data acquisition and recruitment.

D5, M24: Project status report.

D6, M36: Subject recruitment status and summary statistics.

D7, M36: Project status report.

D8, M40: Update on completed tasks.

D9, M48: Report on outreach and future planning.

D10, M48: Final project report.

Note: Deliverables are for internal management purposes and for evaluating theongoing research timing.

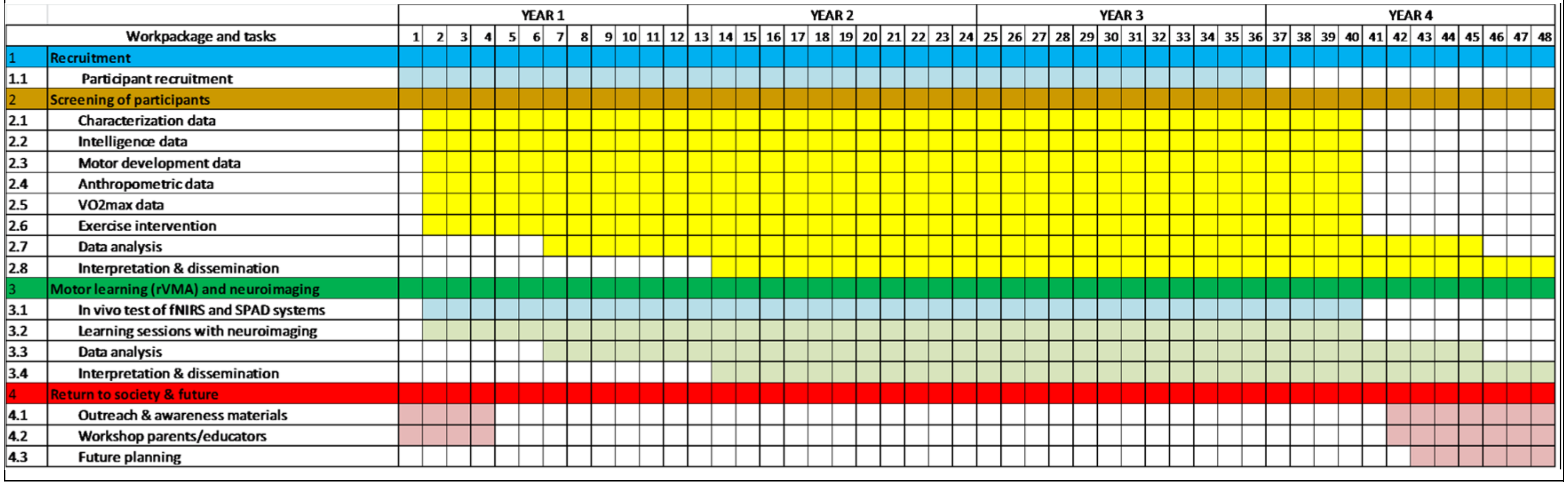

SCIENTIFIC AND SOCIAL INTEREST

Scientific interest

Children with DCD are characterized by a significant delay in the acquisition of gross and fine motor skills and impairment in the execution of coordinated motor skills (Who 2018). These motor coordination problems have been associated to other characteristics like lower fitness level (Cairney et al., 2011, 2017), physical activity engagement (Batey et al., 2014; Cermak et al., 2015), cognitive and academic performance (Dewey et al., 2002; P. H. Wilson, Smits-Engelsman, Caeyenberghs, & Steenbergen, 2017) and poorer socio-emotional status (Zwicker et al., 2013). However, little is known about how these characteristics interact and whether they can be used to predict possible cases of DCD. Children with DCD have also been observed to have difficulties with motor learning and not only motor control, presenting slower adaptations and worst consolidation in comparison to TD children (Jarus et al., 2015; Kagerer et al., 2004, 2006). However, the existing evidence is scarce and inconclusive, especially given that consolidation of motor memory has been weakly examined (Smits-Engelsman et al., 2015). They have also been described as less attentive (Dewey et al., 2002; Fong et al., 2016; Gomez & Sirigu, 2015; P. H. Wilson et al., 2017), a fact that could be strongly related to their learning impairments, although little is known about this fact (Debrabant et al., 2013; Zwicker et al., 2009). In summary, these children present various issues that could affect their health and quality of life, a fact that probably will endure into adulthood. In fact, children with DCD do not simply outgrow their motor impairments. Without intervention, it is estimated that nearly 75% of children with DCD continue to have difficulties as adults (Kirby, Sugden, & Purcell, 2014). Therefore, early identification of DCD and intervention of this disorder seems crucial. Acute physical exercise has been proven to improve motor learning in children (Ferrer-Uris et al., 2018; Lundbye-Jensen et al., 2017). Furthermore, acute exercise has also shown improvements in attention in children with ADHD (Chang, Liu, Yu, & Lee, 2012; Medina et al., 2010). Then, acute physical exercise could be proposed to improve learning and attention in children with DCD. However, there is no existing evidence of the effects of acute exercise on this population regarding motor learning and attention. Moreover, although that exercise effects have been related to increases in some neurochemicals (catecholamines and BDNF) (McMorris et al., 2008; Skriver et al., 2014; Winter et al., 2007) little is known about the possible mechanisms underlying exercise benefits on motor learning.

The present research project will add new evidence regarding the characteristics of DCD children through the proposal of a predictive model for this disorder, involving physiological, motor, cognitive and socio-emotional parameters. This predictive model could broaden our knowledge of these children, especially in the Catalan and Spanish populations were DCD children are not well characterized (Plata & Guerra, 2009). Aside from the participants' characteristics, the assessment of learning of a perceptual-motor task will add new evidence regarding how DCD children learn a new motor skill and how their learning is impaired in comparison to TD children. Because both processes during learning of a new motor skill, adaptation and consolidation, will be analyzed, this project will add new evidence on how children with DCD adapt visuo-motor integration tasks, how they consolidate motor memory

and how they retrieve the learned skill. Moreover, because attention will be assessed previous and during the learning of the new motor skill, new evidence will be available regarding the relation between DCD lack of attention and their motor learning impairments. Lastly, we will assess the acute exercise effects on attention and the learning of the presented motor task. This project will present prime evidence regarding the effect of acute exercise on the learning ability of children with DCD. Moreover, attention measures will help to define the possible contributions of exercise effects on motor learning, possibly through an increase in the capacity to maintain attention levels though the task practice. In addition, this project will promote the use of a groundbreaking optical device to neuroimaging learning in children.

Innovative approach of the project

Given the available research evidence in the literature (Piepmeier et al., 2015; Chuang, Tsai, Chang, Huang, & Hung, 2015; Lambrick, Stoner, Grigg, & Faulkner, 2016) it seems reasonable to think that acute exercise may also benefit children with DCD but no studies, to our knowledge, have examined this question. Additionally, little research is available that focused on how children with DCD learn new motor skills and the evidence is inconclusive given that consolidation and transfer of the learning has been weakly examined (Smits-Engelsman, Jelsma, Ferguson, & Geuze, 2015). Furthermore, studies where concurrent brain imaging and behavioral assessments are used would be the next step to explore the exercise benefits on learning in children. To our knowledge, there are no such studies, and certainly not in children with DCD.

The design of this project aims to address the 3 aforementioned voids in the literature. We will examine the effect of an acute exercise intervention on motor learning (adaptation and consolidation) while examining both behavioral and brain imaging outcomes. Providing such knowledge will constitute an important advancement in (a) better characterizing children with DCD, (b) establishing acute exercise as a potential intervention to improve motor learning and attention, and (c) designing educational materials for educators and families related to children with DCD. There is crucial need to increase awareness related to DCD so children with DCD can be identified and diagnosed while providing them with effective and feasible intervention options (Missiuna & Campbell, 2014; Plata & Guerra, 2009).

Social interest

Although DCD have been mainly associated with motor coordination problems, this coordination issues may in turn lead to several health problems for these children like: over-weight or obesity (Beutum et al., 2013; Faught et al., 2013), lower physical activity and higher cardiovascular disease risk (Cairney et al., 2011, 2017), low self-esteem (Cairney et al., 2007; Poulsen et al., 2008), social isolation (Katartzi & Vlachopoulos, 2011), higher anxiety (Piek et al., 2008; Pratt & Hill, 2011) and depression (Dewey et al., 2002). Despite the relevance of the consequences associated with this disorder, DCD is not well known, diagnosed and treated in the Catalan and Spanish societies (Plata & Guerra, 2009). We would argue that improved

knowledge of this disorder by teachers, parents and physical education professionals could play a major role in the early detection of possible DCD cases. In contrast, and because of this lack of knowledge, most cases might go undetected and therefore untreated, although intervention in this disorder, as posed before, is critically necessary. Furthermore, even when a probable DCD case is detected intervention protocols are not well established and, therefore, they could result inefficient o even ineffective.

The present research project will contribute to lessen the main social problems related to DCD via several actions. First, improving knowledge of this disorder by the teaching community, parents and physical education professionals; second, providing means to identify possible DCD cases; and third, providing intervention means to improve learning in children with DCD.

By characterizing DCD in the Catalan population and through the definition of a predictive model of the DCD, we may help teachers and parents identifying those children at risk or high probability of presenting DCD. Guidelines for the diagnosis of this disorder will be generated and distributed via workshops at the different schools. In addition, characterizing DCD in Catalunya is a first step to obtain conclusions regarding these children needs and therefore guide proper interventions. We will deliver two educational workshops at each school, one at the beginning of the data collection and another at the end of the project. Initially, we will introduce the MABC check list so teachers and parents know how to identify probable DCD. At the end of the project, we will share the results emphasizing factors that predict DCD and effects of acute exercise on learning in children with and without DCD.

In future research, larger sample recruitment with multi-site collaboration across the nation (we already have interests from schools in Madrid, Andalucía, Galicia, Valencia, Cantabria and, of course, Catalunya) could constitute a next step towards obtaining more robust conclusions. Through the characterization of DCD we also aim to establish guidelines for the implementation of cognitive-motor training interventions addressed to physical education professionals. Therefore, improvements in the early identification and intervention means of DCD may be expected as result of the present line of research.

On the other hand, one of the main contributions of the present research project will be the generated evidence regarding the effects of exercise-based intervention on DCD children's motor learning. Knowledge about the effect of acute exercise on motor adaptation and consolidation in children with DCD, may guide physical exercise interventions to improve motor learning. Knowledge of exercise effects on learning may also guide short physical exercise interventions ("energizers") as a future technique to enhance learning and attention in the classroom. Previous researchers have successfully implemented such technique in TD children, were improvements in attention and academic performance have been observed (Mahar et al., 2006; Mullender-Wijnsma et al., 2016; Vazou & Skrade, 2017). Because children with DCD have been described as less attentive (Fong, Tsang, & Ng, 2012; Reynolds et al., 2017), and exercise enhances attention (Chang et al., 2012; Ciria, Perakakis, Luque-Casado, Morato, & Sanabria, 2017; Medina et al., 2010), exercise may not only improve motor learning but also other forms of learning. In fact, school-related learning is associated with executive functions such as attention (Blair, Knipe, & Gamson, 2008; Diamond, Barnett, Thomas, &

Munro, 2007) but also with motor learning such as adaptation and consolidation of visuo-motor integration tasks. That is, there is a close relationship between the development of the brain substrates responsible for motor learning and those of executive functions (Diamond, 2000). Because difficulties in motor control and learning in DCD children have been related to various health and quality of life issues, improving their learning and attention through short exercise bouts could carry a much larger impact in the lives of children with DCD.

Finally, the key-enabling neuroimaging technology that we propose is versatile and is applicable to other clinical (e.g. neuro-critical care monitoring, cognitive disorders) and neuroscience (e.g. bi-lingualism, coupling of electrophysiology with hemodynamics) questions enlarging the socio-economic impact of the proposed research. Development of the hybrid system derived from our project will be the responsibility of Dr. Durduran's team in ICFO. ICFO has a strong in-house Knowledge & Technology Transfer (KTT) Team, through which it conducts many relevant research collaborations with industrial partners. ICFO holds a portfolio of more than 90 patent families and has relevant experience in more than 50 industrial projects with different corporations. ICFO is also very proactive in fostering entrepreneurial activities and spin-off creation and, relevant to this project, HemoPhotonics SL is a spin-off of Prof Durduran's group. The center offers its researchers the Launchpad, a space and support structure, which allows innovative ideas to develop into new technology spinoffs. The KTT Team is also responsible for the IP management and exploitation of most of ICFO's funded projects and consortia.

REFERENCES

Adams, I. L. J., Lust, J. M., Wilson, P. H., & Steenbergen, B. (2014). Compromised motor control in children with DCD: A deficit in the internal model?-A systematic review. *Neuroscience and Biobehavioral Reviews*, *47*, 225–244. https://doi.org/10.1016/j.neubiorev.2014.08.011

Angulo-Barroso, R., Ferrer-Uris, B., & Busquets, A. (2019). Enhancing Children's Motor Memory Retention Through Acute Intense Exercise : Effects of Different Exercise Durations. *Frontiers in Psychology*, *10*(August), 1–9. https://doi.org/10.3389/fpsyg.2019.02000

Angulo-Barroso, R. M., Peciña, S., Lin, X., Li, M., Sturza, J., Shao, J., & Lozoff, B. (2017). Implicit learning and emotional responses in nine-month-old infants. *Cognition and Emotion*, *31*(5), 1031–1040. https://doi.org/10.1080/02699931.2016.1179624

Angulo-Barroso, R. M. R., Peirano, P., Algarin, C., Kaciroti, N., & Lozoff, B. (2013). Motor Activity and intra-individual variability according to sleep-wake states in preschool-aged children with iron-deficiency anemia in infancy. *Early Human Development*, *89*(Fondecyt 1110513), 1025–1031. https://doi.org/10.1016/j.earlhumdev.2013.08.014

Armstrong, N., & Fawkner, S. G. (2007). Aerobic Fitness. In N. Armstrong (Ed.), *Paediatric exercise physiology* (pp. 161–187). Churchill Livingstone. Retrieved from https://www.sciencedirect.com/science/book/9780443102608

Batey, C. A., Missiuna, C. A., Timmons, B. W., Hay, J. A., Faught, B. E., & Cairney, J. (2014). Self-efficacy toward physical activity and the physical activity behavior of children with and without Developmental Coordination Disorder. *Human Movement Science*, *36*, 258–271. https://doi.org/10.1016/j.humov.2013.10.003

Benítez-Porres, J., López-Fernández, I., Raya, J. F., Álvarez Carnero, S., Alvero-Cruz, J. R., & Álvarez Carnero, E. (2016). Reliability and Validity of the PAQ-C Questionnaire to Assess Physical Activity in Children. *Journal of School Health*, *86*(9), 677–685. https://doi.org/10.1111/josh.12418

Betts, J., Mckay, J., Maruff, P., & Anderson, V. (2006). The development of sustained attention in children: The effect of age and task load. *Child Neuropsychology*, *12*(3), 205–221. https://doi.org/10.1080/09297040500488522

Beutum, M. N., Cordier, R., & Bundy, A. (2013). Comparing Activity Patterns, Biological, and Family Factors in Children with and Without Developmental Coordination Disorder. *Physical & Occupational Therapy In Pediatrics*, *33*(2), 174–185. https://doi.org/10.3109/01942638.2012.747585

Blair, C., Knipe, H., & Gamson, D. (2008). Is There a Role for Executive Functions in the Development of Mathematics Ability? *Mind, Brain, and Education*, *2*(2), 80–89. https://doi.org/10.1111/j.1751-228X.2008.00036.x

Blank, R., Smits-Engelsman, B. C. M., Polatajko, H., & Wilson, P. H. (2012). European Academy for Childhood Disability (EACD): Recommendations on the definition, diagnosis and intervention of developmental coordination disorder (long version). *Developmental Medicine and Child Neurology*, *54*(1), 54–93. https://doi.org/10.1111/j.1469-8749.2011.04171.x

Blasi, A., Lloyd-Fox, S., Johnson, M. H., & Elwell, C. (2014). Test-retest reliability of functional near infrared spectroscopy in infants. *Neurophotonics*, *1*(2), 025005. https://doi.org/10.1117/1.NPh.1.2.025005

Bo, J., Block, H. J., Clark, J. E., & Bastian, A. J. (2008). A Cerebellar Deficit in Sensorimotor Prediction Explains Movement Timing Variability. *Journal of Neurophysiology*, *100*(5), 2825–2832. https://doi.org/10.1152/jn.90221.2008

Bo, J., & Lee, C. M. (2013). Motor skill learning in children with Developmental Coordination Disorder. *Research in Developmental Disabilities*, *34*(6), 2047–2055. https://doi.org/10.1016/j.ridd.2013.03.012

Boyd, L. A., Vidoni, E. D., Siengsukon, C. F., & Wessel, B. D. (2009). Manipulating time-to-plan alters patterns of brain activation during the Fitts' task. *Experimental Brain Research*, *194*(4), 527–539. https://doi.org/10.1007/s00221-009-1726-4

Busquets, A., Marina, M., & Angulo-Barroso, R. (2013). Changes in motor strategies across age performing a longswing on the high bar. *Research Quarterly for Exercise and Sport*, *84*(3), 353–362. https://doi.org/10.1080/02701367.2013.810537

Busquets, A., Marina, M., Irurtia, A., Ranz, D., & Angulo-Barroso, R. M. (2011). High bar swing performance in novice adults: Effects of practice and talent. *Research Quarterly for Exercise and Sport*, *82*(1), 9–20. https://doi.org/10.1080/02701367.2011.10599717

Buss, A. T., Fox, N., Boas, D. A., & Spencer, J. P. (2014). Probing the early development of visual working memory capacity with functional near-infrared spectroscopy. *NeuroImage*, *85*, 314–325. https://doi.org/10.1016/j.neuroimage.2013.05.034

Caçola, P., Getchell, N., Srinivasan, D., Alexandrakis, G., & Liu, H. (2018). Cortical activity in fine-motor tasks in children with Developmental Coordination Disorder: A preliminary fNIRS study. *International Journal of Developmental Neuroscience*, *65*, 83–90. https://doi.org/10.1016/j.ijdevneu.2017.11.001

Cairney, J., Hay, J. A., Faught, B. E., & Hawes, R. (2005). Developmental coordination disorder and overweight and obesity in children aged 9–14 y. *International Journal of Obesity*, *29*(4), 369–372. https://doi.org/10.1038/sj.ijo.0802893

Cairney, J., Hay, J. A., Veldhuizen, S., Missiuna, C., & Faught, B. E. (2010). Developmental coordination disorder, sex, and activity deficit over time: A longitudinal analysis of participation trajectories in children with and without coordination difficulties. *Developmental Medicine and Child Neurology*, *52*(3), 67–72. https://doi.org/10.1111/j.1469-8749.2009.03520.x

Cairney, J., Hay, J., Mandigo, J., Wade, T., Faught, B. E., & Flouris, A. (2007). Developmental coordination disorder and reported enjoyment of physical education in children. *European Physical Education Review*, *13*(1), 81–98. https://doi.org/10.1177/1356336X07072678

Cairney, J., Hay, J., Veldhuizen, S., & Faught, B. E. (2011). Trajectories of cardiorespiratory fi tness in children with and without developmental coordination disorder: A longitudinal analysis. *British Journal of Sports Medicine*, *45*(15), 1196–1201. https://doi.org/10.1136/bjsm.2009.069880

Cairney, J., Veldhuizen, S., King-Dowling, S., Faught, B. E., & Hay, J. (2017). Tracking cardiorespiratory fitness and physical activity in children with and without motor

coordination problems. *Journal of Science and Medicine in Sport*, *20*(4), 380–385. https://doi.org/10.1016/j.jsams.2016.08.025

Cao, J., Khan, B., Hervey, N., Tian, F., Delgado, M. R., Clegg, N. J., … Alexandrakis, G. (2015). Evaluation of cortical plasticity in children with cerebral palsy undergoing constraint-induced movement therapy based on functional near-infrared spectroscopy. *Journal of Biomedical Optics*, *20*(4), 046009. https://doi.org/10.1117/1.JBO.20.4.046009

Catroppa, C., & Anderson, V. (1999). Attentional Skills in the Acute Phase Following Pediatric Traumatic Brain Injury. *Child Neuropsychology (Neuropsychology, Development and Cognition: Section C)*, *5*(4), 251–264. https://doi.org/10.1076/0929-7049(199912)05:04;1-R;FT251

Cermak, S. A., Katz, N., Weintraub, N., Steinhart, S., Raz-Silbiger, S., Munoz, M., & Lifshitz, N. (2015). Participation in Physical Activity, Fitness, and Risk for Obesity in Children with Developmental Coordination Disorder: A Cross-cultural Study. *Occupational Therapy International*, *22*(4), 163–173. https://doi.org/10.1002/oti.1393

Chang, Y.-K., Liu, S., Yu, H.-H., & Lee, Y.-H. (2012). Effect of acute exercise on executive function in children with attention deficit hyperactivity disorder. *Archives of Clinical Neuropsychology : The Official Journal of the National Academy of Neuropsychologists*, *27*(2), 225–237. https://doi.org/10.1093/arclin/acr094

Chia, L. C., Licari, M. K., Guelfi, K. J., & Reid, S. L. (2014). Investigation of treadmill and overground running: Implications for the measurement of oxygen cost in children with developmental coordination disorder. *Gait and Posture*, *40*(3), 464–470. https://doi.org/10.1016/j.gaitpost.2014.05.054

Chuang, L. Y., Tsai, Y. J., Chang, Y. K., Huang, C. J., & Hung, T. M. (2015). Effects of acute aerobic exercise on response preparation in a Go/No Go Task in children with ADHD: An ERP study. *Journal of Sport and Health Science*, *4*(1), 82–88. https://doi.org/10.1016/j.jshs.2014.11.002

Ciria, L. F., Perakakis, P., Luque-Casado, A., Morato, C., & Sanabria, D. (2017). The relationship between sustained attention and aerobic fitness in a group of young adults. *PeerJ*, *5*, e3831. https://doi.org/10.7717/peerj.3831

Cohen, J. (1988). *Statistical power analysis for the behavioral sciences*. Hillsdale (N.J.) : Lawrence Erlbaum Associates. Retrieved from http://cataleg.ub.edu/record=b1105334~S1*spi

Conners, C. K. (2008). *Conners Comprehensive Behavior Rating Scales*. Pearson Clinical & Talent Assessement España.

Contreras-Vidal, J. L., Bo, J., Boudreau, J. P., & Clark, J. E. (2005). Development of visuomotor representations for hand movement in young children. *Experimental Brain Research*, *162*(2), 155–164. https://doi.org/10.1007/s00221-004-2123-7

Dal Maso, F., Desormeau, B., Boudrias, M. H., & Roig, M. (2018). Acute cardiovascular exercise promotes functional changes in cortico-motor networks during the early stages of motor memory consolidation. *NeuroImage*, *174*(March), 380–392. https://doi.org/10.1016/j.neuroimage.2018.03.029

Debrabant, J., Gheysen, F., Caeyenberghs, K., Van Waelvelde, H., & Vingerhoets, G. (2013). Neural underpinnings of impaired predictive motor timing in children with

Developmental Coordination Disorder. *Research in Developmental Disabilities*, *34*(5), 1478–1487. https://doi.org/10.1016/j.ridd.2013.02.008

Derosière, G., Dalhoumi, S., Perrey, S., Dray, G., & Ward, T. (2014). Towards a near infrared spectroscopy-based estimation of operator attentional state. *PLoS ONE*, *9*(3). https://doi.org/10.1371/journal.pone.0092045

Dewey, D., Kaplan, B. J., Crawford, S. G., & Wilson, B. N. (2002). Developmental coordination disorder: Associated problems in attention, learning, and psychosocial adjustment. *Human Movement Science*, *21*(5–6), 905–918. https://doi.org/10.1016/S0167-9457(02)00163-X

Diamond, A. (2000). Close interrelation of motor development and cognitive development and of the cerebellum and prefrontal cortex. *Child Development*, *71*(1), 44–56. Retrieved from http://www.ncbi.nlm.nih.gov/pubmed/10836557

Diamond, A., Barnett, W. S., Thomas, J., & Munro, S. (2007). Preschool program improves cognitive control. *Science (New York, N.Y.)*, *318*(5855), 1387–1388. https://doi.org/10.1126/science.1151148

Duncan, A., Meek, J. H., Clemence, M., Elwell, C. E., Fallon, P., Tyszczuk, L., … Delpy, D. T. (1996). Measurement of cranial optical path length as a function of age using phase resolved near infrared spectroscopy. *Pediatric Research*, *39*(5), 889–894. https://doi.org/10.1203/00006450-199605000-00025

Durduran, T., Choe, R., Baker, W. B., & Yodh, A. G. (2010). Diffuse optics for tissue monitoring and tomography. *Reports on Progress in Physics*. Rep Prog Phys. https://doi.org/10.1088/0034-4885/73/7/076701

Durduran, T., Kristoffersen, A., Varma, H., Valdes, C., & Culver, J. (2013). Speckle contrast optical tomography.

Durduran, Turgut, & Yodh, A. G. (2014, January 15). Diffuse correlation spectroscopy for non-invasive, micro-vascular cerebral blood flow measurement. *NeuroImage*. Academic Press Inc. https://doi.org/10.1016/j.neuroimage.2013.06.017

Fassbender, C., Murphy, K., Foxe, J. J., Wylie, G. R., Javitt, D. C., Robertson, I. H., & Garavan, H. (2004). A topography of executive functions and their interactions revealed by functional magnetic resonance imaging. *Cognitive Brain Research*, *20*(2), 132–143. https://doi.org/10.1016/j.cogbrainres.2004.02.007

Faught, B. E., Demetriades, S., Hay, J., & Cairney, J. (2013). Does relative body fat influence the Movement ABC-2 assessment in children with and without developmental coordination disorder? *Research in Developmental Disabilities*, *34*(12), 4433–4438. https://doi.org/10.1016/j.ridd.2013.09.016

Ferrer-Uris, B. (2017). *Aprendizaje de habilidades perceptivo-motoras: Efectos de la edad y el ejercicio*. Universitat de Barcelona. Retrieved from http://diposit.ub.edu/dspace/handle/2445/115315#.Ws0gkr3AQnQ.mendeley

Ferrer-Uris, B., Busquets, A., & Angulo-Barroso, R. (2018). Adaptation and Retention of a Perceptual-Motor Task in Children: Effects of a Single Bout of Intense Endurance Exercise. *Journal of Sport and Exercise Psychology*, 1–9. https://doi.org/10.1123/jsep.2017-0044

Ferrer-Uris, B., Busquets, A., Lopez-Alonso, V., Fernandez-del-Olmo, M., & Angulo-Barroso, R.

(2017). Enhancing consolidation of a rotational visuomotor adaptation task through acute exercise. *PLOS ONE*, *12*(4), 1–18. https://doi.org/10.1371/journal.pone.0175296

Fong, S. S. M., Chung, J. W. Y., Cheng, Y. T. Y., Yam, T. T. T., Chiu, H.-C., Fong, D. Y. T., … Ng, S. S. M. (2016). Attention during functional tasks is associated with motor performance in children with developmental coordination disorder. *Medicine*, *95*(37), e4935. https://doi.org/10.1097/MD.0000000000004935

Fong, S. S. M., Lee, V. Y. L., Chan, N. N. C., Chan, R. S. H., Chak, W. K., & Pang, M. Y. C. (2011). Motor ability and weight status are determinants of out-of-school activity participation for children with developmental coordination disorder. *Research in Developmental Disabilities*, *32*(6), 2614–2623. https://doi.org/10.1016/j.ridd.2011.06.013

Fong, S. S. M., Tsang, W. W. N., Cheng, Y. T. Y., Ki, W. Y., Ma, A. W. W., & Macfarlane, D. J. (2015). Single-channel Electroencephalographic Recording in Children with Developmental Coordination Disorder: Validity and influence of Eye Blink Artifacts. *Journal of Novel Physiotherapies*, *05*(04). https://doi.org/10.4172/2165-7025.1000270

Fong, S. S. M., Tsang, W. W. N., & Ng, G. Y. F. (2012). Altered postural control strategies and sensory organization in children with developmental coordination disorder. *Human Movement Science*, *31*(5), 1317–1327. https://doi.org/10.1016/j.humov.2011.11.003

Fortenbaugh, F. C., DeGutis, J., & Esterman, M. (2017). Recent theoretical, neural, and clinical advances in sustained attention research. *Annals of the New York Academy of Sciences*, *1396*(1), 70–91. https://doi.org/10.1111/nyas.13318

Gheysen, F., Van Waelvelde, H., & Fias, W. (2011). Impaired visuo-motor sequence learning in Developmental Coordination Disorder. *Research in Developmental Disabilities*, *32*(2), 749–756. https://doi.org/10.1016/j.ridd.2010.11.005

Gomez, A., & Sirigu, A. (2015). Developmental coordination disorder: Core sensori-motor deficits, neurobiology and etiology. *Neuropsychologia*, *79*, 272–287. https://doi.org/10.1016/j.neuropsychologia.2015.09.032

Green, D., Baird, G., & Sugden, D. (2006). A pilot study of psychopathology in Developmental Coordination Disorder. *Child: Care, Health and Development*, *32*(6), 741–750. https://doi.org/10.1111/j.1365-2214.2006.00684.x

Gueze, R. H., Jongmans, M. J., Schoemaker, M. M., & Smits-Engelsman, B. C. M. (2001). Clinical and research diagnostic criteria for developmental coordination disorder: a review and discussion. *Human Movement Science*, *20*(1–2), 7–47. Retrieved from http://www.ncbi.nlm.nih.gov/pubmed/11471398

Harris, S. R., Mickelson, E. C. R., & Zwicker, J. G. (2015). Diagnosis and management of developmental coordination disorder. *Canadian Medical Association Journal*, *187*(9), 659–665. https://doi.org/10.1503

Henderson, S. E., Sugden, D. A., & Barnett, A. (2007). *Movement Assessment Battery for Children-2 - Second Edition*. Retrieved from http://www.pearsonclinical.com/therapy/products/100000433/movement-assessment-battery-for-children-second-edition-movement-abc-2.html

Henriques, D. Y. P., & Cressman, E. K. (2012). Visuomotor adaptation and proprioceptive recalibration. *Journal of Motor Behavior*, *44*(6), 435–444. https://doi.org/10.1080/00222895.2012.659232

Hillier, S. (2007). Intervention for children with developmental coordination disorder : a systematic review. *The Internet Journal of Allied Health Sciences and Practice*, *5*(3), 1–11. Retrieved from http://ijahsp.nova.edu/articles/vol5num3/hillier.pdf

Hötting, K., & Röder, B. (2013). Beneficial effects of physical exercise on neuroplasticity and cognition. *Neuroscience and Biobehavioral Reviews*, *37*(9), 2243–2257. https://doi.org/10.1016/j.neubiorev.2013.04.005

Hyodo, K., Dan, I., Suwabe, K., Kyutoku, Y., Yamada, Y., Akahori, M., … Soya, H. (2012). Acute moderate exercise enhances compensatory brain activation in older adults. *Neurobiology of Aging*, *33*(11), 2621–2632. https://doi.org/10.1016/j.neurobiolaging.2011.12.022

Imai, M., Watanabe, H., Yasui, K., Kimura, Y., Shitara, Y., Tsuchida, S., … Taga, G. (2014). Functional connectivity of the cortex of term and preterm infants and infants with Down's syndrome. *NeuroImage*, *85*, 272–278. https://doi.org/10.1016/j.neuroimage.2013.04.080

Imamizu, H., Kuroda, T., Miyauchi, S., Yoshioka, T., & Kawato, M. (2003). Modular organization of internal models of tools in the human cerebellum. *Proceedings of the National Academy of Sciences*, *100*(9), 5461–5466. https://doi.org/10.1073/pnas.0835746100

Imamizu, H., Kuroda, T., Yoshioka, T., & Kawato, M. (2004). Functional Magnetic Resonance Imaging Examination of Two Modular Architectures for Switching Multiple Internal Models. *Journal of Neuroscience*, *24*(5), 1173–1181. https://doi.org/10.1523/JNEUROSCI.4011-03.2004

Imamizu, Hiroshi, Miyauchi, S., Tamada, T., Sasaki, Y., Takino, R., Pütz, B., … Kawato, M. (2000). Human cerebellar activity reflecting an acquired internal model of a new tool. *Nature*, *403*(6766), 192–195. https://doi.org/10.1038/35003194

Iversen, S., Berg, K., Ellertsen, B., & Tønnessen, F.-E. (2005). Motor coordination difficulties in a municipality group and in a clinical sample of poor readers. *Dyslexia (Chichester, England)*, *11*(3), 217–231. Retrieved from http://www.ncbi.nlm.nih.gov/pubmed/16128050

Jarus, T., Ghanouni, P., Abel, R. L., Fomenoff, S. L., Lundberg, J., Davidson, S., … Zwicker, J. G. (2015). Effect of internal versus external focus of attention on implicit motor learning in children with developmental coordination disorder. *Research in Developmental Disabilities*, *37*, 119–126. https://doi.org/10.1016/j.ridd.2014.11.009

Jelsma, D., Ferguson, G. D., Smits-Engelsman, B. C. M., & Geuze, R. H. (2015). Short-term motor learning of dynamic balance control in children with probable Developmental Coordination Disorder. *Research in Developmental Disabilities*, *38*, 213–222. https://doi.org/10.1016/j.ridd.2014.12.027

Kadejso, B., & Gillberg, C. (1999). Developmental Coordination Disorder in Swedish 7-Year-Old Children. *Journal of the American Academy of Child & Adolescent Psychiatry*, *38*(7), 820–828. https://doi.org/10.1097/00004583-199907000-00011

Kagerer, F. A., Bo, J., Contreras-Vidal, J. L., & Clark, J. E. (2004). Visuomotor adaptation in children with developmental coordination disorder. *Motor Control*, *8*(4), 450–460. Retrieved from http://www.ncbi.nlm.nih.gov/pubmed/15585900

Kagerer, F. A., Contreras-Vidal, J. L., Bo, J., & Clark, J. E. (2006). Abrupt, but not gradual visuomotor distortion facilitates adaptation in children with developmental coordination

disorder. *Human Movement Science*, *25*(4–5), 622–633. https://doi.org/10.1016/j.humov.2006.06.003

Kahneman, D. (1973). *Attention and effort* (Vol. 1063). Prentice-Hall Englewood Cliffs, NJ. https://doi.org/10.2307/1421603

Kashiwagi, M., Iwaki, S., Narumi, Y., Tamai, H., & Suzuki, S. (2009). Parietal dysfunction in developmental coordination disorder: A functional MRI study. *NeuroReport*, *20*(15), 1319–1324. https://doi.org/10.1097/WNR.0b013e32832f4d87

Kasuga, S., Telgen, S., Ushiba, J., Nozaki, D., & Diedrichsen, J. (2015). Learning feedback and feedforward control in a mirror-reversed visual environment. *Journal of Neurophysiology*, *114*(4), 2187–2193. https://doi.org/10.1152/jn.00096.2015

Katartzi, E. S., & Vlachopoulos, S. P. (2011). Motivating children with developmental coordination disorder in school physical education: The self-determination theory approach. *Research in Developmental Disabilities*, *32*(6), 2674–2682. https://doi.org/10.1016/j.ridd.2011.06.005

Kawato, M. (1999). Internal models for motor control and trajectory planning. *Current Opinion in Neurobiology*, *9*(6), 718–727. Retrieved from http://www.ncbi.nlm.nih.gov/pubmed/10607637

Kempermann, G., Fabel, K., Ehninger, D., Babu, H., Leal-Galicia, P., Garthe, A., & Wolf, S. A. (2010). Why and how physical activity promotes experience-induced brain plasticity. *Frontiers in Neuroscience*, *4*(DEC), 1–9. https://doi.org/10.3389/fnins.2010.00189

King, B. R., Harring, J. R., Oliveira, M. A., & Clark, J. E. (2011). Statistically characterizing intra- and inter-individual variability in children with Developmental Coordination Disorder. *Research in Developmental Disabilities*, *32*(4), 1388–1398. https://doi.org/10.1016/j.ridd.2010.12.043

King, B. R., Kagerer, F. A., Harring, J. R., Contreras-Vidal, J. L., & Clark, J. E. (2011). Multisensory adaptation of spatial-to-motor transformations in children with developmental coordination disorder. *Experimental Brain Research*, *212*(2), 257–265. https://doi.org/10.1007/s00221-011-2722-z

Kirby, A., Sugden, D., Beveridge, S., & Edwards, L. (2008). Developmental co-ordination disorder (DCD) in adolescents and adults in further and higher education. *Journal of Research in Special Educational Needs*, *8*(3), 120–131. https://doi.org/10.1111/j.1471-3802.2008.00111.x

Kirby, A., Sugden, D., & Purcell, C. (2014). Diagnosing developmental coordination disorders. *Archives of Disease in Childhood*, *99*(3), 292–296. https://doi.org/10.1136/archdischild-2012-303569

Koehl, M., Meerlo, P., Gonzales, D., Rontal, A., Turek, F. W., & Abrous, D. N. (2008). Exercise-induced promotion of hippocampal cell proliferation requires β-endorphin. *The FASEB Journal*, *22*(7), 2253–2262. https://doi.org/10.1096/fj.07-099101

Krakauer, J. W., Ghez, C., & Ghilardi, M. F. (2005). Adaptation to visuomotor transformations: consolidation, interference, and forgetting. *The Journal of Neuroscience : The Official Journal of the Society for Neuroscience*, *25*(2), 473–478. https://doi.org/10.1523/JNEUROSCI.4218-04.2005

Labelle, V., Bosquet, L., Mekary, S., Vu, T. T. M., Smilovitch, M., & Bherer, L. (2014). Fitness Level Moderates Executive Control Disruption during Exercise Regardless of Age. *Journal of Sport and Exercise Psychology*, *36*(3), 258–270. https://doi.org/10.1123/jsep.2013-0115

Lambrick, D., Stoner, L., Grigg, R., & Faulkner, J. (2016). Effects of continuous and intermittent exercise on executive function in children aged 8–10 years. *Psychophysiology*, *53*(9), 1335–1342. https://doi.org/10.1111/psyp.12688

Léger, L. A., Mercier, D., Gadoury, C., & Lambert, J. (1988). The multistage 20 metre shuttle run test for aerobic fitness. *Journal of Sports Sciences*, *6*(2), 93–101. https://doi.org/10.1080/02640418808729800

Lejeune, C., Catale, C., Willems, S., & Meulemans, T. (2013). Intact procedural motor sequence learning in developmental coordination disorder. *Research in Developmental Disabilities*, *34*(6), 1974–1981. https://doi.org/10.1016/j.ridd.2013.03.017

Lejeune, C., Wansard, M., Geurten, M., & Meulemans, T. (2016). Procedural learning, consolidation, and transfer of a new skill in Developmental Coordination Disorder. *Child Neuropsychology*, *22*(2), 143–154. https://doi.org/10.1080/09297049.2014.988608

Lingam, R., Hunt, L., Golding, J., Jongmans, M., & Emond, A. (2009). Prevalence of Developmental Coordination Disorder Using the DSM-IV at 7 Years of Age: A UK Population-Based Study. *PEDIATRICS*, *123*(4), e693–e700. https://doi.org/10.1542/peds.2008-1770

Lloyd-Fox, S., Blasi, A., & Elwell, C. E. (2010). Illuminating the developing brain: The past, present and future of functional near infrared spectroscopy. *Neuroscience and Biobehavioral Reviews*, *34*(3), 269–284. https://doi.org/10.1016/j.neubiorev.2009.07.008

Lloyd-Fox, S., Wu, R., Richards, J. E., Elwell, C. E., & Johnson, M. H. (2015). Cortical activation to action perception is associated with action production abilities in young infants. *Cerebral Cortex*, *25*(2), 289–297. https://doi.org/10.1093/cercor/bht207

Lundbye-Jensen, J., Skriver, K., Nielsen, J. B., & Roig, M. (2017). Acute Exercise Improves Motor Memory Consolidation in Preadolescent Children. *Frontiers in Human Neuroscience*, *11*(April), 1–10. https://doi.org/10.3389/fnhum.2017.00182

Mahar, M. T., Murphy, S. K., Rowe, D. a., Golden, J., Shields, a. T., & Raedeke, T. D. (2006). Effects of a classroom-based program on physical activity and on-task behavior. *Medicine and Science in Sports and Exercise*, *38*(12), 2086–2094. https://doi.org/10.1249/01.mss.0000235359.16685.a3

Mandrick, K., Derosiere, G., Dray, G., Coulon, D., Micallef, J.-P., & Perrey, S. (2013). Utilizing slope method as an alternative data analysis for functional near-infrared spectroscopy-derived cerebral hemodynamic responses. *International Journal of Industrial Ergonomics*, *43*(4), 335–341. https://doi.org/10.1016/J.ERGON.2013.05.003

McMorris, T., Collard, K., Corbett, J., Dicks, M., & Swain, J. P. (2008). A test of the catecholamines hypothesis for an acute exercise–cognition interaction. *Pharmacology Biochemistry and Behavior*, *89*(1), 106–115. https://doi.org/10.1016/j.pbb.2007.11.007

McMorris, T., & Hale, B. J. (2012). Differential effects of differing intensities of acute exercise on speed and accuracy of cognition: A meta-analytical investigation. *Brain and Cognition*, *80*(3), 338–351. https://doi.org/10.1016/j.bandc.2012.09.001

Medina, J. A., Netto, T. L. B., Muszkat, M., Medina, A. C., Botter, D., Orbetelli, R., … Miranda, M. C. (2010). Exercise impact on sustained attention of ADHD children, methylphenidate effects. *ADHD Attention Deficit and Hyperactivity Disorders*, *2*(1), 49–58. https://doi.org/10.1007/s12402-009-0018-y

Miall, R. C., Jenkinson, N., & Kulkarni, K. (2004). Adaptation to rotated visual feedback: a re-examination of motor interference. *Experimental Brain Research*, *154*(1999), 201–210. https://doi.org/10.1007/s00221-003-1630-2

Milham, M. P., Banich, M. T., Claus, E. D., & Cohen, N. J. (2003). Practice-related effects demonstrate complementary roles of anterior cingulate and prefrontal cortices in attentional control. *NeuroImage*, *18*(2), 483–493. Retrieved from http://www.ncbi.nlm.nih.gov/pubmed/12595201

Missiuna, C., & Campbell, W. N. (2014). Psychological Aspects of Developmental Coordination Disorder: Can We Establish Causality? *Current Developmental Disorders Reports*, *1*(2), 125–131. https://doi.org/10.1007/s40474-014-0012-8

Moriguchi, Y., & Hiraki, K. (2013). Prefrontal cortex and executive function in young children: a review of NIRS studies. *Frontiers in Human Neuroscience*, *7*(December), 1–9. https://doi.org/10.3389/fnhum.2013.00867

Mullender-Wijnsma, M. J., Hartman, E., de Greeff, J. W., Doolaard, S., Bosker, R. J., & Visscher, C. (2016). Physically Active Math and Language Lessons Improve Academic Achievement: A Cluster Randomized Controlled Trial. *Pediatrics*, *137*(3), e20152743. https://doi.org/10.1542/peds.2015-2743

Nishiyori, R., Bisconti, S., Meehan, S. K., & Ulrich, B. D. (2016). Developmental changes in motor cortex activity as infants develop functional motor skills. *Developmental Psychobiology*, *58*(6), 773–783. https://doi.org/10.1002/dev.21418

Piek, J. P., Dawson, L., Smith, L. M., & Gasson, N. (2008). The role of early fine and gross motor development on later motor and cognitive ability. *Human Movement Science*, *27*(5), 668–681. https://doi.org/10.1016/j.humov.2007.11.002

Piepmeier, A. T., Shih, C.-H., Whedon, M., Williams, L. M., Davis, M. E., Henning, D. a., … Etnier, J. L. (2015). The effect of acute exercise on cognitive performance in children with and without ADHD. *Journal of Sport and Health Science*, *4*(1), 97–104. https://doi.org/10.1016/j.jshs.2014.11.004

Plata, R., & Guerra, G. (2009). El niño con trastorno del desarrollo de la coordinación ¿Un desconocido en nuestra comunidad? *Norte de Salud Mental*, *33*, 18–30.

Pless, M., & Carlsson, M. (2000). Effects of motor skill interventions on developmental coordination disorder: a meta-analysis. *Physical Activity Quarterly*.

Posner, M. (1990). The Attention System Of The Human Brain. *Annual Review of Neuroscience*, *13*(1), 25–42. https://doi.org/10.1146/annurev.neuro.13.1.25

Poulsen, A. A., Ziviani, J. M., Johnson, H., & Cuskelly, M. (2008). Loneliness and life satisfaction of boys with developmental coordination disorder: The impact of leisure participation and perceived freedom in leisure. *Human Movement Science*, *27*(2), 325–343. https://doi.org/10.1016/j.humov.2008.02.004

Pratt, M. L., & Hill, E. L. (2011). Anxiety profiles in children with and without developmental

coordination disorder. *Research in Developmental Disabilities*, *32*(4), 1253–1259. https://doi.org/10.1016/j.ridd.2011.02.006

Preston, N., Magallón, S., Hill, L. J. B., Andrews, E., Ahern, S. M., & Mon-Williams, M. (2017). A systematic review of high quality randomized controlled trials investigating motor skill programmes for children with developmental coordination disorder. *Clinical Rehabilitation*, *31*(7), 857–870. https://doi.org/10.1177/0269215516661014

Querne, L., Berquin, P., Vernier-Hauvette, M.-P., Fall, S., Deltour, L., Meyer, M.-E., & de Marco, G. (2008). Dysfunction of the attentional brain network in children with Developmental Coordination Disorder: A fMRI study. *Brain Research*, *1244*, 89–102. https://doi.org/10.1016/j.brainres.2008.07.066

Raz, A. (2004). Anatomy of attentional networks. *Anatomical Record - Part B New Anatomist*, *281*(1), 21–36. https://doi.org/10.1002/ar.b.20035

Reynolds, J. E., Licari, M. K., Reid, S. L., Elliott, C., Winsor, A. M., Bynevelt, M., & Billington, J. (2017). Reduced relative volume in motor and attention regions in developmental coordination disorder: A voxel-based morphometry study. *International Journal of Developmental Neuroscience*, *58*, 59–64. https://doi.org/10.1016/j.ijdevneu.2017.01.008

Roche, R., Viswanathan, P., Clark, J. E., & Whitall, J. (2016). Children with developmental coordination disorder (DCD) can adapt to perceptible and subliminal rhythm changes but are more variable. *Human Movement Science*, *50*, 19–29. https://doi.org/10.1016/j.humov.2016.09.003

Ruiz, L. M., & Graupera-Sanz, J. L. (2012). *Batería de evaluación del movimiento para niños-2 (MABC-2)*. Madrid: Pearson Clinical & Talent Assessement España.

Scholkmann, F., & Wolf, M. (2013). General equation for the differential pathlength factor of the frontal human head depending on wavelength and age. *Journal of Biomedical Optics*, *18*(10), 105004. https://doi.org/10.1117/1.JBO.18.10.105004

Schwarz, L., & Kindermann, W. (1990). Beta-endorphin, adrenocorticotropic hormone, cortisol and catecholamines during aerobic and anaerobic exercise. *European Journal of Applied Physiology and Occupational Physiology*, *61*(3–4), 165–171. Retrieved from http://www.ncbi.nlm.nih.gov/pubmed/2178088

Skriver, K., Roig, M., Lundbye-Jensen, J., Pingel, J., Helge, J. W., Kiens, B., & Nielsen, J. B. (2014). Acute exercise improves motor memory: Exploring potential biomarkers. *Neurobiology of Learning and Memory*, *116*, 46–58. https://doi.org/10.1016/j.nlm.2014.08.004

Smits-Engelsman, B. C. M., Jelsma, L. D., Ferguson, G. D., & Geuze, R. H. (2015). Motor Learning: An Analysis of 100 Trials of a Ski Slalom Game in Children with and without Developmental Coordination Disorder. *Plos One*, *10*(10), e0140470. https://doi.org/10.1371/journal.pone.0140470

Smits-Engelsman, B. C. M., & Wilson, P. H. (2013). Noise, variability, and motor performance in developmental coordination disorder. *Developmental Medicine and Child Neurology*, *55*(SUPPL.4), 69–72. https://doi.org/10.1111/dmcn.12311

Smits-Engelsman, B. C. M., Wilson, P. H., Westenberg, Y., & Duysens, J. (2003). Fine motor deficiencies in children with developmental coordination disorder and learning disabilities: an underlying open-loop control deficit. *Human Movement Science*, *22*(4–5), 495–513. Retrieved from http://www.ncbi.nlm.nih.gov/pubmed/14624830

Summers, J., Larkin, D., & Dewey, D. (2008). Activities of daily living in children with developmental coordination disorder: Dressing, personal hygiene, and eating skills. *Human Movement Science*, *27*(2), 215–229. https://doi.org/10.1016/j.humov.2008.02.002

Takahashi, C. D., Nemet, D., Rose-Gottron, C. M., Larson, J. K., Cooper, D. M., & Reinkensmeyer, D. J. (2006). Effect of muscle fatigue on internal model formation and retention during reaching with the arm. *Journal of Applied Physiology (Bethesda, Md. : 1985)*, *100*(2), 695–706. https://doi.org/10.1152/japplphysiol.00140.2005

Taubert, M., Villringer, A., & Lehmann, N. (2015). Endurance Exercise as an "Endogenous" Neuro-enhancement Strategy to Facilitate Motor Learning. *Frontiers in Human Neuroscience*, *9*(December), 1–16. https://doi.org/10.3389/fnhum.2015.00692

Tsai, C. L. (2009). The effectiveness of exercise intervention on inhibitory control in children with developmental coordination disorder: Using a visuospatial attention paradigm as a model. *Research in Developmental Disabilities*, *30*(6), 1268–1280. https://doi.org/10.1016/j.ridd.2009.05.001

Tsiotra, G. D., Flouris, A. D., Koutedakis, Y., Faught, B. E., Nevill, A. M., Lane, A. M., & Skenteris, N. (2006). A Comparison of Developmental Coordination Disorder Prevalence Rates in Canadian and Greek Children. *Journal of Adolescent Health*, *39*(1), 125–127. https://doi.org/10.1016/j.jadohealth.2005.07.011

Valdes, C. P., Varma, H. M., Kristoffersen, A. K., Dragojevic, T., Culver, J. P., & Durduran, T. (2014). Speckle contrast optical spectroscopy, a non-invasive, diffuse optical method for measuring microvascular blood flow in tissue. *Biomedical Optics Express*, *5*(8), 2769. https://doi.org/10.1364/boe.5.002769

Valentín-Gudiol, M., Mattern-Baxter, K., Girabent-Farrés, M., Bagur-Calafat, C., Hadders-Algra, M., & Angulo-Barroso, R. M. (2017, July 29). Treadmill interventions in children under six years of age at risk of neuromotor delay. *Cochrane Database of Systematic Reviews*. John Wiley and Sons Ltd. https://doi.org/10.1002/14651858.CD009242.pub3

Vanderwert, R. E., & Nelson, C. A. (2014). The use of near-infrared spectroscopy in the study of typical and atypical development. *NeuroImage*, *85*, 264–271. https://doi.org/10.1016/j.neuroimage.2013.10.009

Varma, H. M., Valdes, C. P., Kristoffersen, A. K., Culver, J. P., & Durduran, T. (2014). Speckle contrast optical tomography: A new method for deep tissue three-dimensional tomography of blood flow. *Biomedical Optics Express*, *5*(4), 1275. https://doi.org/10.1364/boe.5.001275

Vazou, S., & Skrade, M. A. B. (2017). Intervention integrating physical activity with math: Math performance, perceived competence, and need satisfaction. *International Journal of Sport and Exercise Psychology*, *15*(5), 508–522. https://doi.org/10.1080/1612197X.2016.1164226

Vereijken, B., Van Emmerik, R. E. A., Bongaardt, R., Beek, W. J., & Newell, K. M. (1997). Changing coordinative structures in complex skill acquisition. *Human Movement Science*, *16*(6), 823–844. https://doi.org/10.1016/S0167-9457(97)00021-3

Visser, J. (2003). Developmental coordination disorder: a review of research on subtypes and comorbidities. *Human Movement Science*, *22*(4–5), 479–493.

https://doi.org/10.1016/j.humov.2003.09.005

Wang, T.-N., Tseng, M.-H., Wilson, B. N., & Hu, F.-C. (2009). Functional performance of children with developmental coordination disorder at home and at school. *Developmental Medicine & Child Neurology*, *51*(10), 817–825. https://doi.org/10.1111/j.1469-8749.2009.03271.x

Wilmut, K., & Wann, J. (2008). The use of predictive information is impaired in the actions of children and young adults with Developmental Coordination Disorder. *Experimental Brain Research*, *191*(4), 403–418. https://doi.org/10.1007/s00221-008-1532-4

Wilson, B. N., Crawford, S. G., Green, D., Roberts, G., Aylott, A., & Kaplan, B. J. (2009). Psychometric properties of the revised developmental coordination disorder questionnaire. *Physical and Occupational Therapy in Pediatrics*, *29*(2), 182–202. https://doi.org/10.1080/01942630902784761

Wilson, P. H., & Butson, M. (2007). Deficits underlying DCD. In R. H. Geuze, J.-M. Albaret, J. Visser, P. H. Wilson, H. Polatajko, & D. A. Sugden (Eds.), *Developmental Coordination Disorder. A review of current approaches* (pp. 111–138). Solal Éditeurs.

Wilson, P. H., Maruff, P., & Lum, J. (2003). Procedural learning in children with developmental coordination disorder. *Human Movement Science*, *22*(4–5), 515–526. Retrieved from http://www.ncbi.nlm.nih.gov/pubmed/14624831

Wilson, P. H., Ruddock, S., Smits-Engelsman, B. C. M., Polatajko, H., & Blank, R. (2013). Understanding performance deficits in developmental coordination disorder: A meta-analysis of recent research. *Developmental Medicine and Child Neurology*, *55*(3), 217–228. https://doi.org/10.1111/j.1469-8749.2012.04436.x

Wilson, P. H., Smits-Engelsman, B. C. M., Caeyenberghs, K., & Steenbergen, B. (2017). Toward a Hybrid Model of Developmental Coordination Disorder. *Current Developmental Disorders Reports*, *4*(3), 64–71. https://doi.org/10.1007/s40474-017-0115-0

Winter, B., Breitenstein, C., Mooren, F. C., Voelker, K., Fobker, M., Lechtermann, A., … Knecht, S. (2007). High impact running improves learning. *Neurobiology of Learning and Memory*, *87*(4), 597–609. https://doi.org/10.1016/j.nlm.2006.11.003

World Health Organization. (1992). The ICD-10 classification of mental and behavioural disorders: clinical descriptions and diagnostic guidelines. Geneva: World Health Organization.

World Health Organization. (2018). Revision of the International Classification of Diseases (ICD-11) - Draft. Geneva: World Health Organization.

Yanagisawa, H., Dan, I., Tsuzuki, D., Kato, M., Okamoto, M., Kyutoku, Y., & Soya, H. (2010). Acute moderate exercise elicits increased dorsolateral prefrontal activation and improves cognitive performance with Stroop test. *NeuroImage*, *50*(4), 1702–1710. https://doi.org/10.1016/j.neuroimage.2009.12.023

Zwicker, J. G., Harris, S. R., & Klassen, A. F. (2013). Quality of life domains affected in children with developmental coordination disorder: A systematic review. *Child: Care, Health and Development*, *39*(4), 562–580. https://doi.org/10.1111/j.1365-2214.2012.01379.x

Zwicker, J. G., Missiuna, C., & Boyd, L. A. (2009). Neural correlates of developmental coordination disorder: A review of hypotheses. *Journal of Child Neurology*, *24*(10), 1273–

1281. https://doi.org/10.1177/0883073809333537

Zwicker, J. G., Missiuna, C., Harris, S. R., & Boyd, L. A. (2010). Brain Activation of Children With Developmental Coordination Disorder is Different Than Peers. *Pediatrics*, *126*(3), e678–e686. https://doi.org/10.1542/peds.2010-0059

Zwicker, J. G., Missiuna, C., Harris, S. R., & Boyd, L. A. (2011). Brain activation associated with motor skill practice in children with developmental coordination disorder: An fMRI study. *International Journal of Developmental Neuroscience*, *29*(2), 145–152. https://doi.org/10.1016/j.ijdevneu.2010.12.002

Zwicker, J. G., Missiuna, C., Harris, S. R., & Boyd, L. A. (2012). Developmental coordination disorder: A review and update. *European Journal of Paediatric Neurology*, *16*(6), 573–581. https://doi.org/10.1016/j.ejpn.2012.05.005

ANNEX 1 (RESEARCHERS' CV)

ANNEX 2 (SCHOOLS)

ANNEX 3 (PARENTS/LEGAL TUTOR INFORMED CONSENT)

INFORMACIÓ I CONSENTIMENT INFORMAT

Institut Nacional d'Educació Física de Catalunya-Barcelona, centre adscrit a la Universitat de Barcelona

Identificació del projecte

Examining the effects of acute exercise on motor learning and brain activity in children with developmental coordination disorder (ExLe-Brain-DCD)

Afirmació sobre l'edat del/de la participant

Vostè és una persona de 18 anys o més i és el pare, mare o tutor legal del d'aquest infant que té una edat compresa entre els 7.5 i els 10.5 anys d'edat. El seu infant ha estat convidat a participar en un projecte d'investigació dirigit per el Dr. Albert Busquets Faciabén i la Dra. Rosa Angulo Barroso del Grup de Recerca en Activitat Física i Salut de l'Institut Nacional d'Educació Física de Catalunya (Barcelona, Espanya).

Objectiu de l'estudi

L'objectiu principal d'aquest estudi és investigar la influència de la realització d'una única sessió d'exercici físic intens sobre la capacitat d'aprenentatge d'infants amb desenvolupament motor típic (TD) i infants amb trastorn del desenvolupament de la coordinació motora (TDC). Concretament, s'estudiarà l'efecte d'una sessió d'exercici sobre la capacitat d'aprenentatge d'una tasca de coordinació ull-mà que serà executada mitjançant un ordinador. S'estudiaran els efectes de l'exercici físic a curt (1 hora), mig (24 hores) i llarg termini (7 dies). Per dur a terme l'estudi, es dividirà als participants en 4 grups: grup d'infants amb desenvolupament típic que realitzarà exercici (EX-TD), grup d'infants amb dificultats coordinatives que realitzarà exercici (EX-TDC), grup d'infants amb desenvolupament típic que no realitzarà exercici (CON-TD) i grup d'infants amb dificultats coordinatives que no realitzarà exercici (CON-TDC). El seu nen/a participarà en un d'aquests grups on s'assignarà de forma aleatòria la condició de realitzar o no exercici previ a la tasca d'aprenentatge.

Procediment experimental

L'estudi consta de 5 sessions diferents. Si vostè accedeix a que el seu infant participi en aquest estudi, els investigadors li administraran les següents proves:

Previ a l'inici de l'estudi, s'avaluarà la idoneïtat dels candidats a participar en l'estudi a través d'uns qüestionaris sobre els seus hàbits de salut, antecedents mèdics, nivell de pràctica d'exercici físic setmanal i sobre l'avaluació de la conducta motriu. Aquests qüestionaris seran complimentats entre els professors de cada infant i els pares/mares/tutors legals del mateix.

Sessió 1: durant la primera sessió de l'estudi, s'administrarà un test per valorar la capacitat cognitiva de l'infant, un test per avaluar el rendiment en les capacitats coordinatives de l'infant i es mesurarà l'alçada i pes de l'infant. En aquesta sessió es demanarà als participants que executin una prova de carrera amb l'objectiu d'avaluar el seu nivell de condició física. La prova

consistirà en desplaçar-se corrent d'un costat a l'altre d'una pista de 20m de llarg, tot seguint el ritme marca per un so. Es demanarà als infants que intentin realitzar el màxim número de trajectes possibles seguint el ritme del so. Per incentivar el seu esforç aquesta tasca serà presentada en forma de joc. Durant la realització de la prova es col·locarà als participants una banda elàstica al pit que captarà la freqüència de batec del cor (pulsòmetre). Aquest dispositiu, totalment inòcul, s'utilitzarà per valorar el nivell d'esforç del participant i preservar la seva seguretat durant tota la prova.

Sessió 2: durant la segona sessió, la qual es realitzarà com a mínim 48h després de la segona, els participants realitzaran tres tasques diferents. Per un costat, es demanarà als participants la realització d'una sessió d'exercici intens, amb un desenvolupament molt semblant a la prova de carrera realitzada a la primera sessió. Els integrants dels grups CON (CON-TD i CON-TDC) no realitzaran aquest exercici i, enlloc seu, romandran en repòs fent alguna activitat tranquil·la a l'aula durant un temps equivalent a l'exercici dels altres grups (25 min). Per altre costat, també es demanarà als participants que realitzin una tasca d'aprenentatge on s'involucrarà la coordinació ull-mà, en la qual els participants controlaran els moviments d'un cercle a la pantalla d'un ordinador mitjançant l'ús d'un joystick, amb un plantejament semblant al d'un videojoc. L'objectiu d'aquesta tasca serà el de moure un cercle a uns punts objectius de la pantalla amb la màxima exactitud i velocitat possibles. Es demanarà als participants que practiquin aquesta tasca durant un temps d'aproximadament 8 minuts. Seguidament, i després d'un temps de repòs d'una hora, es demanarà als participants que tornin a realitzar la tasca d'aprenentatge, aquest cop només durant 3,5 minuts per tal de comprovar el nivell de retenció de l'aprenentatge inicial (test de retenció). Abans i després de realitzar l'exercici o el descans equivalent, es col·locarà sobre el cap dels participants una gorro per mesurar l'activitat de la regió cerebral prefrontal, la qual està íntimament lligada amb l'atenció. Aquest dispositiu és totalment inòcul i mesura l'activitat cerebral a través de l'emissió i captació de llum infraroja, la mateixa que emet, per exemple, un comandament a distància d'un televisor.

Sessions 3 i 4: durant aquestes dues sessions es demanarà, de nou, la realització de dos test de retenció de la tasca d'aprenentatge (un a cada sessió). Aquestes dues sessions es realitzaran 24 h i 7 dies després de la tercera sessió, respectivament. L'infant també portarà el gorro per a la mesura de l'activitat cerebral.

Necessitats materials

Per tal de realitzar les proves de carrera de les sessions 1 i 2, serà necessari que els participants assisteixin amb roba còmode i calçat esportiu. A més, en cas de presentar algun problema de visió corregit mitjançant l'ús d'ulleres, serà necessari que portin les ulleres per tal de realitzar la tasca d'aprenentatge i els seus test de retenció (sessions 2, 3 i 4)

Confidencialitat i protecció de les dades

Tota la informació recollida per aquest estudi és estrictament confidencial La informació recollida provindrà de les proves realitzades al seu infant i dels qüestionaris que li facilitarem. A més, l'observació conductual del nen/a que vostè ens proporciona serà complementada amb informació provinent del mestre responsable de la classe de l'infant o que designi l'escola,

també mitjançant un qüestionari. Les dades personals que obtinguem seran agrupades amb les dades d'altres participants i emmagatzemades de forma confidencial i segura a les instal·lacions de l'Institut Nacional d'Educació Física de Catalunya (INEFC), Barcelona. Únicament els investigadors principals d'aquest projecte i els seus col·laboradors tindran accés als arxius de dades personals protegides. Les dades obtingudes de les proves estaran pseudonimitzades i seran preservades en espais segurs i per a investigació al núvol d'emmagatzematge de dades i en dos discs durs protegits. Seguint les prerrogatives de la ciència d'accés obert, les dades obtingudes i anonimitzades podran fer-se públiques per a usos d'investigació i amb les mateixes condicions que hem establert nosaltres.

Les possibles imatges de vídeo y/o fotografia que s'obtinguin durant la realització de l'estudi seran anonimitzades (s'ocultarà, pixelarà o difuminarà el rostre i qualsevol altra marca que pugui identificar l'infant) i només s'empraran per a l'elaboració d'articles, comunicacions científiques o altres formes de divulgació dels resultats de l'estudi. Es preveu que aquesta difusió dels resultats es realitzarà via revistes científiques (publicació on-line i/o en paper), presentacions científiques (powerpoint o similar) i divulgació al públic en general (web).

Les dades i imatges que s'obtinguin de la investigació no s'utilitzaran per a cap altra finalitat que no sigui l'esmentada en aquest document i passaran a formar part d'un arxiu de dades del que seran els màxim responsables els investigadors principals (Dr. Albert Busquets i Dra. Rosa Angulo). Les dades identificatives i imatges no anonimitzades seran guardades com a mínim durant 5 anys després de la durada de l'estudi i després es procedirà a la seva eliminació.

Vostè podrà exercir en tot moment els drets que estableix la Llei Orgànica 3/2018, de 5 de desembre, de protecció de dades personals i garantia de drets digitals i el Reglament (UE) 2016/679, de 27 d'abril de 2016, de protecció de dades (RGPD). Com a responsable legal de l'infant participant té dret a accedir a totes les seves dades, sol·licitar la rectificació de les dades inexactes o, si escau, sol·licitar-ne la supressió, així com limitar-ne el tractament, oposar-se i retirar el consentiment del seu ús per a determinades finalitats. A més, tant vostè com el seu infant tenen dret a retirar-se en qualsevol moment duna part o de la totalitat de l'estudi sense manifestar la causa o motiu i sense conseqüències. També tenen dret que se'ls aclareixin els possibles dubtes abans d'acceptar participar-hi, i a conèixer els resultats de la investigació.

Aquests drets els podeu exercir mitjançant un escrit a l'adreça postal o mitjançant un missatge de correu electrònic a l'adreça de l'investigador principal Dr. Albert Busquets. Així mateix, us informem del vostre dret a presentar una reclamació davant l'Agència Catalana de Protecció de Dades en cas de qualsevol actuació que considereu que vulnera els vostres drets. A més, De conformitat amb el que estableix la regulació esmentada, la Universitat de Barcelona (amb CIF Q0818001J i domicili a la Gran Via de les Corts Catalanes, 585 -08007 Barcelona) i l'INEFC (amb CIF Q0840010C i domicili a l'Avinguda de l'Estadi 12-22 -08038 Barcelona) com a responsables del tractament de les dades personals, us informa que podeu contactar amb el Delegat de Protecció de Dades mitjançant escrit a l'adreça postal (UB: Travessera de les Corts, 131-159, Pavelló Rosa, 08028 – Barcelona; INEFC: Avinguda de l'Estadi 12-22 -08038 Barcelona), o mitjançant un missatge de correu electrònic (UB: protecciodedades@ub.edu; INEFC: Inefc.pd@gencat.cat).La informació referent al seu infant pot ser compartida amb representats de l'INEFC- Barcelona, la Universitat de Barcelona o l'administració governamental si vostè o qualsevol altre es troba en risc o si se'ns és requerit fer-ho per llei.

Riscos

Como a resultats de la seva participació en aquest estudi, el seu infant pot experimental dolor muscular d'aparició tardana ("agulletes" o "tiretes") i fatiga degut a l'esforç físic i muscular realitzat durant les proves de carrera de l'estudi. Així mateix, també pot presentar una lleu sensació de mareig degut a l'esforç realitzat. Donat que també existeix cert risc en situacions de comportament cardíac anormal, el ritme cardíac dels participants serà monitoritzat i controlat durant totes les proves d'exercici. Es definiran un rangs de seguretat i, en cas d'arribar al valor màxim, s'aturarà l'execució de la prova immediatament. No hi ha altres riscos coneguts i no es sap de cap afecte advers a llarg termini que pugui ser associat a la participació d'aquest estudi.

Beneficis, llibertat per retirar-se de l'estudi i per realitzar preguntes

La participació del seu infant és completament voluntària. L'experiment no està dissenyat per ajudar-lo específicament, però pot tenir un impacte substancial per a ajudar-nos a entendre els efectes beneficiosos que pot tenir la realització d'exercici físic sobre la capacitat d'aprenentatge perceptual-motora en nens amb desordres en el desenvolupament de la coordinació. Vostè o el seu infant són lliures de realitzar qualsevol pregunta o, també, de retirar el seu consentiment en la participació de l'estudi en qualsevol moment i sense cap tipus de penalització. Els investigadors li facilitaran un informe amb els resultats d'aquest estudi i les valoracions del seu infant. L'INEFC-Barcelona no li facilitarà cap tipus d'assegurança mèdica o d'hospitalització pel fet de participat en aquest estudi ni tampoc es compensarà econòmicament cap circumstància o contratemps que es pugui donar com a resultat d'aquest estudi, a excepció de que sigui requerit per llei. En cas de qualsevol accident, es recorrerà a l'ús dels dispositius i procediments habituals del centre escolar.

Investigadors Principals

Dr. Albert Busquets Faciabén (Investigador Principal), Dra. Rosa Angulo-Barroso (Investigadora Principal)
Grup de Recerca en Activitat Física, Alimentació i Salut (GRAFAiS),
Institut Nacional d'Educació Fisica de Catalunya, Avinguda de l'Estadi, 12-22, 08038 Barcelona (España)
E-mail del responsable del projecte: albert.busquets@gencat.cat
Telèfon de contacte del responsable del projecte: 934255445 (Ext. 213)

Requeriments del consentiment informat

Vostè ha pres de forma totalment voluntària la decisió de permetre al seu infant a participat o no en l'estudi d'investigació descrit. La seva signatura indica que vostè ha llegit l'informe facilitat anteriorment, que vostè ha pogut rebre resposta a totes les seves preguntes i que decideix deixar participar al seu infant en aquest estudi i autoritza al tractament de les dades de l'infant associades a l'estudi. La signatura del seu infant indica que ell/a ha entès tota la informació rellevant de l'estudi, que s'han respost les possibles preguntes que pogués tenir i que decideix, lliurement, participar en aquest estudi. Se li facilitarà una copia d'aquest document de consentiment informat perquè vostè la guardi.

Informació bàsica sobre protecció de dades

- Tractament : Examining the effects of acute exercise on motor learning and brain activity in children with developmental coordination disorder (ExLe-Brain-DCD)
- Responsables del tractament de dades: Dr. Albert Busquets i Dra. Rosa Angulo (Institut Nacional d'Educació Física de Catalunya)
- Finalitat: dur a terme un estudi sobre la influència de l'exercici físic en la capacitat d'aprenentatge dels infants (amb o sense trastorn del desenvolupament de la coordinació).
- Drets que podeu exercir: Accés, rectificació, limitació, portabilitat, supressió i oposició al tractament. Teniu dret a revocar el consentiment en qualsevol moment sense que tingui efectes retroactius.
- Per a més informació cliqueu al següent link (amb la resta d'informació que esdemana a l'article 13 i 14 RGPD)
    - https://drive.google.com/file/d/1bR14Y_lTE4uCFZWAv560kJFRO-11HRq9/view?usp=sharing

Dades personals de l'infant participant

Nom del participant:____

Codi del participant (a omplir per l'investigador):___________________________

Data de naixement del participant: _____________________________________

Nom del pare, mare o tutor legal del participant:___________________________

____

Telèfon de contacto:____

E-mail:____

Signatura del pare, mare o tutor legal del participant

A ________________________ el ____ de ___________________ de 20__

Dades de l'investigador

Nom de l'investigador:____

Signatura de l'investigador

**Vull revocar el meu consentiment de participació a l'estudi**<br><br>Signatura del pare, mare o tutor legal del participant que vol revocar el consentiment<br><br><br><br>A _________________________ el ____ de ____________________ de 20__

INFORMATION AND INFORMED CONSENT

Institut Nacional d'Educació Física de Catalunya-Barcelona, center of the Universitat de Barcelona

Project identification

Examining the effects of acute exercise on motor learning and brain activity in children with developmental coordination disorder (ExLe-Brain-DCD)

Statement on the age of the participant

You are a person of 18 years or older and the father, mother or legal guardian of this child who is between 7.5 and 10.5 years old. Your child has been invited to participate in a research project led by Dr. Albert Busquets Faciabén and Dr. Rosa Angulo Barroso from the Physical Activity, Nutrition, and Health Research Group of the Institut Nacional d'Educació Física de Catalunya-Barcelona (Barcelona, Spain).

Aim of the study

The main aim of this study is to investigate the influence of performing a single session of intense physical exercise on the learning ability of children with typical motor development (TD) and children with developmental coordination disorder engine (DCD). Specifically, we will study the effect of an exercise session on the learning ability using a hand-eye coordination task that will be executed using a computer. The effects of physical exercise on learning will be studied in the short (1 hour), medium (24 hours) and long term (7 days). To carry out the study, the participants will be divided into 4 groups: group of children with typical development who will exercise (EX-TD), group of children with coordination difficulties who will exercise (EX-DCD), group of children with typical development who will not exercise (CON-TD) and group of children with coordination difficulties who will not exercise (CON-DCD). Your child will participate in one of these groups, children will be randomly assigned to the condition of doing or not exercise prior to the learning task.

Experimental procedure

The study consists of 5 different sessions. If you consent to your child participating in this study, the researchers will administer the following tests:

Prior to the start of the study, candidates' suitability to participate in the study will be assessed through questionnaires on their health habits, medical history, level of weekly physical exercise practice, and on their motor behavior. These questionnaires will be completed by each child's teachers and parents/legal guardians.

Session 1: during the first session of the study, we will administer a test to assess the child's cognitive ability, a test to assess the development of the child's coordination abilities, and we will measure his/her height and weight. In this session, participants will be asked to run a race test in order to assess their level of physical condition. The test will consist of running from one

side to the other of a 20m long track, while following the rhythm set by a sound. Children will be asked to try to make as many paths as possible following the rhythm of the sound. To encourage their effort, this task will be presented as a game. During the test, an elastic band will be placed on the participants' chest that will record the heartbeat frequency (heart rate monitor). This completely innocuous device will be used to assess the participant's level of effort and preserve their safety throughout the test.

Session 2: during the second session, which will take place at least 48 hours after Session 1, the participants will perform three different tasks. On one hand, the participants will be asked to carry out an intense exercise session, which has a very similar development to the race test carried out in the first session. The members of the CON groups (CON-TD and CON-TDC) will not perform this exercise and, instead, will remain at rest doing some quiet activity in the classroom for a time equivalent to the exercise of the other groups (25 min). On the other hand, all participants will also be asked to perform a learning task involving hand-eye coordination, in which participants will control the movements of a circle on a computer screen by using a joystick, with an approach similar to a videogame. The objective of this task will be to move a circle to target points on the screen with the maximum accuracy and speed possible. Participants will be asked to practice this task for approximately 8 minutes. Then, after a one-hour rest period, the participants will be asked to perform the learned task again, this time for only 3.5 minutes in order to check the level of retention of the initial learning (retention test). Before and after performing the exercise or the equivalent rest, a cap will be placed on the head of the participants to measure the activity of the prefrontal brain region, which is closely linked with attention. This device is completely innocuous and measures brain activity through the emission and capture of infrared light, the same as emitted by, for example, a television remote control.

Sessions 3 and 4: during these two sessions, children will again be asked to complete two retention tests of the learned task (one in each session). These two sessions will take place 24 hours and 7 days after the Session 2, respectively. The child will also wear the cap for the measurement of brain activity.

Clothing and glasses needs

In order to do the running tests of sessions 1 and 2, it will be necessary for the participants to attend in comfortable clothes and sports shoes. In addition, in case of presenting a vision problem corrected by wearing glasses, it will be necessary for them to wear the glasses in order to perform the learning task and their retention tests (sessions 2, 3 and 4).

Confidentiality and data protection

All the information collected for this study is strictly confidential. The information collected will come from the tests carried out on your child and from the questionnaires that we will provide. In addition, the behavioral observation of the child that you provide us will be supplemented with information from the teacher responsible for the child's class or another teacher designated by the school, also through a questionnaire. The personal data that we obtain will be grouped with the data of other participants and stored confidentially and

securely in the facilities of the Institut Nacional d'Educació Física de Catalunya (INEFC), Barcelona. Only the principal investigators of this project and their collaborators will have access to the protected personal data files. The data obtained from the tests will be pseudonymized and will be preserved in secure spaces on two protected hard drives and in the data storage cloud. Following the prerogatives of open access science, the data obtained and anonymized may be made public for research purposes and under the same conditions that we have established.

Any video and/or photo images obtained during the course of the study will be anonymized (the face and any other mark that can identify the child will be hidden, pixelated or blurred) and will only be used for the preparation of articles, scientific communications or other forms of dissemination of the results of the study. It is expected that this dissemination of the results will be carried out via scientific journals (online and/or paper publication), scientific presentations (PowerPoint or similar) and dissemination to the general public (web).

The data and images obtained from the research will not be used for any purpose other than the research aims mentioned in this document. They will become part of a data archive for which the principal investigators will have the ultimate responsibility (Dr. Albert Busquets and Dr. Rosa Angulo). Identification data and non-anonymized images will be kept for at least 5 years after the duration of the study and then they will be deleted.

You will be able to exercise at all times the rights established by Organic Law 3/2018, of December 5, on the protection of personal data and guarantee of digital rights and Regulation (EU) 2016/679, of April 27, 2016, of data protection (RGPD). As the legal guardian of the participating child, you have the right to access all his data, request the rectification of inaccurate data or, where appropriate, request its deletion, as well as limit its processing, oppose to use it, and withdraw consent for its use for certain purposes. In addition, both you and your child have the right to withdraw at any time from part or all of the study without stating the cause or reason and without consequences. They also have the right to have any doubts clarified before agreeing to participate, and to know the results of the research.

You can make use of these rights by writing to the postal address or by sending an e-mail message to the address of the principal investigator Dr. Albert Busquets. Likewise, we inform you of your right to file a claim with the Catalan Data Protection Agency in the event of any action that you consider infringing your rights. In addition, in accordance with the provisions of the aforementioned regulation, the University of Barcelona (with CIF Q0818001J and address at Gran Via de les Corts Catalanes, 585 -08007 Barcelona) and INEFC (with CIF Q0840010C and address at Avinguda de l'Estadi 12-22 -08038 Barcelona) as responsible for the processing of personal data, informs you that you can contact the Data Protection Delegate by writing to the postal address (UB: Travessera de les Corts, 131 -159, Pavelló Rosa, 08028 – Barcelona; INEFC: Avinguda de l'Estadi 12-22 -08038 Barcelona), or by email (UB: protecciodedades@ub.edu; INEFC: inefc.pd@gencat.cat). The information regarding your child may be shared with representatives of the INEFC-Barcelona, the University of Barcelona or the government administration if you or anyone else is at risk or if we are required to do so by law.

Risks

As a result of his/her participation in this study, your child may experience delayed onset of muscle soreness ("aches and pains" or "stiffness") and fatigue due to the physical and

muscular effort made during the running tests of the study. Likewise, they may also feel a slight feeling of dizziness due to the effort made. Since there is also a certain risk in situations of abnormal cardiac demands, the heart rate of the participants will be monitored and controlled during all the exercise tests. A safety range will be defined and, if the maximum value is reached, the execution of the test will be stopped immediately. There are no other known risks and no known long-term adverse effects that may be associated with participation in this study.

Benefits, freedom to withdraw from the study and to ask questions

Your child's participation is completely voluntary. The experiment is not designed to help you specifically, but it may have a substantial impact in helping us to understand the beneficial effects that physical exercise may have on perceptual-motor learning ability in children with disorders that affect the development of coordination. You or your child are free to ask any question or, also, to withdraw the consent to participate in the study at any time and without any type of penalty. The researchers will provide you with a report with the results of this study and the evaluations of your child. INEFC-Barcelona will not provide you with any type of medical or hospitalization insurance for participating in this study, nor will any circumstances or setbacks that may occur as a result of this study be financially compensated, with the exception that is required by law. In the event of any accident, the usual firs aid devices and procedures of the school will be used.

Principal Investigators

Dr. Albert Busquets Faciabén (Principal Investigator) and Dra. Rosa Angulo-Barroso (Principal Investigator)
Grup de Recerca en Activitat Física, Alimentació i Salut (GRAFAiS),
Institut Nacional d'Educació Fisica de Catalunya, Avinguda de l'Estadi, 12-22, 08038 Barcelona (España)
E-mail of the project manager: albert.busquets@gencat.cat
Telephone to contact the project manager: 934255445 (Ext. 213)

Informed consent requirements

You have made a completely voluntary decision to allow your child to participate or not in the research study described. Your signature indicates that you have read the report previously provided, that you have been able to receive answers to all your questions and that you decide to let your child participate in this study and authorize the processing of the child's data associated with the study. Your child's signature in the Children's Assent form indicates that he/she has understood all the relevant information about the study, that any possible questions he/she have been answered and that he/she freely decides to participate in this study. You will be provided with a copy of this informed consent document for you to keep.

Basic information on data protection

- Treatment: Examining the effects of acute exercise on motor learning and brain activity in children with developmental coordination disorder (ExLe-Brain-DCD)
- Responsible for data processing: Dr. Albert Busquets and Dr. Rosa Angulo (National Institute of Physical Education of Catalonia)
- Purpose: to carry out a study on the influence of physical exercise on the learning ability of children (with or without developmental coordination disorder).
- Rights you can exercise: Access, rectification, limitation, portability, deletion and opposition to treatment. You have the right to revoke consent at any time without having retroactive effects.
- For more information click on the following link (with the rest of the information required in article 13 and 14 RGPD)
  - https://drive.google.com/file/d/1mtpV65oAfYNSz2KelR3zaKG-ehf4g_f3/view?usp=sharing

Personal data of the participating child

Name of the participant: ____

Code of the participant (to be filled in by the researcher):______________________________

Date of birth of the participant: ____

Name of the participant's father, mother or legal guardian:_____________________________

____

Contact phone number:____

E-mail:____

Signature of the participant's father, mother or legal guardian:

In __________________, ___________________ , 20__

Researcher data

Name of the researcher:____

Signature of the researcher:

**I want to revoke my consent to participate in the study**
Signature of the parent or legal guardian of the participant who wishes to revoke consent
In __________________________, ____________________ , 20__

ANNEX 4 (CHILDREN INFORMED ASSENT)

INFORMACIÓ i ASSENTIMENT INFORMAT

Institut Nacional d'Educació Física de Catalunya-Barcelona, centre adscrit a la Universitat de Barcelona

Identificació del projecte

Examining the effects of acute exercise on motor learning and brain activity in children with developmental coordination disorder (ExLe-Brain-DCD)

Afirmació de l'edat del participant

La teva edat és d'entre 7 anys i mig i 10 anys i mig i estàs pensant en participar en l'estudi que dirigeixen el Dr. Albert Busquets Faciabén i la Dra. Rosa Angulo Barroso del Grup de Recerca en Activitat Física i Salut (GRAFiS, Barcelona, Espanya).

Objectiu

L'objectiu d'aquest estudi és investigar si l'exercici físic intens modifica la manera amb que aprens a fer moviments de les mans per controlar el moviment d'un objecte a la pantalla de l'ordinador. Per aconseguir-ho observarem com aprens una tasca amb l'ordinador i si ets capaç de repetir aquesta mateixa tasca al cap d'1 hora, 24 hores i 7 dies després d'haver-la après. Farem quatre grups de nens i nenes. Dos dels grups faran exercici i els altres dos grups no. Al final de l'estudi compararem l'aprenentatge dels quatre grups.

Procediment experimental

Durant l'estudi hauràs de fer diferents activitats repartides en 5 dies diferents:

*A*bans de poder començar l'estudi, els teus pares hauran d'emplenar uns fulls amb diverses preguntes sobre la teva salut i la teva pràctica d'exercici i activitat física. Si els ajudes, segur que els serà més fàcil emplenar tots els fulls.

Dia 1: durant el primer dia et demanarem que facis una prova d'intel·ligència, on hauràs de fer exercicis mentals; et demanarem que facis un test de coordinació, on hauràs de fer diverses proves com per exemple fer punteria llançant un objecte o fer salts a peu coix; i et mesurarem per saber quant madeixes i quant peses. Durant el mateix dia, farem una prova de carrera. En aquesta prova hauràs d'anar corrent fins a una taula on hi haurà cartes d'un joc de fer parelles i les hauràs de portar a una altra taula, que estarà separada 20 metres. Hauràs d'intentar arribar a cada una de les taules a la vegada que escoltis un pito que sonarà a través d'un altaveu. El pito cada vegada anirà més ràpid, per tant tu també hauràs de córrer cada vegada més de pressa. Perquè la prova surti bé, hauràs d'intentar portar el màxim de cartes possibles d'una taula a l'altre. Abans de començar aquesta prova et col·locarem una cinta al pit que ens servirà per veure la velocitat a la que et batega el cor. Si en qualsevol moment d'aquesta prova no et trobes bé ens ho pots dir i pararem la prova.

Dia 2: el segon dia de proves serà al cap dos dies o més des del segon dia de l'estudi. Aquest dia, en funció del grup en el que et toqui per sorteig, faràs unes proves o unes altres. Si ets dels grups que no fan exercici, començaràs fent un descans de 25 minuts. Durant el descans aprofitarem per pintar, dibuixar, llegir, ... Si ets dels grups que fan exercici, començaràs fent un exercici molt semblant al del segon dia, on hauràs de seguir aconseguint cartes del joc de parelles. Durant l'exercici d'aquest dia aniràs canviant de velocitat, una estona ràpid i una estona lent, sempre seguint el pito. Siguis del grup siguis, després de l'exercici o el descans, et demanarem que aprenguis a controlar un comandament d'ordinador que farà moure un punt verd a la pantalla d'un ordinador. El teu objectiu serà el de moure el punt verd per perseguir un punt vermell que va canviant de posició a la pantalla. Hauràs d'intentar sempre moure el punt verd el més ràpid i recte que puguis, sempre anant a tocar el punt vermell i després tornant al centre de la pantalla. Tindràs més o menys 8 minuts per aconseguir fer-ho el millor que puguis. Després d'una hora d'haver practicat aquesta tasca d'ordinador, et demanarem que la tornis a practicar durant poca estona, més o menys 3 minuts. Al final del segon dia, farem un joc de parelles amb totes les cartes que hagis recollit. Hauràs d'intentar fer el màxim número possible de parelles, per tant, com més cartes recullis abans millor. Durant aquest segon dia, abans i després de fer l'exercici o el descans, et col·locarem una gorro al cap per mesurar l'activació d'una part del teu cervell. Quan et col·loquem el gorro no notaràs res, ja que el gorro mesura l'activitat del cervell a través de llumetes.

Dies 3 i 4: durant aquests dos dies només hauràs de repetir la tasca de l'ordinador. El tercer dia serà l'endemà del dia que hagis practicat amb l'ordinador. El quart dia serà al cap d'una setmana. Aquests dies també et posarem el gorro per mesurar l'activitat cerebral.

Necessitats materials

Per poder fer el millor possible l'exercici de córrer, hauràs de venir amb roba i sabates de fer esport, com si haguessis de fer educació física. Si fas servir ulleres, també serà molt important que les portis per fer la tasca de l'ordinador.

Confidencialitat

Tots els resultats de les proves, les fotos i els vídeos que et fem durant l'estudi, només els podrem veure els investigadors i col·laboradors de l'estudi. Els farem servir per escriure articles i presentar-los a congressos. El teu nom mai sortirà escrit per enlloc, així que ningú sabrà de qui són les puntuacions de les proves ni les persones que surten a les fotos.

Riscos

Després d'haver fet l'exercici físic dels dos primers dies pot ser que et notis una mica cansat, que tinguis agulletes o, fins i tot, que et notis una mica marejat just al acabar de córrer. Si mentre estàs fent l'exercici en algun moment et trobes malament o et mareges, avisa'ns de seguida i pararem les proves de seguida.

Beneficis, llibertat per retirar-se de l’estudi i per realitzar preguntes

Al final de l’estudio, podràs escollir un dels premis que tenim guardats per a tu. Quants més punts aconsegueixis al joc de les parelles del segon dia de l’estudi, millor serà el premi que puguis escollir.

Pots fer-nos qualsevol pregunta que tinguis. Si en algun moment no et trobes bé i no vols seguir participant en l’estudi, pots demanar que et tornem aquest document que tindrà la teva firma i deixar de formar part de l’estudi.

Investigadors Principals

Dra. Rosa Angulo-Barroso

Dr. Albert Busquets Faciabén

Grup de Recerca en Activitat Física i Salut, Avinguda de l’Estadi, 12-22

08038 Barcelona (España)

Requeriment de l'assentiment informat

La teva signatura en aquest document significa que vols participar en l'estudi, que has entès totes les proves en les que et demanarem que participis i que t'hem contestat totes les preguntes que tenies. Et donarem una copia d'aquest document perquè la guardis.

Dades personals de l'infant participant

Nom i cognoms del participant:____

Codi del participant (a omplir per l'investigador):____

Signatura del participant

A _________________________ el ____ de ____________________ de 20__

Dades de l'investigador

Nom de l'investigador:____

Signatura de l'investigador

**Vull revocar el meu consentiment de participació a l'estudi**
Signatura del participant que vol revocar el consentiment
A _________________________ el ____ de ____________________ de 20__

Informed consent 2 – for children

INFORMATION and INFORMED CONSENT

Institut Nacional d'Educació Física de Catalunya-Barcelona, centre of the Universitat de Barcelona

Project identification

Examining the effects of acute exercise on motor learning and brain activity in children with developmental coordination disorder (ExLe-Brain-DCD)

Affirmation of the age of the participant

Your age is between 7 and a half and 10 and a half years and you are thinking of participating in the study led by Dr. Albert Busquets Faciabén and Dr. Rosa Angulo Barroso from the Physical Activity, Nutrition, and Health Research Group (GRAFAiS, Barcelona, Spain).

Aim

The aim of this study is to investigate whether intense physical exercise changes the way you learn to make hand movements to control the movement of an object on the computer screen. To achieve this objective, we will observe how you learn this task with the computer and if you are able to repeat this same task after 1 hour, 24 hours and 7 days after learning it. We will make four groups of boys and girls. Two of the groups will exercise and the other two groups will not. At the end of the study we will compare the learning of the four groups.

Experimental procedure

During the study you will have to do different activities spread over 5 different days:

Before you can start the study, your parents will have to fill in some sheets with several questions about your health and your practice of exercise and physical activity. If you help them, it will surely be easier for them to fill in all the sheets.

Day 1: during the first day we will ask you to take an intelligence test, where you will have to do mental exercises; we will ask you to take a coordination test, where you will have to do several tests such as aiming by throwing an object or jumping with a limp; and we'll measure you to find out how tall you are and how much you weigh. During the same day, we will do a race test. In this test you will have to run to a table where there will be cards from a matching card game and you will have to take them to another table, which will be 20 meters apart. You will have to try to reach each of the tables while listening to a whistle that will sound through a speaker. The whistle will go faster and faster, so you will also have to run faster and faster. For the test to go well, you will have to try to bring as many cards as possible from one table to the other. Before starting this test we will place a tape on your chest that will be used to see the speed at which your heart beats. If at any time during this test you do not feel well, you can tell us and we will stop the test.

Day 2: The second day of testing will be two days or more after the second day of study. On this day, depending on the group in which you are drawn by lottery, you will do some tests or others. If you're in the non-exercising group, you'll start with a 25-minute break. During the break we will take the opportunity to paint, draw, read, ... If you are one of the groups that exercise, you will start by doing an exercise very similar to the one on the second day, where you will have to keep getting cards from the card matching game. During this day's exercise you will be changing speed, one time fast and one time slow, always following the whistle. After the exercise or the break, we will ask you to learn how to control a computer joystick that will move a green dot on a computer screen. Your goal will be to move the green dot to chase a red dot that changes position on the screen. You should always try to move the green dot as fast and as straight as you can, always touching the red dot and then returning to the center of the screen. You will have more or less 8 minutes to manage to do it the best you can. After an hour of finishing your first practice with this computer task, we will ask you to practice it again for a short time, more or less 3 minutes. At the end of the second day, we will play a matching game with all the cards you have collected. You will have to try to make as many pairs as possible, so the more cards you collect the better. During this second day, before and after exercising or resting, we will place a cap on your head to measure the activation of the front part of your brain. When we put the cap on you, you won't notice anything, as the cap measures brain activity through lights.

Days 3 and 4: during these two days you will only have to repeat the computer task. The third day will be the day after the day you practiced with the computer. The fourth day will be one week later. These days we will also put the cap on you to measure brain activity.

Clothing and glasses needs

To be able to do your best during the running exercise, you will have to come in sports clothes and shoes, as if you had to do physical education. If you wear glasses, it will also be very important that you wear them to do the computer work.

Confidentiality

All the test results, photos and videos we take of you during the study can only be seen by the researchers and collaborators of the study. We will use them to write articles and present them at conferences. Your name will never be written anywhere, so no one will know whose test scores are whose or who is in the photos.

Risks

After you've done the physical exercise of the first two days, you may feel a little tired, have pain and needles sensations in your muscles or even feel a little dizzy right after you finish running. If while you are doing the exercise at any point you feel sick or dizzy, let us know immediately and we will stop the tests immediately.

Benefits, freedom to withdraw from the study and to ask questions

At the end of the study, you can choose one of the prizes we have saved for you. The more points you get in the pairs game on the second day of the study, the better the prize you can choose.

You can ask us any question you may have. If at any time you do not feel well and do not want to continue participating in the study, you can ask us to return this document with your signature and stop being part of the study.

Principal Investigators

Dra. Rosa Angulo-Barroso

Dr. Albert Busquets Faciabén

Grup de Recerca en Activitat Física i Salut, Avinguda de l'Estadi, 12-22

08038 Barcelona (España)

Requirement of informed consent

Your signature on this document means that you want to participate in the study, that you have understood all the tests in which we will ask you to participate and that we have answered all the questions you had. We will give you a copy of this document to keep.

Personal data of the participating child

Name and surname of the participant:____

Participant code (to be filled in by the researcher):____________________________________

Signature of the participant

In _________________________ , ____________________ , 20__

Data of the researcher

Name of the researcher:____

Signature of the researcher

**I want to revoke my consent to participate in the study**
Signature of the participant who wishes to revoke consent
In_________________________ , ____________________ , 20__

ANNEX 5 (QUESTIONNAIRES)

QÜESTIONARI D'APTITUD PER LA PARTICIPACIÓ EN L'ESTUDI

INFORMACIÓ PERSONAL DEL PARTICIPANT, ESTAT SOCIOECONÒMIC FAMILIAR, ESTAT DE SALUT, HÀBITS DE SALUT I AVALUACIÓ DE LA CONDUCTA MOTRIU

*Els següents qüestionaris es troben redactats en llengua castellana, donat que és l'idioma en el qual han estat validats pel seu ús científic.

INFORMACIÓ PERSONAL

**Nom de l'infant:**
**Cognoms de l'infant:**
**Data de naixement de l'infant:**
**Altres comentaris** (si creu que és necessari fer-nos coneixedors d'alguna informació que no es vegi reflectida en les preguntes d'aquest qüestionari):

Código Participante

CUESTIONARIO SOBRE EL ESTADO SOCIOECONÓMICO FAMILIAR

Preguntas sobre la vivienda

Cuantas personas residen en la vivienda habitual del niño/a: ________

Marque la opción más cierta en su caso en cuanto a la composición familiar:

- ◯ Familia monoparental
- ◯ Familia biparental (familia residente en una única vivienda)
- ◯ Familia biparental (padres/tutores legales separados)
- ◯ Otras situaciones familiares, especificar: ____

A continuación, responda las siguientes preguntas para cada una de las personas mayores de 16 años que residan en la misma vivienda que el niño/a participante en el estudio:

Persona 1

¿Cuál es su nivel máximo de estudios finalizados? (marque la opción más cierta para esta persona)

- ◯ No sabe leer ni escribir
- ◯ Estudios primarios incompletos (sabe leer y escribir sin haber finalizado la educación primaria)
- ◯ Estudios primarios completos (cinco cursos aprobados de EGB)
- ◯ Primera etapa de educación secundaria (graduado escolar, bachillerato elemental, EGB o ESO)
- ◯ Enseñanzas de bachillerato (bachillerato superior, BUP, bachillerato plan nuevo, PREU o COU)
- ◯ Formación profesional de grado medio (oficialía indistrial, FP I, ciclos formativos de grado medio)
- ◯ Formación profesional de grado superior (maestría industrial, FP II, ciclos formativos de grado superior)
- ◯ Estudios universitarios de grado medio (diplomaturas y enseñanzas universitarias de primer ciclo)
- ◯ Estudios universitarios de grado superior (licenciaturas y enseñanzas universitarias de segundo ciclo o máster)
- ◯ Estudios universitarios de tercer ciclo (doctorado)
- ◯ Otra posibilidad, especificar:____

Situación laboral principal en la actualidad (marque la opción más cierta para esta persona):

- ◯ Asalariado
- ◯ En paro con prestación económica
- ◯ En paro sin prestación económica
- ◯ Jubilado/da (excluir jubilaciones parciales)
- ◯ Estudiante
- ◯ Autónomo
- ◯ Otra situación, especificar: ____

Sueldo mensual percibido a través de la actividad profesional:
- ◯ Menos de 710,1€
- ◯ Entre 710,1€ y 1.002,7€
- ◯ Entre 1.002,8€ y 1.229,2
- ◯ Entre 1.229,3€ y 1.412,6€
- ◯ Entre 1.412,7€ y 1.594,4€
- ◯ Entre 1.594,5€ y 1.824,0€
- ◯ Entre 1.824,1€ y 2.137,4€
- ◯ Entre 2.137,5€ y 2.595,0€
- ◯ Entre 2.595,1€ y 3.316,8€
- ◯ 3.316,9€ o más euros

Persona 2

¿Cuál es su nivel máximo de estudios finalizados? (marque la opción más cierta para esta persona)
- ◯ No sabe leer ni escribir
- ◯ Estudios primarios incompletos (sabe leer y escribir sin haber finalizado la educación primaria)
- ◯ Estudios primarios completos (cinco cursos aprobados de EGB)
- ◯ Primera etapa de educación secundaria (graduado escolar, bachillerato elemental, EGB o ESO)
- ◯ Enseñanzas de bachillerato (bachillerato superior, BUP, bachillerato plan nuevo, PREU o COU)
- ◯ Formación profesional de grado medio (oficialía indistrial, FP I, ciclos formativos de grado medio)
- ◯ Formación profesional de grado superior (maestría industrial, FP II, ciclos formativos de grado superior)
- ◯ Estudios universitarios de grado medio (diplomaturas y enseñanzas universitarias de primer ciclo)
- ◯ Estudios universitarios de grado superior (licenciaturas y enseñanzas universitarias de segundo ciclo o máster)
- ◯ Estudios universitarios de tercer ciclo (doctorado)
- ◯ Otra posibilidad, especificar:____

Situación laboral principal en la actualidad (marque la opción más cierta para esta persona):
- ◯ Asalariado
- ◯ En paro con prestación económica
- ◯ En paro sin prestación económica
- ◯ Jubilado/da (excluir jubilaciones parciales)
- ◯ Estudiante
- ◯ Autónomo
- ◯ Otra situación, especificar: ____

Sueldo mensual percibido a través de la actividad profesional:
- ◯ Menos de 710,1€
- ◯ Entre 710,1€ y 1.002,7€
- ◯ Entre 1.002,8€ y 1.229,2
- ◯ Entre 1.229,3€ y 1.412,6€
- ◯ Entre 1.412,7€ y 1.594,4€

- ◯ Entre 1.594,5€ y 1.824,0€
- ◯ Entre 1.824,1€ y 2.137,4€
- ◯ Entre 2.137,5€ y 2.595,0€
- ◯ Entre 2.595,1€ y 3.316,8€
- ◯ 3.316,9€ o más euros

Persona 3

¿Cuál es su nivel máximo de estudios finalizados? (marque la opción más cierta para esta persona)

- ◯ No sabe leer ni escribir
- ◯ Estudios primarios incompletos (sabe leer y escribir sin haber finalizado la educación primaria)
- ◯ Estudios primarios completos (cinco cursos aprobados de EGB)
- ◯ Primera etapa de educación secundaria (graduado escolar, bachillerato elemental, EGB o ESO)
- ◯ Enseñanzas de bachillerato (bachillerato superior, BUP, bachillerato plan nuevo, PREU o COU)
- ◯ Formación profesional de grado medio (oficialía indistrial, FP I, ciclos formativos de grado medio)
- ◯ Formación profesional de grado superior (maestría industrial, FP II, ciclos formativos de grado superior)
- ◯ Estudios universitarios de grado medio (diplomaturas y enseñanzas universitarias de primer ciclo)
- ◯ Estudios universitarios de grado superior (licenciaturas y enseñanzas universitarias de segundo ciclo o máster)
- ◯ Estudios universitarios de tercer ciclo (doctorado)
- ◯ Otra posibilidad, especificar:____

Situación laboral principal en la actualidad (marque la opción más cierta para esta persona):

- ◯ Asalariado
- ◯ En paro con prestación económica
- ◯ En paro sin prestación económica
- ◯ Jubilado/da (excluir jubilaciones parciales)
- ◯ Estudiante
- ◯ Autónomo
- ◯ Otra situación, especificar: ____

Sueldo mensual percibido a través de la actividad profesional:

- ◯ Menos de 710,1€
- ◯ Entre 710,1€ y 1.002,7€
- ◯ Entre 1.002,8€ y 1.229,2
- ◯ Entre 1.229,3€ y 1.412,6€
- ◯ Entre 1.412,7€ y 1.594,4€
- ◯ Entre 1.594,5€ y 1.824,0€
- ◯ Entre 1.824,1€ y 2.137,4€
- ◯ Entre 2.137,5€ y 2.595,0€
- ◯ Entre 2.595,1€ y 3.316,8€
- ◯ 3.316,9€ o más euros

Persona 4

¿Cuál es su nivel máximo de estudios finalizados? (marque la opción más cierta para esta persona)

- ◯ No sabe leer ni escribir
- ◯ Estudios primarios incompletos (sabe leer y escribir sin haber finalizado la educación primaria)
- ◯ Estudios primarios completos (cinco cursos aprobados de EGB)
- ◯ Primera etapa de educación secundaria (graduado escolar, bachillerato elemental, EGB o ESO)
- ◯ Enseñanzas de bachillerato (bachillerato superior, BUP, bachillerato plan nuevo, PREU o COU)
- ◯ Formación profesional de grado medio (oficialía indistrial, FP I, ciclos formativos de grado medio)
- ◯ Formación profesional de grado superior (maestría industrial, FP II, ciclos formativos de grado superior)
- ◯ Estudios universitarios de grado medio (diplomaturas y enseñanzas universitarias de primer ciclo)
- ◯ Estudios universitarios de grado superior (licenciaturas y enseñanzas universitarias de segundo ciclo o máster)
- ◯ Estudios universitarios de tercer ciclo (doctorado)
- ◯ Otra posibilidad, especificar:____

Situación laboral principal en la actualidad (marque la opción más cierta para esta persona):

- ◯ Asalariado
- ◯ En paro con prestación económica
- ◯ En paro sin prestación económica
- ◯ Jubilado/da (excluir jubilaciones parciales)
- ◯ Estudiante
- ◯ Autónomo
- ◯ Otra situación, especificar: ____

Sueldo mensual percibido a través de la actividad profesional:

- ◯ Menos de 710,1€
- ◯ Entre 710,1€ y 1.002,7€
- ◯ Entre 1.002,8€ y 1.229,2
- ◯ Entre 1.229,3€ y 1.412,6€
- ◯ Entre 1.412,7€ y 1.594,4€
- ◯ Entre 1.594,5€ y 1.824,0€
- ◯ Entre 1.824,1€ y 2.137,4€
- ◯ Entre 2.137,5€ y 2.595,0€
- ◯ Entre 2.595,1€ y 3.316,8€
- ◯ 3.316,9€ o más euros

CUESTIONARIO SOBRE EL ESTADO DE SALUD: PREGUNTES SOBRE ANTECEDENTS MÈDICS		
	SI	NO
¿Utiliza su niño/a gafas para poder leer o trabajar con el ordenador?		
¿Padece su niño/a algún trastorno psíquico/psiquiátrico que pueda interferir en su capacidad de aprendizaje?(p.e. Trastorno de déficit de atención, hiperactividad, dislexia, …)<br><br>En caso de responder SI, indique cual es el trastorno y el año de diagnóstico del mismo:		
¿Está su niño/a tomando medicamentos recetados por el médico/psiquiatra de forma regular?<br><br>En caso de responder SI, indique la siguiente información:<br>• Motivo de la receta:<br>• Medicamento recetado:<br>• Cantidad y frecuencia de toma de la medicación:		
¿Existen antecedentes familiares del "Trastorno del Desarrollo de la Coordinación"?<br><br>En caso de responder si, explicar el diagnóstico y proximidad del familiar:		

¿Existen antecedentes familiares de algún trastorno psicológico o psiquiátrico?<br><br>En caso de responder si, explicar el diagnóstico y proximidad del familiar:		

CUESTIONARIO SOBRE EL ESTADO DE SALUD:<br>PHYSICAL ACTIVITY READINESS QUESTIONNAIRE		
	SI	NO
¿Algún médico le ha dicho que su niño/a tiene problemas del corazón y que sólo debe hacer actividades físicas recomendadas por un médico?		
¿Tiene su niño/a dolor en el pecho cuando hace alguna actividad física?		
¿Pierde su niño/a el equilibro por mareos, o ha perdido alguna vez el conocimiento?		
¿Tiene su niño/a problemas en algún hueso o articulación que pueda ser agravado por un cambio en su actividad física?		
¿Padece su niño/a asma no controlado (p.e. asma que no es fácilmente controlable con el uso de un inhalador)?		
¿Está su niño/a tomando medicamentos recetados por el médico para la presión arterial o para el corazón (por ejemplo, pastillas diuréticas)?		
¿Sabe de cualquier otra razón en contra de que su niño/a realice ejercicio físico? (Incluyendo diabetes, una lesión o enfermedad grave reciente)		

CUESTIONARIO DE ACTIVIDAD FISICA PARA NIÑOS (PAQ-C)

Queremos conocer cuál es tu nivel de actividad física en los últimos 7 días (última semana). Esto incluye todas aquellas actividades como deportes, gimnasia o danza que hacen sudar o sentirte cansado, o juegos que hagan que se acelere tu respiración como jugar al pilla-pilla, saltar a la comba, correr, trepar y otras.

Recuerda:

1- No hay respuestas buenas o malas. Esto NO es un examen.
2- Contesta las preguntas de la forma más honesta y sincera posible. Esto es muy importante.

---

1. Actividad Física en tu tiempo libre: ¿Has hecho alguna de estas actividades en los últimos 7 días (última semana)? Si tu respuesta es sí: ¿Cuántas veces lo has hecho? (*Marca un solo círculo por actividad*)

	No	1-2	3-4	5-6	7 veces o +
Saltar a la comba					
Patinar					
Jugar a juegos como el pilla-pilla					
Montar en bicicleta					
Caminar (como ejercicio)					
Correr/footing					
Gimnasia artística					
Gimnasia rítmica					
Natación					
Bailar/danza					
Bádminton					
Rugby					
Montar en monopatín					
Fútbol/fútbol sala					
Voleibol					
Hockey					
Baloncesto					
Esquiar					
Otros deportes de raqueta					
Balonmano					
Atletismo					
Artes marciales (judo, Kárate, …)					
Otros:____________________________					
Otros:____________________________					

2. En los últimos 7 días, durante las clases de educación física, ¿cuántas veces estuviste muy activo durante las clases (jugando intensamente, corriendo, saltando, haciendo lanzamientos)? (Señala sólo una)

No hice/hago educación física	
Casi nunca	
Algunas veces	
A menudo	
Siempre	

3. En los últimos 7 días ¿Qué hiciste normalmente durante el recreo? (Señala sólo una)

Estar sentado (hablar, leer, trabajo de clase)	
Estar o pasear por los alrededores	
Correr o jugar un poco	
Correr y jugar bastante	
Correr y jugar intensamente todo el tiempo	

4. En los últimos 7 días ¿Qué hiciste normalmente a la hora de la comida (antes y después de comer)? (Señala sólo una)

Estar sentado (hablar, leer, trabajo de clase)	
Estar o pasear por los alrededores	
Correr o jugar un poco	
Correr y jugar bastante	
Correr y jugar intensamente todo el tiempo	

5. En los últimos 7 días, inmediatamente después de la escuela hasta las 6, ¿cuántos días jugaste a algún juego hiciste deporte o bailes en los que estuviste muy activo? (Señala sólo una)

Ninguna	
1 vez en la última semana	
2-3 veces en la última semana	
4 veces en la última semana	
5 veces en la última semana	

6. En los últimos 7 días, cuantos días a partir de media tarde (entre las 6 y las 10) hiciste deportes, baile o jugaste a juegos en los que estuvieras muy activo? (Señala sólo una)

Ninguna	
1 vez en la última semana	
2-3 veces en la última semana	
4-5 veces en la última semana	
6-7 veces en la última semana	

7. El último fin de semana, ¿cuántas veces hiciste deportes, baile o jugar a juegos en los que estuviste muy activo? (Señala sólo una)

Ninguna	
1 vez	
2-3 veces	
4-5 veces	
6 veces	

8. ¿Cuáles de las siguientes frases describen mejor tu última semana? Lee las cinco antes de decidir cuál te describe mejor. (Señala sólo una)

Todo o la mayoría de mi tiempo libre lo dediqué a actividades que suponen poco esfuerzo físico	
Algunas veces (1-2 veces a la semana) hice actividades físicas en mi tiempo libre (por ejemplo, hacer deportes, correr, nadar, montar en bicicleta, hacer aeróbic)	
A menudo (3-4 veces a la semana) hice actividad física en mi tiempo libre	
Bastante a menudo (5-6 veces en la última semana) hice actividad física en mi tiempo libre	
Muy a menudo (7 o más veces en la última semana) hice actividad física en mi tiempo libre	

9. Señala con qué frecuencia hiciste actividad física para cada día de la semana (como hacer deporte, jugar, bailar o cualquier otra actividad física) (Poca= entre 20 y 30 minutos, Normal= entre 30 minutos y 1 hora, Bastante= entre 1 hora y 2 horas, Mucha= más de 2 horas).

	Ninguna	Poca	Normal	Bastante	Mucha
Lunes					
Martes					
Miércoles					
Jueves					
Viernes					
Sábado					
Domingo					

10. ¿Estuviste enfermo esta última semana o algo impidió que hicieras normalmente actividades físicas?

Si	
No	

En caso que tu respuesta sea “Si”, que fue lo que te pasó?_________________________

LISTA DE OBSERVACIÓN CONDUCTUAL DE LA BATERÍA DE EVALUACIÓN DEL MOVIMIENTO PARA NIÑOS -2

Complete las secciones A y B antes de completar la valoración global del cuadro final de la siguiente página.

Sección A. Moverse en un entorno estàtico y/o predecible

0= Muy bien 1= Bien 2= Regular 3= Con mucha dificultad NO= No observado	
**A.1 *Autonomía personal***	
A.1.1 - Mantiene el equilibrio mientras permanece de pie poniéndose la ropa (p. ej., pantalones, falda, jersey, etc.).	
A.1.2 - Se pone la ropa por la cabeza (p. ej., camisetas, jerseys).	
A.1.3 - Se abrocha los botones (p. ej., camisa, chaqueta).	
A.1.4 – Se lava y seca las manos	
A.1.5 – Vierte líquidos de un recipiente a otro (p. ej., de una jarra a un vaso).	
**A.2 *Habilidades en el aula***	
A.2.1 – Manipula objetos pequeños (p. ej., fichas, cuentas de collar, hojas de papel).	
A.2.2 – Escribe letras usando lápiz o bolígrafo	
A.2.3 – Usa tijeras para cortar papel.	
A.2.4 – Camina por la clase evitando los objetos o personas inmóviles	
A.2.5 – Transporta objetos (p. ej., libros, botes de lápices) de un lugar a otro sin que se le caigan.	
**A.3 *Habilidades deportivas y recreativas***	
A.3.1 – Salta manteniendo los dos pies juntos al impulsarse y al aterrizar en el suelo.	
A.3.2 – Salta a la pata coja con cualquiera de sus pies.	
A.3.3 – Lanza un saquito o una pelota para que otro niño que está quieto pueda cogerlas.	
A.3.4 – Usa el equipamiento fijo del gimnasio/parque infantil (p. ej., espalderas, toboganes).	
A.3.5 – Cruza el gimnasio/parque infantil evitando chocar con objetos o personas inmóviles.	
**SECCIÓN A: TOTAL**	

Información adicional (opcional)

Por favor, indique si el niño tiene dificultades de aprendizaje ☐

y/o dificultades en: atención ☐ habla ☐ lectura ☐

relaciones sociales ☐ control emocional ☐

Sección B. Moverse en un entorno dinámico y/o impredecible

0= Muy bien 1= Bien 2= Regular 3= Con mucha dificultad NO= No observado	
B.1 Autonomía personal/Habilidades en el aula	
B.1.1 – Mantiene el equilibro cuando se requieren ajustes posturales frecuentes (p. ej., sentarse en un banco al tiempo que lo hacen otros niños, permanecer en una fila con otros niños en movimiento).	
B.1.2 – Se mueve en una clase repleta recogiendo o repartiendo objetos (p. ej., libros, lápices).	
B.1.3 – Lleva una bandeja o bebida de un lugar a otro evitando personas en movimientos (p. ej., en el comedor)	
B.1.4 – Lleva el compás de la música dando palmas o golpecitos con el pie en el suelo	
B.1.5 – Mueve el cuerpo al ritmo de la música o al ritmo de otras personas (p. ej., baile y actividades rítmicas en grupo).	
B.2 Habilidades con balón o pelota	
B.2.1 – Atrapa un balón usando las dos manos	
B.2.2 – Golpea una pelota en movimiento usando una raqueta	
B.2.3 – Lanza un balón mientras está en movimiento para que otro niño lo pueda atrapar.	
B.2.4 – Bota repetidamente un balón de forma controlada.	
B.2.5 – Participa en juegos colectivos empleando habilidades de lanzamiento, atrape, chute o golpeo de balón.	
B.3 Habilidades deportivas y recreativas	
B.3.1 – Monta en bicicleta sin <<Ruedines>>.	
B.3.2 – Participa en juegos de persecución y pillar (p. ej., pilla-pilla)	
B.3.3 – Mantiene el equilibrio en el agua estando con otros niños (p. ej., en la piscina)	
B.3.4 – Usa los materiales móviles del gimnasio/parque infantil (p. ej., aros, patinetes).	
B.3.5 – Cruza el gimnasio/parque infantil evitando chocar con objetos o personas en movimiento.	
**SECCIÓN B: TOTAL**	

Evaluación global

¿De forma general, cree que este niño presenta dificultades de movimiento? **Si / No**

En caso afirmativo, ¿estas dificultades afecta negativamente al niño? (rodear con un círuclo)

**Aprendizajes escolares**	En nada	Un poco	Mucho
**Actividades recreativas**	En nada	Un poco	Mucho
**Autoestima**	En nada	Un poco	Mucho
**Interacciones sociales**	En nada	Un poco	Mucho

Sección C. Factores no motores que pueden afectar al movimiento

	Si	No
C.1 – **Desorganizado** (p.ej., se pone los zapatos antes que los calcetines		
C.2 – **Indeciso/Despistado** (p. ej., tarda en iniciar acciones complejas; olvida lo que tiene que hacer en la mitad de la tarea)		
C.3 – **Pasivo** (p. ej., sin interés; necesita mucho estímulo para participar).		
C.4 – **Temeroso** (p. ej., miedoso ante actividades que requieren saltos o escalada; solicita constantemente ayuda).		
C.5 – **Ansioso** (p. ej., tiembla; se aturde en situaciones estresantes)		
C.6 – **Impulsivo** (p. ej., empieza antes de terminar de escuchar las instrucciones; impaciente).		
C.7 . **Distraído** (p. je., mira a su alrededor; responde a ruidos irrelevantes).		
C.8 – **Hiperactivo** (p. ej., se agita y retuerce; se mueve constantemente cuando escucha instrucciones; juguetea con la ropa)		
C.9 – **Sobreestima sus capacidades** (p. ej., intenta hacer tareas más difíciles; intenta hacer las cosas demasiado rápido).		
C.10 – **Subestima sus capacidades** (p. ej., se queja que la tarea es difícil; anticipa el fallo antes de empezar)		
C.11 – **Falta de persistencia** (p. ej., abandona rápidamente; se frustra fácilmente)		
C.12 – **Enfado ante el fallo** (p. ej., parece lloroso; declina intentar otra vez la tarea)		
C.13 – **Incapaz de obtener satisfacción del éxito** (p. ej., no responde a los elogios).		
Otros (especificar):		

Factores no motores que pueden afectar al movimiento

(a) ¿Piensa usted que los factores no motores indicados en la sección C de la Lista de Observación Conductual impide que el niño demuestre su capacidad real de movimiento?: **en nada / un poco / mucho**

(b) ¿Cómo cree que influirán estas dificultades cuando se planifique el programa de intervención?: **en nada / moderadamente / mucho**

SUITABILITY QUESTIONNAIRE FOR PARTICIPATION IN THE STUDY

PERSONAL INFORMATION OF THE PARTICIPANT, FAMILY SOCIO-ECONOMIC STATUS, HEALTH STATUS, HEALTH HABITS AND ASSESSMENT OF MOTOR BEHAVIOR

* The following questionnaires are written in Spanish, given that it is the language in which they have been validated for their scientific use.

PERSONAL INFORMATION

**Child's name:**
**Child's Surname:**
**Child's Date of birth:**
**Other comments** (if you think it is necessary to make us aware of any information that is not reflected in the questions of this questionnaire):

Participant's ID

QUESTIONNAIRE ON FAMILY SOCIOECONOMIC STATUS

Questions about housing

How many people live in the child's usual home: ________

Mark the most accurate option in your case regarding family composition:

- ◯ Single parent family
- ◯ Two-parent family living together (family living in a single dwelling)
- ◯ Two-parent family living separated (separated parents/legal guardians)
- ◯ Other family situations, specify: ____

<u>Next, answer the following questions for each person older than 16 who lives in the same home as the child participating in the study:</u>

<u>Person 1</u>

What is your maximum level of completed studies? (mark the most accurate option for this person)

- ◯ Can't read or write
- ◯ Incomplete primary education (can read and write without finishing primary education)
- ◯ Complete primary studies (five completed and passed EGB courses)
- ◯ First stage of secondary education (school graduation, elementary baccalaureate, EGB or ESO)
- ◯ Baccalaureate education (upper baccalaureate, BUP, new plan baccalaureate, PRICE or COU)
- ◯ Secondary vocational training (industrial operator, FP I, secondary training courses)
- ◯ Advanced professional training (industrial master's degree, FP II, advanced training courses)
- ◯ Intermediate university studies (diplomas and first cycle university courses)
- ◯ Higher degree university studies (degrees and second-cycle university courses or master's)
- ◯ Third cycle university studies (doctorate)
- ◯ Other possibility, specify:____

Current main employment situation (mark the most accurate option for this person):

- ◯ Salaried
- ◯ Unemployment with economic benefits
- ◯ Unemployment without financial benefits
- ◯ Retired (excluding partial retirements)
- ◯ Student
- ◯ Autonomous
- ◯ Other situation, specify: ____

Monthly salary received through professional activity:

- ◯ Less than 710,1€
- ◯ Between 710,1€ and 1.002,7€
- ◯ Between 1.002,8€ and 1.229,2

- ◯ Between 1.229,3€ and 1.412,6€
- ◯ Between 1.412,7€ and 1.594,4€
- ◯ Between 1.594,5€ and 1.824,0€
- ◯ Between 1.824,1€ and 2.137,4€
- ◯ Between 2.137,5€ and 2.595,0€
- ◯ Between 2.595,1€ and 3.316,8€
- ◯ 3.316,9€ or more euros

Person 2

What is your maximum level of completed studies? (mark the most accurate option for this person)

- ◯ Can't read or write
- ◯ Incomplete primary education (can read and write without finishing primary education)
- ◯ Complete primary studies (five completed and passed EGB courses)
- ◯ First stage of secondary education (school graduation, elementary baccalaureate, EGB or ESO)
- ◯ Baccalaureate education (upper baccalaureate, BUP, new plan baccalaureate, PRICE or COU)
- ◯ Secondary vocational training (industrial operator, FP I, secondary training courses)
- ◯ Advanced professional training (industrial master's degree, FP II, advanced training courses)
- ◯ Intermediate university studies (diplomas and first cycle university courses)
- ◯ Higher degree university studies (degrees and second-cycle university courses or master's)
- ◯ Third cycle university studies (doctorate)
- ◯ Other possibility, specify:____

Current main employment situation (mark the most accurate option for this person):

- ◯ Salaried
- ◯ Unemployment with economic benefits
- ◯ Unemployment without financial benefits
- ◯ Retired (excluding partial retirements)
- ◯ Student
- ◯ Autonomous
- ◯ Other situation, specify: ____

Monthly salary received through professional activity:

- ◯ Less than 710,1€
- ◯ Between 710,1€ and 1.002,7€
- ◯ Between 1.002,8€ and 1.229,2
- ◯ Between 1.229,3€ and 1.412,6€
- ◯ Between 1.412,7€ and 1.594,4€
- ◯ Between 1.594,5€ and 1.824,0€
- ◯ Between 1.824,1€ and 2.137,4€
- ◯ Between 2.137,5€ and 2.595,0€
- ◯ Between 2.595,1€ and 3.316,8€
- ◯ 3.316,9€ or more euros

Person 3

What is your maximum level of completed studies? (mark the most accurate option for this person)

- ◯ Can't read or write
- ◯ Incomplete primary education (can read and write without finishing primary education)
- ◯ Complete primary studies (five completed and passed EGB courses)
- ◯ First stage of secondary education (school graduation, elementary baccalaureate, EGB or ESO)
- ◯ Baccalaureate education (upper baccalaureate, BUP, new plan baccalaureate, PRICE or COU)
- ◯ Secondary vocational training (industrial operator, FP I, secondary training courses)
- ◯ Advanced professional training (industrial master's degree, FP II, advanced training courses)
- ◯ Intermediate university studies (diplomas and first cycle university courses)
- ◯ Higher degree university studies (degrees and second-cycle university courses or master's)
- ◯ Third cycle university studies (doctorate)
- ◯ Other possibility, specify:____

Current main employment situation (mark the most accurate option for this person):

- ◯ Salaried
- ◯ Unemployment with economic benefits
- ◯ Unemployment without financial benefits
- ◯ Retired (excluding partial retirements)
- ◯ Student
- ◯ Autonomous
- ◯ Other situation, specify: ____

Monthly salary received through professional activity:

- ◯ Less than 710,1€
- ◯ Between 710,1€ and 1.002,7€
- ◯ Between 1.002,8€ and 1.229,2
- ◯ Between 1.229,3€ and 1.412,6€
- ◯ Between 1.412,7€ and 1.594,4€
- ◯ Between 1.594,5€ and 1.824,0€
- ◯ Between 1.824,1€ and 2.137,4€
- ◯ Between 2.137,5€ and 2.595,0€
- ◯ Between 2.595,1€ and 3.316,8€
- ◯ 3.316,9€ or more euros

Person 4

What is your maximum level of completed studies? (mark the most accurate option for this person)

- ◯ Can't read or write
- ◯ Incomplete primary education (can read and write without finishing primary education)

- ◯ Complete primary studies (five completed and passed EGB courses)
- ◯ First stage of secondary education (school graduation, elementary baccalaureate, EGB or ESO)
- ◯ Baccalaureate education (upper baccalaureate, BUP, new plan baccalaureate, PRICE or COU)
- ◯ Secondary vocational training (industrial operator, FP I, secondary training courses)
- ◯ Advanced professional training (industrial master's degree, FP II, advanced training courses)
- ◯ Intermediate university studies (diplomas and first cycle university courses)
- ◯ Higher degree university studies (degrees and second-cycle university courses or master's)
- ◯ Third cycle university studies (doctorate)
- ◯ Other possibility, specify:____

Current main employment situation (mark the most accurate option for this person):

- ◯ Salaried
- ◯ Unemployment with economic benefits
- ◯ Unemployment without financial benefits
- ◯ Retired (excluding partial retirements)
- ◯ Student
- ◯ Autonomous
- ◯ Other situation, specify: ____

Monthly salary received through professional activity:

- ◯ Less than 710,1€
- ◯ Between 710,1€ and 1.002,7€
- ◯ Between 1.002,8€ and 1.229,2
- ◯ Between 1.229,3€ and 1.412,6€
- ◯ Between 1.412,7€ and 1.594,4€
- ◯ Between 1.594,5€ and 1.824,0€
- ◯ Between 1.824,1€ and 2.137,4€
- ◯ Between 2.137,5€ and 2.595,0€
- ◯ Between 2.595,1€ and 3.316,8€
- ◯ 3.316,9€ or more euros

QUESTIONNAIRE ON THE STATE OF HEALTH: QUESTIONS ABOUT MEDICAL HISTORY		
	YES	NO
Does your child use glasses to read or work on the computer?		
Does your child suffer from any developmental or mental or psychiatric disorder that may interfere with his/her ability to learn? (e.g., attention deficit disorder, hyperactivity, dyslexia, etc.)<br><br>If you answer YES, indicate what the disorder is and the year it was diagnosed:		
Is your child taking any medication prescribed by the doctor/psychiatrist on a regular basis?<br><br>If your answer is YES, indicate the following information:<br>• Reason for the prescription:<br><br>• Prescribed medication:<br><br>• Quantity and frequency of each prescribed medication:		
Are there family antecedents of "Developmental Coordination Disorder"?<br><br>If your answer is YES, explain the diagnosis and proximity of the family member:		
Is there a family history of any psychological or psychiatric disorder?<br><br>If your answer is YES, explain the diagnosis and proximity of the		

family member:		

QUESTIONNAIRE ON THE STATE OF HEALTH: PHYSICAL ACTIVITY READINESS QUESTIONNAIRE		
	YES	NO
Has any doctor told you that your child has heart problems and should only do physical activities recommended by a doctor?		
Does your child have chest pain when doing any physical activity?		
Does your child lose balance due to dizziness, or has your child ever lost consciousness?		
Does your child have any bone or joint problems that could be aggravated by a change in physical activity?		
Does your child suffer from uncontrolled asthma (e.g. asthma that is not easily controlled with the use of an inhaler)?		
Is your child taking medications prescribed by the doctor for blood pressure or for the heart (for example, diuretic pills)?		
Do you know of any other reason against your child exercising? (Including diabetes, a recent serious injury or illness)		

PHYSICAL ACTIVITY QUESTIONNAIRE FOR CHILDREN (PAQ-C)

We want to know your level of physical activity in the last 7 days (last week). This includes all those activities such as sports, gymnastics or dance that make you sweat or feel tired, or games that make you breathe faster such as playing tag, jumping rope, running, climbing and others.

Remember:

1- There are no right or wrong answers. This is NOT a test.
2- Answer the questions in the most honest and sincere way possible. This is very important.

---

1. Physical Activity in your free time: Have you done any of these activities in the last 7 days (last week)? If your answer is yes: How many times have you done it? (*Mark only one option per activity/line*)

	None	1-2	3-4	5-6	7 times or +
jump rope					
Skate					
Play games like tag					
Bicycling					
Walking (as exercise)					
running/jogging					
Artistic gymnastics					
Rhythmic gymnastics					
Swimming					
dance/dance					
Badminton					
Rugby					
skateboarding					
Soccer/Futsal					
Volleyball					
Hockey					
Basketball					
Skiing					
Other racket sports					
Handball					
Athletics					
Martial arts (judo, karate, ...)					
Other:____________________________					

2. In the last 7 days, during physical education classes, how many times were you very active during classes (playing hard, running, jumping, throwing)? (Check only one option)

I did/do not do physical education	
Hardly ever	
Sometimes	
Often	
Always	

3. In the last 7 days, what did you usually do during recess? (Check only one option)

Sitting (talking, reading, class work)	
Being or walking around	
run or play a little	
run and play a lot	
Running and playing hard all the time	

4. In the last 7 days, what did you usually during the lunch break (before and after eating)? (Check only one option)

Sitting (talking, reading, class work)	
Being or walking around	
run or play a little	
run and play a lot	
Running and playing hard all the time	

5. In the last 7 days, right after school until 6 pm, on how many days did you play any games, do sports or dances that you were very active in? (Check only one option)

None	
1 time in the last week	
2-3 times in the last week	
4 times in the last week	
5 times in the last week	

6. In the last 7 days, on how many days from mid-afternoon (between 6 pm and 10 pm) did you do sports, dance, or play games in which you were very active? (Check only one option)

None	
1 time in the last week	
2-3 times in the last week	
4-5 times in the last week	

6-7 times in the last week	

7. Last weekend, how many times did you do sports, dance, or play games in which you were very active? (Check only one option)

None	
1 time	
2-3 times	
4-5 times	
6 times	

8. Which of the following phrases best describe your last week? Read all five before deciding which one best describes you. (Check only one option)

All or most of my free time was spent in activities that require little physical effort	
Sometimes (1-2 times a week) I did physical activities in my free time (for example, playing sports, running, swimming, cycling, aerobics)	
I often (3-4 times a week) did physical activity in my free time	
Quite often (5-6 times in the last week) I did physical activity in my free time	
Very often (7 or more times in the last week) I did physical activity in my free time	

9. Indicate how often you did physical activity for each day of the week (such as playing sports, playing, dancing or any other physical activity) (Little= between 20 and 30 minutes, Normal= between 30 minutes and 1 hour, Quite= between 1 hour and 2 hours, A lot = more than 2 hours).

	None	A little	Normal	Quite	A lot
Monday					
Tuesday					
Wednesday					
Thursday					
Friday					
Saturday					
Sunday					

10. Were you sick in the last week or did something to prevent you from doing normal physical activities?

Yes	
No	

In case your answer is "Yes", what happened to you?_________________________

CHILDREN'S MOVEMENT ASSESSMENT BATTERY BEHAVIORAL OBSERVATION CHECKLIST -2

Complete sections A and B before completing the overall assessment in the final table on the next page.

Section A. Moving in a static and/or predictable environment

0= Very good 1= Good 2= Regular 3= With great difficulty NO= Not observed	
**A.1 Personal autonomy**	
A.1.1 - Maintains balance while standing by putting on clothing (eg pants, skirt, sweater, etc.).	
A.1.2 - Puts the clothes over the head (ex, t-shirts, jumpers).	
A.1.3 - Buttons are done (ex, shirt, jacket).	
A.1.4 – Washes and dries hands	
A.1.5 – Pour liquids from one container to another (ex, from a pitcher to a glass).	
**A.2 Skills in the classroom**	
A.2.1 – Manipulates small objects (ex, tokens, necklace beads, sheets of paper).	
A.2.2 – Write letters using pencil or pen	
A.2.3 – Use scissors to cut paper.	
A.2.4 – Walk around the class avoiding immobile objects or people	
A.2.5 – Transports objects (ex, books, pencil cans) from one place to another without dropping them.	
**A.3 Sports and recreational skills**	
A.3.1 – Jump keeping both feet together when launching and landing on the ground.	
A.3.2 – Jumps on one leg with either foot.	
A.3.3 – Throw a beanbag or a ball so that another child who is standing still can catch it.	
A.3.4 – Use the fixed equipment of the gym/playground (ex, bars, slides).	
A.3.5 – Cross the gym/playground avoiding colliding with immobile objects or people.	
**SECTION A: TOTAL**	

Additional information (optional)

Please indicate if the child has learning difficulties ☐

and/or difficulties in: attention ☐ speaking ☐ reading ☐

social relationships ☐ emotional control ☐

Section B. Moving in a dynamic and/or unpredictable environment

0= Very good 1= Good 2= Regular 3= With great difficulty NO= Not observed	
**B.1 Personal Autonomy/Classroom Skills**	
B.1.1 – Maintains balance when frequent postural adjustments are required (ex, sitting on a bench in time with other children, staying in a line with other children on the move).	
B.1.2 – Moves through a crowded class picking up or distributing objects (ex, books, pencils).	
B.1.3 – Carry a tray or drink from one place to another avoiding moving people (ex, in the dining room)	
B.1.4 – Keep time with the music by clapping or tapping your foot on the floor	
B.1.5 – Moves the body to the rhythm of the music or to the rhythm of other people (ex, dance and rhythmic group activities).	
**B.2 Skills with ball**	
B.2.1 – Catches a ball using both hands	
B.2.2 – Hit a moving ball using a racket	
B.2.3 – Throws a ball while it is moving so that another child can catch it.	
B.2.4 – Repeatedly dribble a ball in a controlled manner.	
B.2.5 – Participate in collective games using throwing, catching, kicking or hitting skills.	
**B.3 Sports and recreational skills**	
B.3.1 – Ride a bicycle without <<training wheels>>.	
B.3.2 – Participates in games of chase and tag (ex, tag)	
B.3.3 – Maintains balance in water when with other children (ex, in the pool)	
B.3.4 – Use the mobile gym/playground materials (ex, hoops, scooters).	
B.3.5 – Crosses the gym/playground avoiding colliding with moving objects or people.	
**SECTION B: TOTAL**	

**Overall evaluation**			
**In general, do you think this child has movement difficulties?**			**Yes / No**
If so, are these difficulties negatively affecting the child? (mark with a circle)			
**School learning**	Not at all	A little	A lot
**Recreational activities**	Not at all	A little	A lot
**Self-esteem**	Not at all	A little	A lot
**social interactions**	Not at all	A little	A lot

Section C. Non-Motor Factors That May Affect Movement

	Yes	No
C.1 – **Disorganized** (ex, puts on shoes before socks		
C.2 – **Indecisive/Clueless** (ex, slow to initiate complex actions; forgets what to do in the middle of the task)		
C.3 – **Passive** (ex, no interest; needs a lot of encouragement to participate).		
C.4 – **Fearful** (ex, fearful of activities that require jumping or climbing; constantly asking for help).		
C.5 – **Anxious** (ex, trembles; becomes lightheaded in stressful situations)		
C.6 – **Impulsive** (ex, starts before finishing listening to instructions; impatient).		
C.7– **Distracted** (ex, looks around; responds to irrelevant noises).		
C.8 – **Hyperactive** (ex, fidgets and squirms; fidgets when given instructions; fiddles with clothing)		
C.9 – **Overestimates their abilities** (ex, tries to do more difficult tasks; tries to do things too fast).		
C.10 – **Underestimates their capabilities** (ex, complains that the task is difficult; anticipates failure before starting)		
C.11 – **Lack of persistence** (ex, gives up quickly; easily frustrated)		
C.12 – **Anger at failure** (ex, seems tearful; declines to try task again)		
C.13 – **Unable to derive satisfaction from success** (ex, does not respond to praise).		
Others (specify):		

Non-motor factors that can affect movement

(a) Do you think that the non-motor factors listed in section C of the Behavioral Observation Checklist prevent the child from demonstrating actual ability to move?

not at all / a little / a lot

(b) How do you think these difficulties will influence when planning the intervention program?

not at all / moderately / a lot
