# Supplementary material for: Study protocol to examine the effects of acute exercise on motor learning and brain activity in children with developmental coordination disorder (ExLe-Brain-DCD)
Source: PLoS One. 2024 May 9;19(5):e0302242. doi: 10.1371/journal.pone.0302242 (PMC11081356; doi:10.1371/journal.pone.0302242)
Supplement: Attachment — Submitted filename: Responses to Editor_and_reviewer.docx [file pone.0302242.s003.docx]

**Responses to Editor:**

1. Objective 1.2 and 1.3 (line 351 and onward) – the wording here is difficult to follow. Specifically the phrase “while performing…”. In objective1.2, the assessment of motor learning is done with this task so perhaps rephrase to “Compare motor learning (adaptation and consolidation) ability in children with and without DCD in a rotation visuo-motor task”. Similar changes can be made for Objective 1.3.

I strongly encourage the authors to refrain from stating global descriptors such as “visuomotor integration and attentional network areas” as part of the objectives (Objective 2). There are many areas that would fall under these labels (e.g., parietal cortex is known for sensorimotor integration and fronto-parietal networks are critically involved in attentional processes). Note also prefrontal cortices are involved in more than VM integration and attention (e.g., executive functioning). For clarity, it is advised to simply state the regions of interest that will be examined as part of the research. That is, readers know the specific regions examined. The portions of the manuscript that justify these regions can then include proposed functional roles.

Thank you for pointing out this issues and making some rephrasing proposals. Indeed, we agree with the editor that the objectives were not easy to read. We also understand and agree to your point about avoiding global descriptors and use instead specific cerebral areas. We have addressed all these issues in the revised version. We hope the introduced changes provide a clearer and easier understanding of our aims.

Please, see changes in lines 369 to 381 in the Revised Manuscript with Track Changes.

2. I would like to echo Reviewer 1 concerns with respect to “matching” across TD and DCD on this long list of factors. Children with DCD, on average, tend to be less active. So finding matched TD participants could add an extra burden that may compromise the completion of the research. And, if they are matched, I would imagine that this would mean the sampling of TD children is even less representative of the general population. Accordingly, it raises questions as to how generalizable the sample of TD children is. Although I recognize why this matching has been done, I simply wanted to extend Reviewer 1’s comments to highlight this issue further. Ultimately, I think a multitude of approaches can be justified here. So something for the research team to consider further before starting the experiment.

Since both, the editor and the reviewer, have raised their concerns regarding the proposed criteria for group matching in our study, we have reflected on its viability. After careful reconsideration, we agree that having so many matching criteria would be very difficult to handle. Therefore, we have modified our proposal so only three criteria will be used to match participant distribution among groups: age, sex, and handedness. Therefore, BMI (not weight), physical activity engagement, and physical fitness will be used as covariates if needed. That is, these three variables will be compared among groups (as well as age, sex, and handedness) to explore any group distribution difference and used as covariates in case group differences are found.

We want to thank the editor and the reviewer for this comment, as we think this is a very appropriate change to our study protocol. The initially proposed group matching could have possibly resulted in an unpractical and very difficult (or impossible) approach.

Please, see the modified sentence as lines 390-391 in the Revised Manuscript with Track Changes.

Journal requirements:

When submitting your revision, we need you to address these additional requirements.

1. Please ensure that your manuscript meets PLOS ONE's style requirements, including those for file naming. The PLOS ONE style templates can befound at

<https://journals.plos.org/plosone/s/file?id=wjVg/PLOSOne_formatting_sample_main_body.pdf> and

<https://journals.plos.org/plosone/s/file?id=ba62/PLOSOne_formatting_sample_title_authors_affiliations.pdf>

Done.

2. We note that the grant information you provided in the ‘Funding Information’ and ‘Financial Disclosure’ sections do not match.

When you resubmit, please ensure that you provide the correct grant numbers for the awards you received for your study in the ‘FundingInformation’ section.

Thank you to noticing this issue, we changed the text of the ‘Funding Information’ to match the text included in the ‘Financial Disclosure’ section (please, see the Cover letter).

3. Your ethics statement should only appear in the Methods section of your manuscript. If your ethics statement is written in any section besides the Methods, please move it to the Methods section and delete it from any other section. Please ensure that your ethics statement is included in your manuscript, as the ethics statement entered into the online submission form will not be published alongside your manuscript.

The ’Ethics Statement’ was already at the end of the ‘Materials and methods’ section (lines 697-727 in the Revised Manuscript with Track Changes). Please, let us know if it has to be moved anywhere else in the manuscript.

4. Thank you for stating the following in the Acknowledgments Section of your manuscript:

[We thank Dr. Priscila Tamplain (Developmental Motor Cognition Lab, Department of Kinesiology, University of Texas at Arlington, USA) and Dr. NadjaSchott (Institute of Sport and Exercise Science, University of Stuttgart, Germany) for their collaboration in the project. We also thank the Grup deRecerca en Activitat Física, Alimentació i Salut (GRAFAiS, Generalitat de Catalunya 2021SGR/01190) for their support.]

We note that you have provided funding information that is not currently declared in your Funding Statement. However, funding information shouldnot appear in the Acknowledgments section or other areas of your manuscript. We will only publish funding information present in the FundingStatement section of the online submission form.

The support of the Grup de Recerca en Activitat Física, Alimentació i Salut (GRAFAiS, Generalitat de Catalunya 2021SGR/01190) was only administrative. We clarified that in the ‘Acknowledgments’ sections (line 808 in the Revised Manuscript with Track Changes).

Please remove any funding-related text from the manuscript and let us know how you would like to update your Funding Statement. Currently, yourFunding Statement reads as follows:

[AB, BF, FB, and RA as authors of this study that is part of the R+D+i project PID2020-120453RB-I00 received funding from the Ministerio de Cienciae Innovación – Agencia Estatal de Invenstigación (https://www.aei.gob.es/; MCIN/AEI/10.13039/501100011033/). In addition, MA earned a PhDfellowship funded by the Institut Nacional d'Educació Física de Catalunya (INEFC) of the Generalitat de Catalunya(https://inefc.gencat.cat/es/inefc_barcelona/). The funders had and will not have a role in the study design, data collection and analyses, decision to publish, or preparation of the manuscript.]

Please include your amended statements within your cover letter; we will change the online submission form on your behalf.

We revised the manuscript looking for any funding-related text. No section contains funding-related texts in the new version of the manuscript. We removed the funding information from the Title page of the manuscript and now it is available in the cover letter.

5. Please review your reference list to ensure that it is complete and correct. If you have cited papers that have been retracted, please include therationale for doing so in the manuscript text, or remove these references and replace them with relevant current references. Any changes to thereference list should be mentioned in the rebuttal letter that accompanies your revised manuscript. If you need to cite a retracted article, indicate thearticle’s retracted status in the References list and also include a citation and full reference for the retraction notice.

Done.

**Responses to reviewers:**

1. The introduction is rather lengthy and somewhat repetitive. The text can be condensed. E.g. I do appreciate that the authors provide a comprehensive overview of DCD and its consequences, but this information can be summarized and the reader can be referred to some excellent recent review papers. Given that the many authors refer to DSM V (APA, 2013) it seems to make sense to refer to this manuscript too.

We agree with the reviewer that the introduction is long and some parts provide too much, and somewhat redundant, detail. In agreement with this comment, we have made an effort to shorten the introduction section, summarizing those parts where too much detailed information was provided, and deleting some sentences that were not critical for the justification of our study protocol.

We hope the implemented changes improve the focus of the introduction and provide a sufficiently comprehensive review of the literature background of our research protocol.

Please, see the introduction section of the revised manuscript were many changes have been performed in each of the pre-existing paragraphs.

Specific minor comments

AbstractL39: Given that these effects are generally limited to (moderate to) vigorous physical activity I would suggest being specific here. Or to use physical exercise.

We agree with the reviewer. We have detailed that the effects are specific to moderate-to-vigorous physical exercise.

The new sentence is (lines 40-41 in the Revised Manuscript with Track Changes): *“Moderate-to-vigorous physical exercise has been proven to improve motor learning (adaptation and consolidation) in children with or without disorders.”*

L168: typo: prefrontal cortex' role

Thank you for noticing this typo. We have corrected it in the revised version of the manuscript. See line 175 in the Revised Manuscript with Track Changes.

L363 and 367: typically developing children is the preferred term (instead of developed)

Thank you for noticing this inconsistency. We have changed these two lines to be consistent with the most appropriate term (typically developing).

L376: the groups will be matched on age, gender, weight, handedness, PA engagement and fitness level. I understand why, but it seems to be very complex to match each and every child on 6 factors. Can the authors please explain how this will be done.

Please, see our comment to the editor in point 2 where we address this concern. Thank you for this important contribution to our protocol. Please, see the modified sentence as lines 390-391 in the Revised Manuscript with Track Changes.

L421: Given that the study will use matched control groups, allocation cannot be fully randomized, I think.

We agree with the reviewer. In our case, participant allocation will be partially randomized, not fully randomized. We have clarified this issue in the revised manuscript. Please, see the changes performed in lines 52 and 465-466 in the Revised Manuscript with Track Changes.

L602: What are the regions of interest and how will parcellation of the regions of interest be done?

Thank you for this comment, which has also been raised by the editor. We have modified the manuscript to specify that the two brain areas of interest are the dorsolateral pre-frontal cortex (DLPFC) and the ventrolateral pre-frontal cortex (VLPFC). Since we have a limitation of 27 channels in our current fNIRS system, we need to focus on the most relevant brain areas for our aims, population and experimental protocol, that is, the pre-frontal cortex. We also specifically indicate that the established 10-20 EEG protocol will be followed for the optodes placements so the targeted brain areas can be evaluated.

See lines 556-562 in the Revised Manuscript with Track Changes.

L636-638: Is this analysis necessary, given that the groups will be matched?

We understand the reviewer’s doubts on this statistical procedure, but we still think that an analysis is necessary for the following reason. Comparison of the group matching criteria (age, sex, and handedness) will be performed to corroborate that the participant allocation was performed correctly. Furthermore, since now we will not use BMI, physical activity engagement, and physical fitness as group distribution criteria, we need to check if there exist any group effect on these criteria and use them as covariates whenever necessary.

L699: Is it necessary to use sex as a covariate, given that the groups will be matched? Also, please note that the construct "gender" was used before.

Thank again for this comment. In this case the proposed analysis by sex reflects our interest to address an ethical consideration that although our efforts to balance groups by sex, there might still be a sex difference in some of our measures and we would like to know the answer to this question.

We have revised the manuscript to be consistent with the terminology “sex” to refer to the sex designation at birth, and the use of gender to address a more general term that could include sex at birth of other forms of defining “sex” designation.
