# Supplementary material for: Study protocol to examine the effects of acute exercise on motor learning and brain activity in children with developmental coordination disorder (ExLe-Brain-DCD)
Source: PLoS One. 2024 May 9;19(5):e0302242. doi: 10.1371/journal.pone.0302242 (PMC11081356; doi:10.1371/journal.pone.0302242)
Supplement: Attachment — Submitted filename: renamed_aace2.docx [file pone.0302242.s004.docx]

# COMMENTS – Manuscript PONE-D-23-24057R1

Title: “Study protocol to examine the effects of acute exercise on motor learning and brain activity in children with developmental coordination disorder (ExLe-Brain-DCD)”

**Important note: This review pertains only to ‘statistical aspects’ of the study and so ‘clinical aspects’ [like medical importance, relevance of the study, ‘clinical significance and** **implication(s)’ of the whole study, etc.] are to be evaluated [should be assessed] separately/independently. Further please note that any ‘statistical review’ is generally done under the assumption that (such) study specific methodological [as well as execution] issues are perfectly taken care of by the investigator(s). This review is not an exception to that and so does not cover clinical aspects {however, seldom comments are made only if those issues are intimately / scientifically related & intermingle with ‘statistical aspects’ of the study}. Agreed that** **‘statistical methods’ are used as just tools here, however, they are vital part of methodology [and so should be given due importance]. I look at the manuscript in/with statistical view point, other reviewer(s) look(s) at it with different angle so that in totality the review is very comprehensive. However, there should be efforts from authors side to improve (may be by taking clues from reviewer’s comments). Therefore, please do not limit the revision only (with respect) to comments made here.**

**COMMENTS:** There are quite a few issues (including few serious objections/observations) about which I have different opinion. Such observations/concerns are given below:

Firstly, I observed that your ABSTRACT [though drafted alright in my opinion], is ‘assay type’. It is preferable to divide the ABSTRACT with small sections like ‘Objective(s)’, ‘Methods’, ‘Results’, ‘Conclusions’, etc. which is an accepted practice of most of the good/standard journals [including this one, though ‘The PLoS One Guidelines to Authors’ did not specify an Abstract format, it is desirable]. It will definitely be more informative then, I guess, whatever the article type may be (including ‘protocol’).

As noted by some reviewer earlier, the introduction is lengthy [nearly occupies 38% of total length though I appreciate providing a comprehensive overview of DCD and its consequences]. Nearly 65% references (out of total 140) are related to the introduction. Can this be reduced further? (Assumed that you might have done it earlier). Further, I request these learned authors to kindly recheck the ‘Sample size calculation’ [lines 357-368] because {though ‘GPower’ is an excellent package} the results are different than well-known standard [table-2 on page 158 of Jacob Cohen’s paper “A power primer” in Psychological Bulletin, 1992, vol.:112, pp 155-159 (which is a sort of summary of the excellent book by Cohen himself titled ‘Statistical power analysis for the behavioral sciences’, Academic Press, 1977, New York)]. This is probably due to large ‘effect size’ assumed {The effect sizes (ES) in these studies were 0.78 and 0.55} whereas in fact for such intervention(s) the effect size found/noted is generally at-the-most be ‘medium’.

I have one basic doubt regarding the correctness of choice of a control group [for example, look at the statement made in lines 246-7: whether the children with DCD could benefit from this strategy to improve their motor learning capacity is not known]. From the account given in lines 44 to 46 {One hundred twenty children will be recruited (60 DCD, 60 controls) and within-cohort randomly assigned to either exercise (13-minute shuttle run task) or rest prior to performing the rVMA task} it is clear that cohort of 60 children with DCD will further be divided in two groups which is alright. But then I doubt about the ‘effect size’ assumed (very large & so the required n=120). Therefore, I again request authors to recheck the control group of those studies {line 357-359: Statistical power analyses were performed for the sample size estimation, based on initial directional error (IDE) data from Kagerer et al. [47] study (N=20) comparing TD to DCD and from Ferrer-Uris et al. [57] study (N=21) comparing EX and CON groups}. ‘What exactly you mean by “Statistical power analyses were performed for the sample size estimation”. Do we usually perform ‘Statistical power analyses for the sample size estimation’?

What is in lines 584-6 [Assumptions for the statistical tests will be checked before their application and necessary adjustments, corrections or supplementary calculations will be applied to increase the robustness of the results] is highly appreciated, however, I request authors to carefully read the following note which is pasted from one famous standard textbook on ‘Medical Research Methodology’ [though I am sure that the authors already know these things].

Please use suitable non-parametric test(s)/technique(s) while dealing with data that are in ‘ordinal’ level of measurement even if [despite that] the distribution may be ‘Gaussian’. Testing ‘normality’ in sample [by using any normality test(s)} is not required/desired while dealing with data that are in ‘ordinal’ level of measurement [as most of the normality tests are not valid for ‘ordinal’ data]. Agreed that there is/are no non-parametric test(s)/technique(s) available to be used as alternative in all situation(s), but should be used whenever/wherever they are available.

Note that though the measures/tools used are appropriate, most of them are likely to yield data that are in ‘ordinal’ level of measurement [and not in ratio level of measurement for sure {as the score two times higher does not indicate presence of that parameter/phenomenon as double (for example, a Visual Analogue Scales VAS score or say ‘depression’ score)}]. Then application of suitable non-parametric (or distribution free) test(s) is/are indicated/advisable.

Description in ‘Data analysis’ section is confusing in the sense that ‘how many groups data are dealt here’? If two groups, then why do you need ANOVA? How the measures are to be explored through one-way analysis of variance (ANOVA)? [refer to lines 559-561: Age, sex at birth, BMI, handedness, physical activity engagement, and fitness level (estimated VO2max) measures will be explored through one-way analysis of variance (ANOVA)]. What do you mean by exploring measures through ANOVA? Refer to lines 576-78 where stated that “Differences between the children groups (O1.2 and O1.3) on the motor learning and on the cortical activity (O2.1 and O2.2) through all sets will be explored using a General Lineal Mixed Model (GLMM) with repeated measures”. Are differences between the children groups explored using a General Lineal Mixed Model (GLMM) with repeated measures? How many times repeated observations you have? Please clarify all the aspects of ‘DESIGN’ of the study, first [very clearly]. Remember/mind you that this is a scientific/academic document and so all details should be clearly/correctly communicated (do not take readers’ for granted).

In lines 685-88, it is stated that “Recognizing the inherent limitations of this project, we may help teachers and parents in identifying those children at risk or high probability of presenting DCD by generating and distributing (via workshops) guidelines for the diagnosis of this disorder and proper interventions for these children”. Is that a part of this study/project? Does that mean {according to authors} there are no other {this is not a limitation of this study anyway}, limitation(s) of this study? As pointed out in ‘important note’ above “This review pertains only to ‘statistical aspects’ of the study and so ‘clinical aspects’ should be assessed separately/independently [one should carefully consider/look at the clinical implications of the study]. In my opinion, to make this article acceptable (which is possible though not easy), some amount of re-vision (re-drafting) may be needed. However, please do not limit the revision only (with respect) to comments made here.

The respected ‘Editor’ may consider accepting/further processing only if found ‘clinical implications’ valuable [i.e., add(s) to clinical knowledge or positively influence clinical practice]. ‘Major revision’ is recommended.
