# Supplementary material for: Study protocol to examine the effects of acute exercise on motor learning and brain activity in children with developmental coordination disorder (ExLe-Brain-DCD)
Source: PLoS One. 2024 May 9;19(5):e0302242. doi: 10.1371/journal.pone.0302242 (PMC11081356; doi:10.1371/journal.pone.0302242)
Supplement: Attachment — Submitted filename: Responses to Reviewers.docx [file pone.0302242.s005.docx]

**Responses to Editor:**

We really appreciate your efforts to find a statistical reviewer and highlight the most important issues to consider for this review. We did our best to answer the reviewer’s questions and to include his/her suggestions in the new manuscript version.

To ensure we covered all the reviewer’s comments that you highlighted, we organized our answers following the topics described in the four bullets you presented to us.

**Responses to reviewers:**

Firstly, I observed that your ABSTRACT [though drafted alright in my opinion], is ‘assay type’. It is preferable to divide the ABSTRACT with small sections like ‘Objective(s)’, ‘Methods’, ‘Results’, ‘Conclusions’, etc. which is an accepted practice of most of the good/standard journals [including this one, though ‘The PLoS One Guidelines to Authors’ did not specify an Abstract format, it is desirable]. It will definitely be more informative then, I guess, whatever the article type may be (including ‘protocol’).

Thank you for considering the abstract of the manuscript well drafted. Following your advice, we divided the abstract into sections but adapting the sections to the type of article (i.e., Study Protocol article) and looking at how other articles in PLoS One have done it (for example: Sun, F. et al., 2022, and Tse, A.C.Y, et al, 2022). Therefore, the abstract is divided into “Introduction”, “Objectives”, “Methods”, and “Discussion” (pages 2-3, lines 31-58 of the new manuscript version).

As noted by some reviewer earlier, the introduction is lengthy [nearly occupies 38% of total length though I appreciate providing a comprehensive overview of DCD and its consequences]. Nearly 65% references (out of total 140) are related to the introduction. Can this be reduced further? (Assumed that you might have done it earlier).

We revised again the ‘Introduction’ section to shorten its length (see page 6 and 8 of the new manuscript version). Although the ‘Introduction’ may still appear lengthy and with many references, we think that the synthesized information we present is essential to provide the reader with the necessary reasons that justify the study and its design.

Further, I request these learned authors to kindly recheck the ‘Sample size calculation’ [lines 357-368] because {though ‘GPower’ is an excellent package} the results are different than well-known standard [table-2 on page 158 of Jacob Cohen’s paper “A power primer” in Psychological Bulletin, 1992, vol.:112, pp 155-159 (which is a sort of summary of the excellent book by Cohen himself titled ‘Statistical power analysis for the behavioral sciences’, Academic Press, 1977, New York)]. This is probably due to large ‘effect size’ assumed {The effect sizes (ES) in these studies were 0.78 and 0.55} whereas in fact for such intervention(s) the effect size found/noted is generally at-the-most be ‘medium’.

I have one basic doubt regarding the correctness of choice of a control group [for example, look at the statement made in lines 246-7: whether the children with DCD could benefit from this strategy to improve their motor learning capacity is not known]. From the account given in lines 44 to 46 {One hundred twenty children will be recruited (60 DCD, 60 controls) and within-cohort randomly assigned to either exercise (13-minute shuttle run task) or rest prior to performing the rVMA task} it is clear that cohort of 60 children with DCD will further be divided in two groups which is alright. But then I doubt about the ‘effect size’ assumed (very large & so the required n=120). Therefore, I again request authors to recheck the control group of those studies {line 357-359: Statistical power analyses were performed for the sample size estimation, based on initial directional error (IDE) data from Kagerer et al. [47] study (N=20) comparing TD to DCD and from Ferrer-Uris et al. [57] study (N=21) comparing EX and CON groups}. ‘What exactly you mean by “Statistical power analyses were performed for the sample size estimation”. Do we usually perform ‘Statistical power analyses for the sample size estimation’?

*Answer to the comment on the choice of the control group as it pertains to the design*

The study protocol presented in our manuscript has two main objectives: (1) To study acute intense exercise effects on motor learning (adaptation and consolidation) in children with and without DCD from a behavioral perspective (performance and execution), and (2) To explore brain activity in the dorsolateral pre-frontal cortex (DLPFC) and ventrolateral pre-frontal cortex (VLPFC) while learning a motor adaptation task in children with and without DCD, under rest or post-exercise conditions. (Please, see the specific objectives detailed in pages 12-13 lines 276-303 of the new manuscript version). On that account, it is important to point that there are two main factors that impact the study design to fulfill these objectives: the disorder condition (if children have or not DCD), and the performance of the acute exercise intervention (if children perform or not acute intense exercise before the rotational visuomotor task).

Regarding the disorder condition, some previous articles studied the motor learning capacity in children with DCD contrasting them to typically develop children from a behavioral perspective (for example, Kagerer et al. 2004; Kagerer et al., 2006; King et al., 2011) but there are no studies to our knowledge that provided evidence of the prefrontal cortex (PFC) activity to explore possible differences in the brain responses of the DCD and TD children during a motor learning task. Furthermore, the existing research literature has not examined the PFC activity and its contribution on rotational visuomotor adaptation tasks in children.

Regarding the acute exercise effect on motor learning in children, very few articles examined the behavioral changes during adaptation and consolidation phases in children with TD (Lundbye-Jensen et al., 2017; Ferrer-Uris et al., 2018; Angulo-Barroso et al., 2019). No studies focusing on children including concurrent PFC activity and behavioral assessments have been published yet, and much less in children with DCD.

Therefore, the inclusion of the two groups of TD children (CON-TD and EX-TD) in our study design is important to properly draw robust results not only about the impact of the acute exercise on motor learning in children with and without DCD, but also regarding the possible underlying mechanisms related to the PFC that are implicated on a visuomotor adaptation task in children. That way, and by including the four groups in our experimental design (CON-TD, CON-DCD, EX-TD, and EX-DCD), we will be able to answer the questions explicitly exposed in the specific objectives:

- CON-TD vs CON-DCD: (O1.2.) Compare motor learning (adaptation and consolidation) ability in children with and without DCD while performing a rotational visuo-motor task (rVMA); and (O2.1) assess changes in DLPFC and VLPFC activity ([02Hb]) during rVMA learning (adaptation and consolidation) in typically developing children and in children with DCD.
- CON-TD vs EX-TD, CON-DCD vs EX-DCD and EX-TD vs EX-DCD: (O1.3) Examine the effect of acute intense exercise on motor learning (adaptation and consolidation) ability in children with and without DCD while performing a rotational visuo-motor task; and (O2.2) Evaluate the impact of acute intense exercise on changes in DLPFC and VLPFC activity ([02Hb]) during rVMA learning (adaptation and consolidation) in typically developing children and in children with DCD.

*Answer to the comment on the effect size and size of the proposed sample*

Power analysis is normally conducted before the data collection. The main purpose underlying power analysis is to help the researcher to determine the smallest sample size that is suitable to detect the effect of a given test at the desired level of significance. Therefore, power analysis is a critical step for study design to determine the appropriate sample size. This process requires the determination of effect sizes from relevant publications.

To conduct the power analysis and obtain a recommended sample size regarding comparisons between TD and DCD or between EX and CON, we used initial directional error (IDE) data from Kagerer et al. (2006) and Ferrer-Uris et al. (2018) (both studies mentioned in the previous point since the former utilized children with DCD and the later included acute exercise impact on motor learning in TD children).

As the reviewer suggested, we recalculated the effect sizes and estimated the sample size following Cohen’s paper (Cohen, 1992). In the next table, the reviewer can see all the data used to calculate the effect size values (now expressed as Cohen’s d) and the sample size estimated using both methods GPower and Cohen’s tables. The results of the sample size estimation are similar despite the method used.

To clarify the calculations made about the sample size and ensure their reproducibility using GPower or the Cohen’s publications, we included the data showed in the previous table in the ‘Sample size calculation’ sub-section of the new version of the manuscript (page 15-16, lines 366-375).

What is in lines 584-6 [Assumptions for the statistical tests will be checked before their application and necessary adjustments, corrections or supplementary calculations will be applied to increase the robustness of the results] is highly appreciated, however, I request authors to carefully read the following note which is pasted from one famous standard textbook on ‘Medical Research Methodology’ [though I am sure that the authors already know these things].

Please use suitable non-parametric test(s)/technique(s) while dealing with data that are in ‘ordinal’ level of measurement even if [despite that] the distribution may be ‘Gaussian’. Testing ‘normality’ in sample [by using any normality test(s)} is not required/desired while dealing with data that are in ‘ordinal’ level of measurement [as most of the normality tests are not valid for ‘ordinal’ data]. Agreed that there is/are no non-parametric test(s)/technique(s) available to be used as alternative in all situation(s), but should be used whenever/wherever they are available.

Note that though the measures/tools used are appropriate, most of them are likely to yield data that are in ‘ordinal’ level of measurement [and not in ratio level of measurement for sure {as the score two times higher does not indicate presence of that parameter/phenomenon as double (for example, a Visual Analogue Scales VAS score or say ‘depression’ score)}]. Then application of suitable non-parametric (or distribution free) test(s) is/are indicated/advisable.

*Answer to the comment on the type of data (potentially ordinal)*

Thank you for pointing out the need to indicate the non-parametric test we will use in case of ordinal data. It is important to note that all data obtained to contrast the motor learning capacity of the DCD and TD children and the effect of performing exercise (specific objectives: O1.2, O1.3, O2.1, O2.2., and O2.3) are in ratio level of measurements (concretely they are continuous data). The rotational visuomotor adaptation task (rVMA) in our protocol will be conducted in a computer by moving a joystick and coordinates of the joystick movement (in X and Y axes) will be recorded at 120 Hz. The IDE, RMSE, movement time, reaction time, travel distance, and the rate learning variables can be then calculated from these X and Y coordinates for each trial. The brain activity change during the rVMA will be described by the relative change of the oxyhemoglobin and deoxyhemoglobin measures done by the fNIRS at 50 Hz. The other variables will be used statistically to describe the groups (specific objective O1.1). In the next table, the reviewer can see all the variables, their units, and the data level of measurement.

*Answer to the choice of statistical test (parametric vs. non parametric)*

We agreed with the reviewer that no exploration for normal distribution will be need for categorical and ordinal data (i.e., sex at birth, handedness, PAQ-C score, M-ABC2 score, Conners test score, and TONI-4 score) and specific non-parametric test has to be selected beforehand. Crosstab statistics will be use to explore the differences between groups of sex and handedness (categorical variables), while Kruskal-Wallis tests will be conducted to identify group differences in PAQ-C, M-ABC2, Conners test, and TONI-4 scores (ordinal variables). We clarify the use of these tests in the new version of the manuscript (page 23-24, lines 576-580 and 586-587).

Description in ‘Data analysis’ section is confusing in the sense that ‘how many groups data are dealt here’? If two groups, then why do you need ANOVA? How the measures are to be explored through one-way analysis of variance (ANOVA)? [refer to lines 559-561: Age, sex at birth, BMI, handedness, physical activity engagement, and fitness level (estimated VO2max) measures will be explored through one-way analysis of variance (ANOVA)]. What do you mean by exploring measures through ANOVA? Refer to lines 576-78 where stated that “Differences between the children groups (O1.2 and O1.3) on the motor learning and on the cortical activity (O2.1 and O2.2) through all sets will be explored using a General Lineal Mixed Model (GLMM) with repeated measures”. Are differences between the children groups explored using a General Lineal Mixed Model (GLMM) with repeated measures? How many times repeated observations you have? Please clarify all the aspects of ‘DESIGN’ of the study, first [very clearly]. Remember/mind you that this is a scientific/academic document and so all details should be clearly/correctly communicated (do not take readers’ for granted). Kindly check for the ‘English’ language. Agreed that English is not our mother tongue (definitely not mine, may or may not be yours but certainly not of many readers).

*Answer to the comment on the data analysis with respect to the number of groups and the type of test used (i.e., ANOVA vs. t-tests)*

In our study protocol, we include two cohorts of children (children with DCD and children TD) that are assigned using partial-within cohort randomization to an exercise group (EX, children who will perform exercise before the motor learning task) or a control group (CON, children who will not perform exercise before the motor learning task). Therefore, we have in total four groups: CON-DCD, CON-TD, EX-DCD, and EX-TD. Please, see the ‘Study design and setting’ section (page 13 lines 306-309), the ‘Participants’ section (page 15, lines 355-360), the ‘Statistical size calculation’ section (page 16, lines 375-376), or the ‘Data analysis’ section (page 24, lines 584-587).

In this case when four groups are defined (2 cohort (DCD, TD) and 2 exercise (EX, CON)), applying multiple t-tests will not be correct and would induce type I errors. Typically for ratio data, two-way ANOVA with appropriate corrections (e.g., Greenhouse-Geisser, Bonferroni) is the adequate statistical analysis to inform about the main effect of each factor (i.e., cohort and exercise) and the interaction of both factors (possible differences between the four independent groups: CON-DCD, CON-TD, EX-DCD, and EX-TD). Alternatively, cross-tabulations (categorical data) and Kruskal-Wallis test (ordinal data) are more appropriate to compare groups with non-parametric measures.

*Answer to the comments with respect to clarity of the design (e.g., clarifying number of observations).*

Thank you for pointing some issues detected in the ‘Data analysis’ section. We modified the text to clarify all the detected problems and following the statistical analyses summarized in the next table.

Statistics in the study protocol are organized similarly to the table, that is, per objectives:

- The first paragraph presented the statistical test that will be conducted during participants’ allocation to ensure that this allocation of participants does not create initial group differences that could impact on the results of the intervention. In the new version of the manuscript, we corrected the number of ways (factors) of the ANOVA and improved the text to clarify the objective of the statistic test and to present equivalent non-parametric tests for categorical and ordinal variables (page 23-24, lines 574-581).
- The second paragraph describes the statistical tests applied to characterize the four groups (descriptive statistics) and expose differences between them (2 x 2 ANOVA). The number of groups and the Kruskal-Wallis test for ordinal data has been added in the new version of the manuscript to compare the four groups (page 24, lines 585-588).
- The third paragraph introduced the adaptation set division in three equal parts to obtain subsets with time durations equal to the retention sets lengths. Next, we linked the specific objectives O1.2 and O1.3 with the behavioral data, while the fNIRS data are related to the objectives O2.1 and O2.2. We presented GLMM with repeated measures through all sets (AD1, AD2, AD3, RT1h, RT24h, RT7d) as statistical test to detect differences between the four groups (Stroup, 2012; West, Welch, & Galecki, 2014). GLMMs will allow us to consider the inter-trial variability (subject as possible random effect) in the analyses and deal with the missing values (high probable for the fNIRS data). Finally, we described the statistics to analyze associations between behavior and PFC activity (O2.3) for discrete (bivariate correlations) and continuous variables (cross-correlations). The list of the sets has been written down to ensure that the reader knows the number of repeated measures (page 24, lines 598-599).

References:

Stroup, W.W. (2012). Generalized Linear Mixed Models: Modern Concepts, Methods and Applications (1st ed.). CRC Press. https://doi.org/10.1201/b13151

West, B.T., Welch, K.B., & Galecki, A.T. (2014). Linear Mixed Models: A Practical Guide Using Statistical Software, Second Edition (2nd ed.). Chapman and Hall/CRC. https://doi.org/10.1201/b17198

In lines 685-88, it is stated that “Recognizing the inherent limitations of this project, we may help teachers and parents in identifying those children at risk or high probability of presenting DCD by generating and distributing (via workshops) guidelines for the diagnosis of this disorder and proper interventions for these children”. Is that a part of this study/project? Does that mean {according to authors} there are no other {this is not a limitation of this study anyway}, limitation(s) of this study?

We think that an important outcome of a project is the transfer of the knowledge acquired during this research to the general public (i.e., parents and teachers). In the lines cited by the reviewer, our intention was to emphasize that this return to the society has to be done with caution to prevent overreaching the conclusions summarized in the guidelines by being conservative and recognizing the limitations of the research. We clarified this position in the new version of the manuscript (page 28, lines 708-713).
